# Efficient lipidomic approach for the discovery of lipid ligands for immune receptors by combining LC-HRMS/MS analysis with fractionation and reporter cell assay

**DOI:** 10.1007/s00216-023-05111-w

**Published:** 2023-12-23

**Authors:** Noriyuki Tomiyasu, Masatomo Takahashi, Kenji Toyonaga, Sho Yamasaki, Takeshi Bamba, Yoshihiro Izumi

**Affiliations:** 1 Department of Systems Life Sciences, Graduate School of Systems Life Sciences, Kyushu University, Fukuoka, Japan; 2 Division of Metabolomics/Mass Spectrometry Center, Medical Research Center for High Depth Omics, Medical Institute of Bioregulation, Kyushu University, Fukuoka, Japan; 3 Department of Molecular Immunology, Research Institute for Microbial Diseases, Osaka University, Osaka, Japan; 4 Section of Infection Biology, Department of Functional Bioscience, Fukuoka Dental College, Fukuoka, Japan; 5 Laboratory of Molecular Immunology, Immunology Frontier Research Center (IFReC), Osaka University, Osaka, Japan

**Keywords:** Lipidomics, LC-HRMS/MS, Reporter cell assay, Microfractionation, Lipid ligand, Immune receptor

## Abstract

C-type lectin receptors (CLRs), which are pattern recognition receptors responsible for triggering innate immune responses, recognize damaged self-components and immunostimulatory lipids from pathogenic bacteria; however, several of their ligands remain unknown. Here, we propose a new analytical platform combining liquid chromatography-high-resolution tandem mass spectrometry with microfractionation capability (LC-FRC-HRMS/MS) and a reporter cell assay for sensitive activity measurements to develop an efficient methodology for searching for lipid ligands of CLR from microbial trace samples (crude cell extracts of approximately 5 mg dry cell/mL). We also developed an in-house lipidomic library containing accurate mass and fragmentation patterns of more than 10,000 lipid molecules predicted in silico for 90 lipid subclasses and 35 acyl side chain fatty acids. Using the developed LC-FRC-HRMS/MS system, the lipid extracts of *Helicobacter pylori* were separated and fractionated, and HRMS and HRMS/MS spectra were obtained simultaneously. The fractionated lipid extract samples in 96-well plates were thereafter subjected to reporter cell assays using nuclear factor of activated T cells (NFAT)-green fluorescent protein (GFP) reporter cells expressing mouse or human macrophage-inducible C-type lectin (Mincle). A total of 102 lipid molecules from all fractions were annotated using an in-house lipidomic library. Furthermore, a fraction that exhibited significant activity in the NFAT-GFP reporter cell assay contained α-cholesteryl glucoside, a type of glycolipid, which was successfully identified as a lipid ligand molecule for Mincle. Our analytical platform has the potential to be a useful tool for efficient discovery of lipid ligands for immunoreceptors.

**Graphical Abstract:**

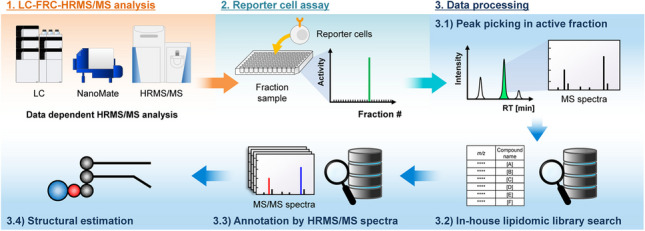

**Supplementary Information:**

The online version contains supplementary material available at 10.1007/s00216-023-05111-w.

## Introduction

Various natural products have a wide range of biological roles through their interactions with the host [1]. In our bodies, small molecules (metabolites) also interact with some receptors, such as G protein–coupled receptors (GPCRs) [2]. Other functions of metabolites include regulation of gene expression, transcription, and protein activity [3–5]. Liquid chromatography-mass spectrometry (LC–MS) and liquid chromatography-tandem mass spectrometry (LC–MS/MS) are widely used to detect bioactive compounds [6–8]. Recent advances in analytical instrumentation have led to the development of ultra-high-performance liquid chromatography (UHPLC), which can control mobile phase delivery at high pressures (approximately 130 MPa). This advantage allows the use of packed columns with a particle size of less than 2 µm, enabling short analysis times while maintaining high separation efficiency [9]. Improvements in electrospray ionization (ESI), ion transmission, and detectors have enabled sensitive detection of a wide range of metabolites [8]. In addition, the rapid development of high-resolution tandem mass spectrometry (HRMS/MS), such as quadrupole time-of-flight mass spectrometry (high mass accuracy, < 5 ppm; and high mass resolution, *m*/Δ*m* =  > 40,000), quadrupole Orbitrap mass spectrometry (< 1 ppm and *m*/Δ*m* =  > 120,000), and Fourier transform ion cyclotron resonance mass spectrometry (< 1 ppb and *m*/Δ*m* =  > 1,000,000), enables the detection of trace-level compounds with high sensitivity and resolution [10]. Thus, the use of UHPLC and HRMS/MS facilitated the comprehensive analysis of small-molecule compounds. Although several bioactive compounds such as metabolites that act as ligands for receptor are believed to exist in nature [11, 12], methodologies for the rapid and accurate identification of these compounds are not well established.

Lipids play important roles in biology as energy storage molecules, cell membrane components, and signaling molecules [13–15]. Currently, LIPID MAPS [16] classifies lipid molecules into 301 lipid subclasses based on their structures. There are approximately 100,000 lipid species in nature, including in silico predicted structures, and the structural diversity of lipid molecules drives diverse biological functions [17]. Lipids have also been implicated in innate immunity [18–20]. Innate immunity is a mechanism in the early stages of the host defense response, whereby immune cells such as dendritic cells and macrophages, which are found in the blood and various tissues, recognize a unique composition of pathogens and eliminate them. C-type lectin receptors (CLRs), widely known as pattern recognition receptors, recognize pathogen-specific lipid molecules and activate the immune responses [18–20]. Some pathogens evade recognition by immune receptors [21] or induce an overactive immune system [22], leading to severe infections. However, there are still orphan receptors whose ligands are unknown [11], and the identification of their ligands is essential for elucidating the mechanisms of immune responses. Lipid ligands possessed by pathogens are useful for the development of inhibitors of enzymes that synthesize and degrade lipid molecules as therapeutic agents for infectious diseases. More than 800 receptors are known to exist in the human body, of which approximately 100 are orphan GPCRs [12]. In addition, some lipid ligands that regulate biological functions may only be present in the human body at significantly low concentrations (a few hundred nM) [23, 24]. For instance, serum concentrations of lysophosphatidic acid (LPA), which induces cell proliferation, cell migration, and other functions via LPA receptors, range from 0.14 to 1.6 μM [24]. The identification of such bioactive lipids and their receptors, which occur in trace amounts in living organisms and in nature, and the elucidation of their biosynthetic mechanisms are important not only for drug development but also for the elucidation of detailed mechanisms in Oriental medicine (e.g., Chinese herbal medicine).

Traditional studies on bioactive compounds, known as forward pharmacology, have been conducted for a long time. In this method, compounds are extracted from biological samples and added to the target cells or tissues to detect their activity. The active fraction was thereafter separated into smaller fractions for isolation and purification of the target compound. Structural analysis was performed to identify the bioactive compounds and their receptors [25]. However, whole-genome sequencing has revealed the presence of all receptors, including unidentified ligands [11, 12]. Therefore, reverse pharmacology is the most widely used method for drug discovery. In this method, a specific target receptor is identified in advance, candidate compounds that interact with this target receptor are investigated, and their pharmacological effects are elucidated [25]. However, most of the target compounds comprised less than 1% by weight of the crude extract [6]. Therefore, high-yield extraction and purification techniques for the targets in biological samples are essential. In addition, high sensitivity, simplicity, and repeatability are important for activity testing [26]. Traditional methods require large amounts of biological samples, cumbersome procedures, and considerable time and effort to identify novel receptor ligands. Therefore, there is a need to develop a system that enables high-throughput screening of novel ligands for known orphan receptors using small amounts of samples.

X-ray crystallography, nuclear magnetic resonance, and LC–MS have been used for the structural analysis of natural products. In particular, LC–MS is a useful analytical method for the structural determination of trace compounds (pg‒ng) owing to its excellent detection sensitivity. Therefore, we assumed that the structure of the small-molecule ligand for the receptor could be more efficiently determined (estimated) if high-sensitivity MS analysis, sampling for activity testing, and a high-sensitivity reporter cell assay could be performed using only a trace sample. Screening systems integrating bioassays and LC–MS have been developed and studied for the efficient discovery of enzyme inhibitors and receptor ligands, but reports are still scarce [27–29].

**Fig. 1:**
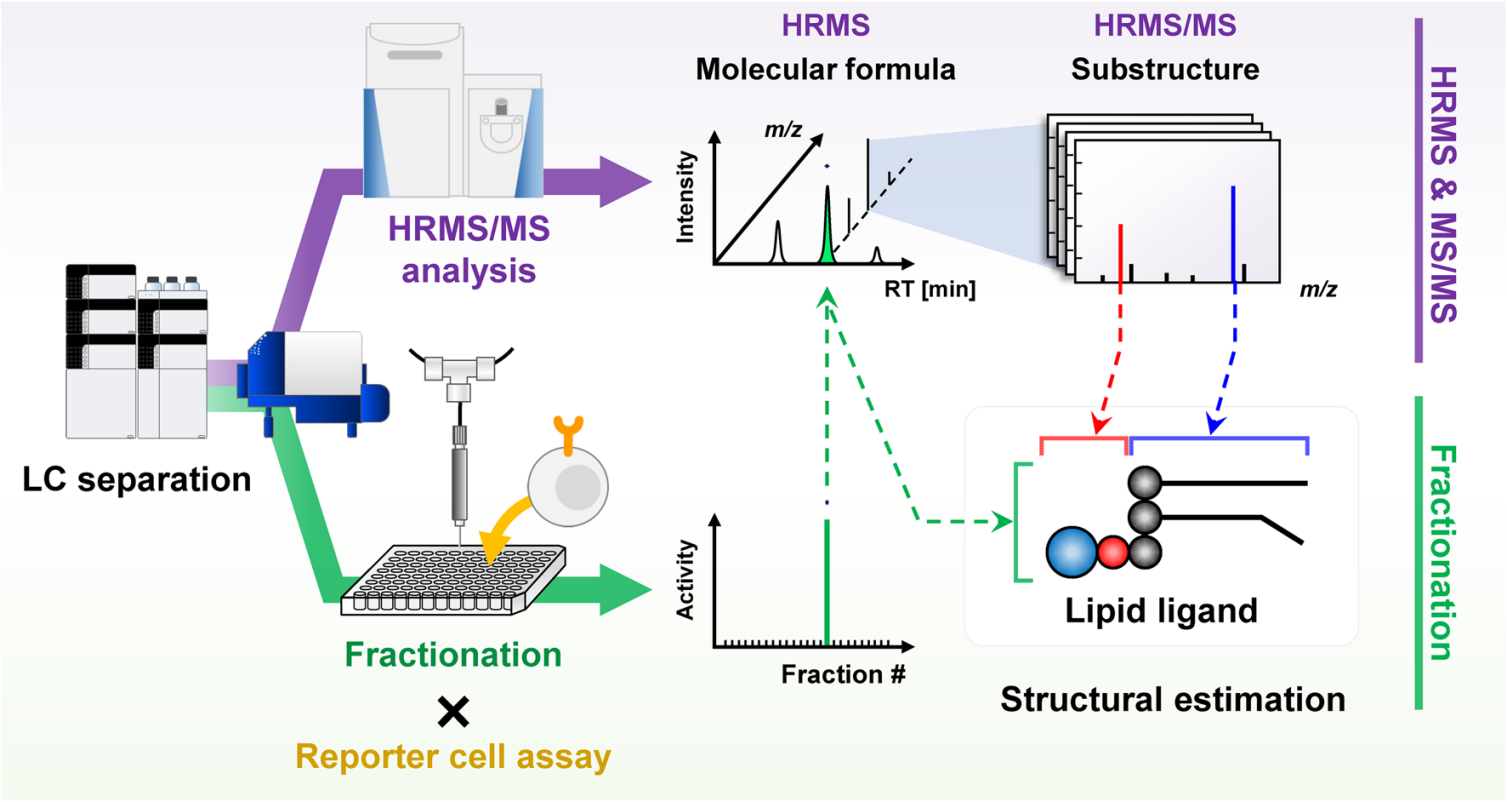
Overview and flowchart of the proposed platform for high-throughput investigation of lipid ligands

In this study, we developed a novel analytical platform that integrated fractionation, HRMS/MS analysis, and reporter cell assays based on LC-HRMS/MS with microfractionation (LC-FRC-HRMS/MS) to rapidly and accurately determine the structures of lipid ligands for immune receptors (Fig. 1). In addition, to perform structural analysis of lipids from the HRMS and HRMS/MS spectra acquired from biological samples, we constructed an in-house lipidomic library containing accurate mass and fragmentation information for more than 10,000 lipid molecular species predicted in silico for 90 lipid subclasses and 35 acyl side chain fatty acids. To verify the practicality of the developed LC-FRC-HRMS/MS system, *Helicobacter pylori*, for which specific ligands for macrophage-inducible C-type lectin (Mincle) have recently been identified [22], was used as a model sample. *H. pylori* lipid extracts were isolated and fractionated, and the HRMS and HRMS/MS spectra were acquired simultaneously. Lipid extract samples fractionated in 96-well microtiter plates were subjected to a reporter cell assay using nuclear factor of activated T cells (NFAT)-green fluorescent protein (GFP) reporter cells expressing Mincle to determine the Mincle ligands.

## Materials and methods

### Chemicals and reagents

LC–MS-grade acetonitrile, water, methanol, and isopropanol were purchased from Kanto Chemical Co. (Tokyo, Japan). LC–MS-grade ammonium acetate and RPMI-1640 medium were obtained from Merck (Darmstadt, Germany). LC–MS-grade acetic acid was purchased from Fujifilm Wako Pure Chemical Co. (Osaka, Japan). HPLC-grade chloroform was purchased from Nacalai Tesque Inc. (Kyoto, Japan). Ten percent fetal bovine serum (FBS) was purchased from Capricorn Scientific GmbH (Ebsdorfer, Germany). Lipid standards (Supplementary Table 1) were purchased from Avanti Polar Lipids, Inc. (Alabaster, AL, USA), Cayman Chemical Co. (Ann Arbor, MI, USA), and Merck.

### Bacteria

*H. pylori* strain SS1, a mouse-adapted human isolate, was used in this study. Glycerol stocks of *H. pylori* SS1 were cultured on 5% sheep blood agar plates (BBL 251239; BD Biosciences, Franklin Lakes, NJ, USA) under microaerobic conditions for 2 days. After plate culture, *H. pylori* SS1 was cultured in Brucella broth (BD Biosciences) containing 5% fetal calf serum for 14‒16 h at 37 °C under microaerobic conditions with gentle shaking. *H. pylori* SS1 was thereafter harvested by centrifugation and subjected to lipid extraction.

### *H. pylori* sample preparation

Lipids were extracted from *H. pylori* SS1 using the Bligh and Dyer method [30] with minor modifications [31, 32]. Briefly, bacterial samples (50 mg wet weight ≒ 5 mg dry weight) was mixed with 1 mL of cold methanol (− 30 °C) and vortexed for 1 min followed by sonication for 5 min. The samples were centrifuged at 16,000 × *g* and 4 °C, for 5 min. The supernatant (600 µL) was transferred to a 2-mL Eppendorf tube, mixed with 600 µL of chloroform and 480 µL of water, and vortexed for 1 min. After centrifugation for 3 min at 16,000 × *g* at 4 °C, the organic (lower) layer (450 µL) was transferred to a 1.5-mL Eppendorf tube. The organic extracts were evaporated with nitrogen gas, and the dried extracts were stored at − 80 °C until LC-FRC-HRMS and LC-FRC-HRMS/MS analyses. The samples were dissolved in 100 µL methanol/chloroform (1:1, v/v) prior to analyses.

### Flow injection conditions

Flow injection high-resolution tandem mass spectrometry (FI-HRMS/MS) was performed using lipid standards from 90 different subclasses to obtain the HRMS and HRMS/MS spectra. The FI-HRMS/MS system comprised a Nexera X2 UHPLC system (Shimadzu Co., Kyoto, Japan) and a Q Exactive high-performance benchtop quadrupole Orbitrap mass spectrometer (Thermo Fisher Scientific Inc., Waltham, MA, USA) with a heated ESI source. The LC system was equipped with two binary pumps, a temperature-controlled column component, and an autosampler. The following FI conditions were used: mobile phase (A) and (B), 10 mM ammonium acetate in methanol; injection volume, 2 μL; and flow rate, 0.2 mL/min. The full-scanning HRMS analysis conditions were as follows: polarity, positive and negative ionization; sheath gas flow rate, 40 arb for positive ionization and negative ionization; auxiliary (Aux) gas flow rate, 10 arb; spray voltage, 3.5 kV for positive ionization, − 2.5 kV for negative ionization; capillary temperature, 250 °C; heater temperature, 400 °C; mass resolution, 70,000; automatic gain control (AGC) target (the number of ions to fill the C-trap), 3,000,000; maximum injection time (IT), 200 ms; and scan range, 150‒2000 (*m/z*). The HRMS/MS conditions using parallel reaction monitoring (PRM) for each target compound were as follows: mass resolution, 70,000; AGC target, 1,000,000; maximum IT, 100 ms; isolation window, 0.4 (Da); and stepped normalized collision energy, 10, 30, and 40 eV.

### LC-HRMS conditions

LC-HRMS analysis was performed to evaluate the separation of a wide range of lipid molecular species by reversed-phase LC (RP-LC) and to determine the retention time (RT) of each lipid subclass standard. The two LC columns used to examine the separation patterns and peak shapes of lipid molecules using the 90 lipid subclass standards were as follows: InertSustain C18, 3 μm particle size, 2.1 mm inner diameter (i.d.) × 150 mm (GL Sciences Inc., Tokyo, Japan); and metal-free PEEK-coated InertSustain C18, 3 μm particle size, 2.1 mm i.d. × 150 mm (GL Sciences Inc.). The LC conditions were as follows: mobile phase (A), 5 mM ammonium acetate in water/acetonitrile (1:2, v/v); mobile phase (B), 5 mM ammonium acetate in methanol/isopropanol (1:19, v/v); injection volume, 5 μL; flow rate, 0.2 mL/min; and column temperature, 50 °C. The gradient conditions were as follows: 0‒100% B, 0‒74 min; 100% B, 74‒84 min; 100‒0%, 84‒84.1 min; and 0%, 84.1‒90 min. The AGC target for the HRMS analysis was set to 1,000,000. The other full-scanning HRMS analysis conditions were the same as those used for the FI-HRMS analysis.

### LC-FRC-HRMS/MS conditions

LC-FRC-HRMS/MS (LC-NanoMate-HRMS/MS) analysis was performed using an LC system (Nexera X2; Shimadzu Co.) coupled with a Q Exactive system (Thermo Fisher Scientific Inc.) equipped with a TriVersa NanoMate (Advion Inc., Ithaca, NY, USA). A metal-free PEEK-coated InertSustain C18 column (3 μm particle size, 2.1 mm i.d. × 150 mm, GL Sciences Inc.) was used for LC separation. The other LC conditions were the same as those used for LC-HRMS analysis. The HRMS analysis conditions were as follows: polarity, positive and negative ionization; capillary temperature, 200 °C; heater temperature, 350 °C; mass resolution, 70,000; AGC target, 3,000,000; maximum IT, 200 ms; and scan range, 150‒2000 (*m/z*). The data-dependent MS (dd-MS) conditions for each target compound were as follows: mass resolution, 17,500; AGC target, 100,000; maximum IT, 80 ms; isolation window, 0.4 (Da); inclusion mass tolerance, 10 ppm; exclusion mass tolerance, 5 ppm; and normalized collision energy, 20 eV. LC-FRC-HRMS/MS analysis used the compound features obtained from the full-scan HRMS data of the *H. pylori* sample as the inclusion list and the compounds that were significantly detected in the methanol/chloroform (1:1, v/v) blank as the exclusion list.

**Fig. 2:**
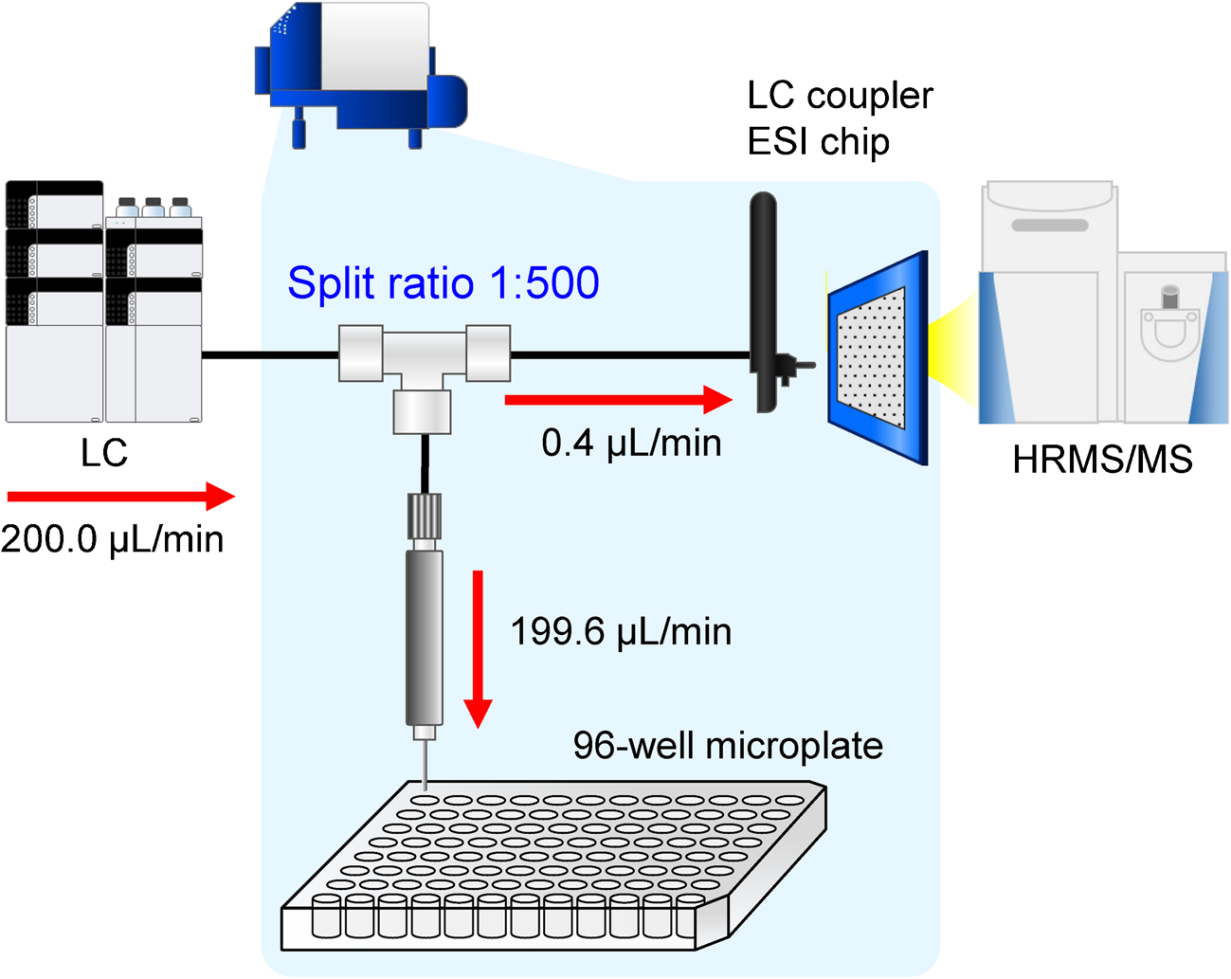
Schematic of the LC-NanoMate-HRMS/MS system for separation, fractionation, and HRMS/MS analysis of lipid components

The flow path of the LC-FRC system was designed by connecting a TriVersa LC coupler with a fused silica capillary (15 μm i.d., 360 μm outer diameter (o.d.), 29.8 mm length, Advion Inc.) and three types of fused silica capillary tubes (75 μm i.d., 360 μm o.d., 40.0 mm length; 100 μm i.d., 360 μm o.d., 20.0 mm length; and two sets of 150 μm i.d., 360 μm o.d., 75.0 mm length, GL Sciences Inc.) (Supplementary Fig. 1). The TriVersa NanoMate conditions were as follows: ESI-chip (A-chip, nozzle i.d. = 5 μm, Advion Inc.) voltage, 1.85 kV; well depth of fraction mandle, 2.0 mm; and maximum height of fraction mandle, 12.8 mm. The distance between the ESI-chip and the Q Exactive capillary tube was manually set to 2.0 mm. Spray detection was triggered when the spray current exceeded 5 to 500 nA for more than 5 s, after 30 s of analysis. The preparative was set to DigIn1 to receive the LC signal and immediately start the preparative. Eluates of the lipid fraction separated by RP-LC were collected in 96-well flat-bottom microtiter plates (Costar 3596; Corning Inc., Corning, NY, USA) manually installed on a NanoMate. Figure 2 shows a schematic of the LC-NanoMate-HRMS/MS system in the preparative mode using a 96-well microplate. Microplate preparations were performed at a rate of 1 well/min from 0 to 84 min for a total of 90-min LC analysis. After LC separation of the *H. pylori* extracts, the lipid fraction solution collected in 96-well microtiter plates was evaporated with nitrogen gas, and the dried extracts were stored at − 80 °C until reporter cell assay.

### Peak alignment and detection

The Compound Discoverer ver. 3.2 (Thermo Fisher Scientific Inc.) was used for data processing, including peak alignment, peak detection, data grouping, gap filling, and background subtraction [33]. Peak alignment for the two datasets containing LC-FRC-HRMS/MS data (procedure blank and *H. pylori* samples) was performed individually based on a nonparametric peak alignment algorithm using base peak chromatograms. A flowchart of each processing step is shown in Supplementary Fig. 2. Details of the peak alignment and detection procedures are provided in Supplementary Table 2.

### Lipid annotation

The metabolic features of *H. pylori* were generated from LC-FRC-HRMS full-scan data of *H. pylori* lipid extracts using Compound Discoverer ver. 3.2. Structural annotation of lipids from *H. pylori* was performed by comparing the HRMS (precursor ion mass error tolerance of < 3 ppm) and HRMS/MS (product ion mass error tolerance of < 5 ppm) data, which are important for substructural information, with those from an in-house lipidomic library.

### Reporter cell assay

The 2B4-NFAT-GFP reporter cells, which induce GFP expression after NFAT activation, were used for the reporter cell assay [34]. The 2B4-NFAT-GFP reporter cells expressing human Mincle (hMincle) or mouse Mincle (mMincle) were prepared as previously described [35]. Reporter cells were cultured in RPMI-1640 medium supplemented with 10% FBS. For the reporter cell assay, 100 µL of 2B4-NFAT-GFP hMincle or mMincle cell suspension (3.0 × 10^4^ cells) was added to each well of a 96-well microtiter plate in which the lipid fraction of *H. pylori* was isolated, collected, and dried. Cells were thereafter incubated for 24 h, and GFP expression was analyzed by flow cytometry (BD FACSCalibur™, BD Biosciences).

## Results and discussion

### Chromatographic separation evaluation of 90 lipid subclasses using a metal-free PEEK-coated RP-LC column

There are several analytical approaches for lipidomics. Shotgun lipidomics using direct infusion tandem mass spectrometry allows rapid analysis; however, isomer separation is difficult and the number of compounds detected is reduced by strong ionization suppression. RP-LC is one of the LC modes that utilize hydrophobic interactions. The separation mechanism of RP-LC for lipidomics is based on hydrophobic interactions between nonpolar side chains of C18 particles and hydrophobic fatty acyl chains of lipids, which are determined by the length and number of double bonds in the fatty acyl chains and the type of polar head groups. RP-LC–MS/MS allows for the efficient separation of lipid molecules with a wide range of polarities and is most commonly used for lipidomic analysis [36]. In this study, RP-LC was selected to construct an analytical platform. The columns used for LC were packed with packing materials (e.g., ODS particles) using frit filters to prevent leakage of the packing material. Pressure-resistant stainless used steel (SUS) is a commonly used material for frit filters. However, when an SUS-type column is used for the LC–MS analysis of phosphorylated compounds, adsorption of phosphate groups onto metals and metal oxides occurs on the column frit and internal column walls, resulting in the poor peak shape and reduced sensitivity [37]. Thus, in this study, we selected a PEEK-coated InertSustain C18 column (3 μm particle size, 2.1 mm i.d. × 150 mm) where the entire column shell including the frit is made of PEEK and packed with ODS, to verify its superiority in lipidomic analysis. This column is a UHPLC-compatible PEEK-coated column with a double-tube structure in which the outer PEEK tube is covered with stainless steel, making the wetted parts metal-free and providing excellent pressure resistance (pressure limit is 50 MPa for 3 μm particles).

**Fig. 3:**
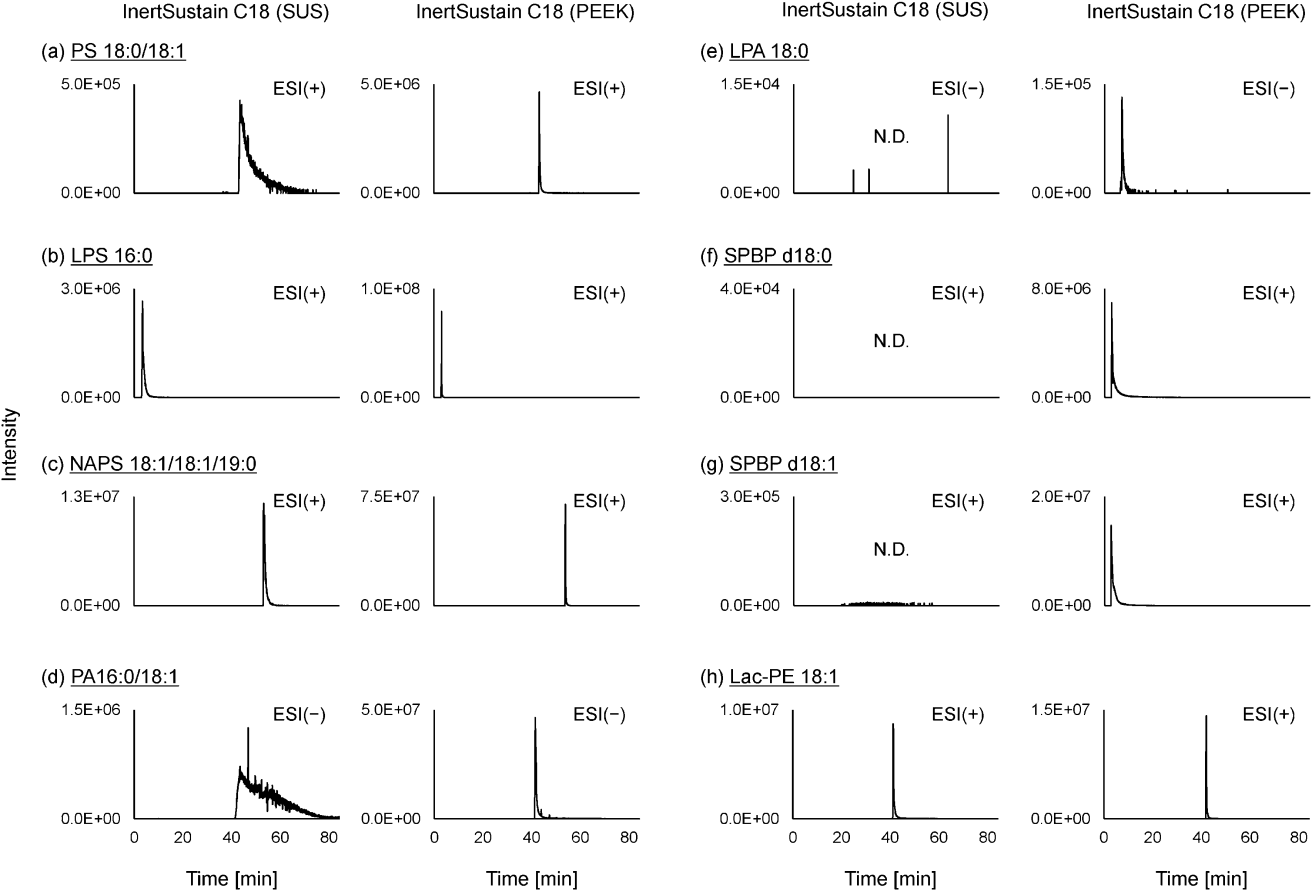
Peak shape comparison of LC-HRMS chromatograms of **a** phosphatidylserine (PS 18:0/18:1), **b** lysophosphatidylserine (LPS 16:0), **c** *N*-acylphosphatidylserine (NAPS 18:1/18:1/19:0), **d** phosphatidic acid (PA 16:0/18:1), **e** lysophosphatidic acid (LPA 18:0), **f** sphinganine-1-phosphate (SPBP d18:0), **g** sphingosine-1-phosphate (SPBP d18:1), and **h** phosphoethanolamine-*N*-lactosyl (Lac-PE 18:1) standards using InertSustain C18 (SUS) and PEEK-coated InertSustain C18 columns

The separation behavior and peak shapes of 90 lipid subclasses (Supplementary Table 1) were investigated using standard InertSustain C18 (SUS) and metal-free PEEK-coated InertSustain C18 columns. The LC-HRMS chromatogram results using the InertSustain C18 (SUS) and InertSustain C18 (PEEK) columns showed that the averaged peak width ratio (PEEK/SUS) for each lipid subclass was 0.85, indicating that the use of the metal-free PEEK-coated InertSustain C18 column improved the lipidomic peak width by an average of 15% (Supplementary Table 3). Particularly for polar lipids containing phosphate groups, such as phosphatidylserine (PS 18:0/18:1), lysophosphatidylserine (LPS 16:0), phosphatidic acid (PA 16:0/18:1), LPA (LPA 18:0), sphingosine-1-phosphate (SPBP d18:1), sphinganine-1-phosphate (SPBP d18:0), *N*-acylphosphatidylserine (NAPS 18:1/18:1/19:0), and phosphoethanolamine-*N*-lactosyl (LacPE 18:1), the use of the PEEK-coated InertSustain C18 column exhibited a significant improvement in peak shape and a trend toward increased peak area values (Fig. 3). Based on these results, we decided to use the metal-free PEEK-coated InertSustain C18 for a wide range of lipidomic analyses, including polar lipids.

### Construction of in-house lipidomic library

Several compound features were detected in the HRMS spectra obtained by analyzing the biological samples. To quickly perform structural analysis using a large number of compound features in a biological sample, it is necessary to fully understand the structural information of the lipid molecules in advance and sort them by comparison and matching. The structural information of lipid molecules can be predicted in silico using the structural information of the head groups and fatty acid side chains of each lipid subclass. In addition, lipid molecules of the same lipid subclass were characterized by a common fragmentation pattern in HRMS/MS analysis [38]. Therefore, the fragmentation pattern of each lipid subclass obtained by measuring a lipid subclass standard product using HRMS/MS can be used to annotate the fragment ions of each lipid molecule. The criteria used to annotate the structure of a compound using LC-HRMS/MS were the HRMS spectrum acquired by HRMS with high mass accuracy, HRMS/MS spectrum acquired by HRMS/MS with high mass accuracy, and the RT of a standard [39, 40].

In the present study, we constructed an in-house lipidomic library for rapid structural annotation of bioactive lipids based on previous studies of structure-specific fragment ions in tandem mass spectra of various lipid subclasses (double bond position in acyl side chains and *cis*/*trans* configuration are not considered) [41–44]. We targeted 90 lipid subclasses (Supplementary Table 1) with 35 fatty acid side chains (Supplementary Table 4); thus, the total number of lipid species exceeded 10,000. First, 90 standards of the lipid subclasses were measured by FI-HRMS/MS in positive and negative modes, and the HRMS/MS spectra were recorded (Supplementary Fig. 3). To interpret the HRMS/MS spectra, the minimal criteria for the annotation of fragmentation patterns were applied (Supplementary Table 5). The selection of specific fragment ions of each lipid subclass, independent of the polarity of the positive and negative modes, allowed the identification of the analogs. For instance, in the case of lysophosphatidylcholine (LPC), the adduct of the precursor ion in the positive ion mode is [M + H]^+^, and the product ion is [C_5_H_14_NO_4_P + H]^+^. Similarly, the adduct ion of the precursor ion in the negative ion mode was [M + CH_3_COO]^−^, and the product ions was [Acyl FA − H]^−^. However, when product ions or neutral losses from the acyl side chains could not be identified, only the lipid subclass was annotated from the head group product ions. To perform the structural annotation of lipids, accurate masses and in silico predicted significant fragment ions for more than 10,000 lipid molecular species combining 90 lipid subclasses and 35 fatty acids were registered in an in-house lipidomic library. The RTs for 90 lipid subclass standards measured by RP-LC-HRMS using a metal-free PEEK-coated InertSustain C18 column were also entered into the in-house lipidomic library and used as reference values for lipid annotation (Supplementary Table 3).

### Development of LC-FRC-HRMS/MS system

In this study, we constructed an analytical platform that enables the rapid separation, fractionation, and identification (annotation) of lipid molecules simultaneously from biological samples as small as 1‒10 mg dry weight. To this end, an LC-FRC-HRMS/MS analytical system was constructed with Nexera UHPLC, TriVersa NanoMate, and Q Exactive. A schematic of the LC-NanoMate/HRMS/MS analytical system is shown in Fig. 2. As shown in Fig. 2, the mobile phase pumped from the LC at a flow rate of 200.0 µL/min splits in the TriVersa NanoMate at a separation ratio of 1:500. Therefore, theoretically, 400 nL/min is delivered to Q Exactive and 199.6 µL/min is delivered to the 96-well microplate for the reporter cell assay. In general, ESI–MS is a concentration-dependent detector [45]. NanoMate is equipped with a nano-ESI ion source and enables ionization even at a flow rate of 400 nL/min. Therefore, HRMS and HRMS/MS spectra can be obtained even when a large portion of the sample is collected in a microplate for the reporter cell assays. The separated compounds were collected in a 96-well microplate at short time intervals, and the fractions were subdivided to increase the throughput of ligand identification.

The fused silica capillary tubes used for the flow paths were set to the theoretically calculated inner diameters and lengths to allow simultaneous fractionation and analysis (Supplementary Fig. 1). The split ratio of the LC-NanoMate-HRMS/MS system was 1:500, and the flow rate to the LC coupler side was extremely low (400 nL/min). Therefore, the inner diameter and length of the capillary tube were optimized for more stable pumping, and the back pressure was adjusted such that sufficient mobile phase could be pumped to the LC coupler side. To verify that the flow rate of the LC coupler matched the calculated theoretical value, distilled water was sampled as follows: the flow rate of the LC was set to 200 µL/min and distilled water was used as the mobile phase. The flow rate was calculated by collecting distilled water in a glass tube for 30 min and measuring the weight and density of the water at a given temperature on the day of the experiment. The length of the capillary tube (i.d. 75 µm) used for the flow path from the T-piece to the four-way valve (Supplementary Fig. 1) was thereafter varied to 30, 40, 50, 60, and 70 cm, and the flow rate was measured at each flow path length. The results demonstrated that the flow rate from the LC coupler side was 350 nL/min at the default channel length of 40 cm (Supplementary Fig. 4). To eliminate the discrepancy between fractionation and HRMS/MS analysis, a 60 cm capillary tube with a flow rate of 400 nL/min was used.

Next, lipid subclass standards were selected at each RT (20, 40, 50, and 60 min) under RP-LC analytical conditions using metal-free PEEK-coated InertSustain C18 and measured to confirm the time lag between fractionation and HRMS/MS analysis at each time point. The lipid subclass standards used to verify the time lag were as follows: ether monoacylglycerol (ether MG O-16:0, RT = 20 min); phosphatidylcholine (PC 15:0/16:0, RT = 40 min); β-galactosyl ceramide (β-GalCer d18:1/24:1, RT = 50 min); and acylceramide (ACer d18:1/17:0, RT = 60 min) (Supplementary Fig. 5a). The mixed solutions were fractionated and analyzed every 30 s using an LC-NanoMate-HRMS/MS system. The fraction samples were analyzed using FI-HRMS to confirm that the acquired HRMS spectra and the fractions were consistent. For instance, a compound identified at a RT of 20.25 min according to the acquired HRMS spectrum is expected to be fractionated at 20‒20.50 min. Supplementary Fig. 5b shows the average peak areas obtained by analyzing the fractionated samples by FI-HRMS. These results indicate that the lipid subclass standards were fractionated simultaneously with the measurement time on the HRMS side.

### Analysis and annotation of *H. pylori* metabolites

*H. pylori* is a gram-negative pathogenic bacterium that causes chronic gastritis. *H. pylori* infection is a major risk factor for gastric malignancy. In contrast, CLRs, which are pattern recognition receptors, recognize pathogen-specific lipid molecules. Recent studies have identified α-cholesteryl glucoside (AHexCS), a *H. pylori* lipid molecule, as a specific ligand for Mincle [22]. Therefore, in this study, using *H. pylori* as a model sample, we validated the utility of an analytical system combining LC-FRC-HRMS/MS and reporter cell assay according to the workflow described in Fig. 1.

**Fig. 4:**
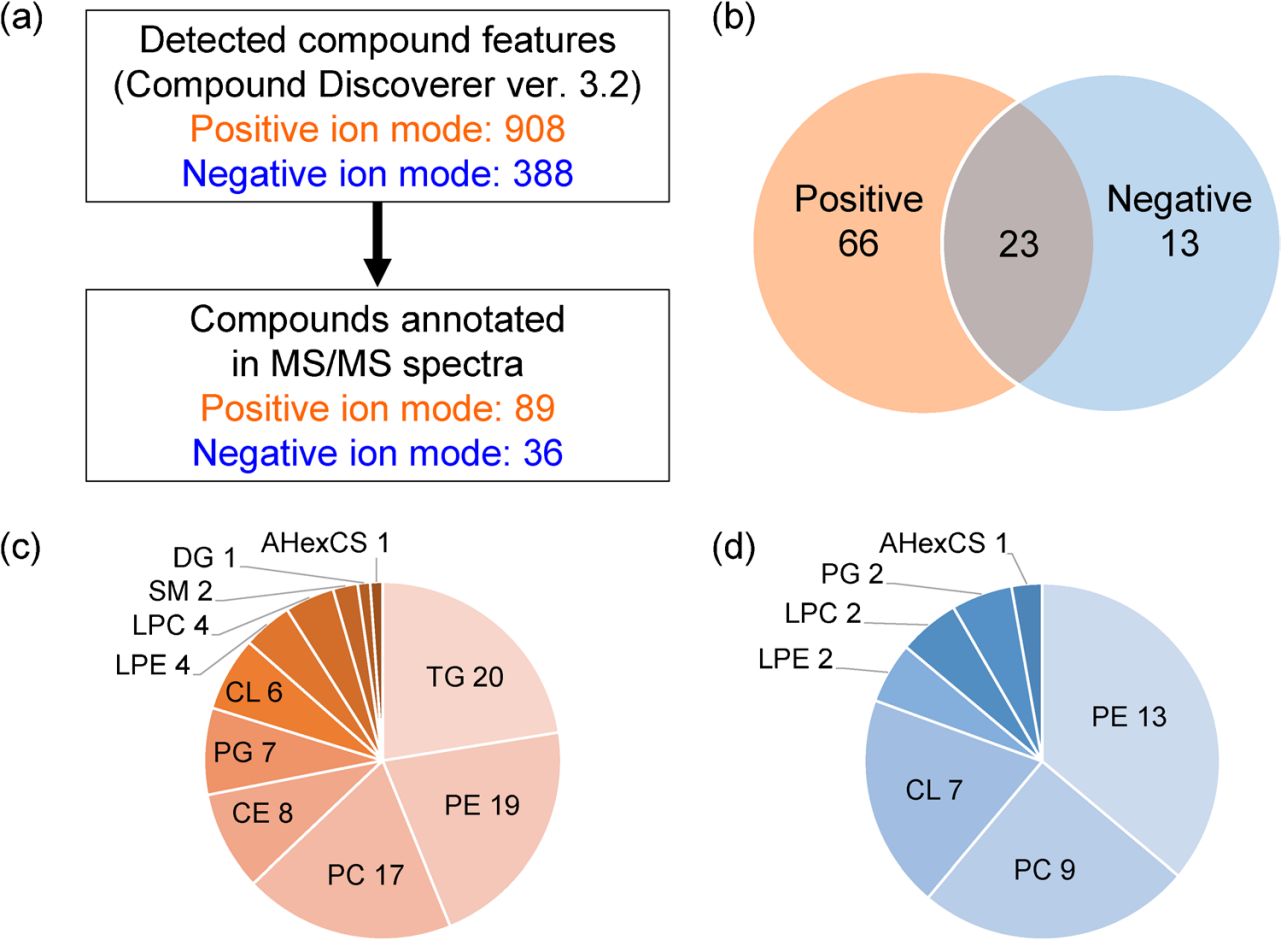
Lipid species annotated from *H. pylori* extracts. **a** Number of metabolic features detected by Compound Discoverer ver. 3.2 and annotated by HRMS/MS spectra. **b** Number of compounds annotated by HRMS/MS spectra in positive and negative ion modes, respectively. **c** Number of each lipid subclass detected in the positive ion mode. **d** Number of each lipid subclass detected in the negative ion mode. DG, diacylglycerol; TG, triacylglycerol; PC, phosphatidylcholine; LPC, lysophosphatidylcholine; PE, phosphatidylethanolamine; LPE, lysophosphatidylethanolamine; PG, phosphatidylglycerol; BPA, bisdiacylglycerophosphate; PA, phosphatidic acid; CL, cardiolipin; SM, sphingomyelin; CE, cholesterol ester; AHexCS, α-cholesteryl glucoside

First, metabolic features were detected from the LC-FRC-HRMS data of the *H. pylori* lipid extracts using Compound Discoverer 3.2 to generate an inclusion list (Supplementary Table 2). Similarly, compound features were detected from the LC-FRC-HRMS data of the solvent (methanol/chloroform (1:1, v/v)) of the *H. pylori* lipid extract to generate an exclusion list. Using the prepared inclusion and exclusion lists, dd-MS analysis was performed on the LC-NanoMate/HRMS/MS system in positive and negative ion modes, respectively, and the *H. pylori* lipid extract was fractionated in 96-well microplates. Metabolic features were thereafter detected in the acquired HRMS spectrum using Compound Discoverer 3.2. Consequently, 908 metabolic features were detected in positive ion mode and 388 in negative ion mode. These metabolic features were compared with the exact masses of lipid molecules in an in-house lipidomic library within a mass tolerance of < 3 ppm. After sorting, candidate features were annotated using the acquired HRMS/MS spectra. A total of 102 lipid molecules (89 in positive ion mode and 36 in negative ion mode) were annotated (Fig. 4a and b). The lipid subclasses detected in each ion mode are shown in Fig. 4c and d. Phosphatidylethanolamine (PE), which was the most abundant lipid molecule detected, has been reported to constitute the majority of total lipids in *H. pylor*i [46]. Several cardiolipins (CLs) were also detected, and CLs have been reported to be a type of lipid that constitutes the flagellum of *H. pylori* [47]. The results of this annotation are considered reasonable in light of previous reports annotating 67 lipid molecules from extracted samples of *H. pylori* [48].

**Fig. 5:**
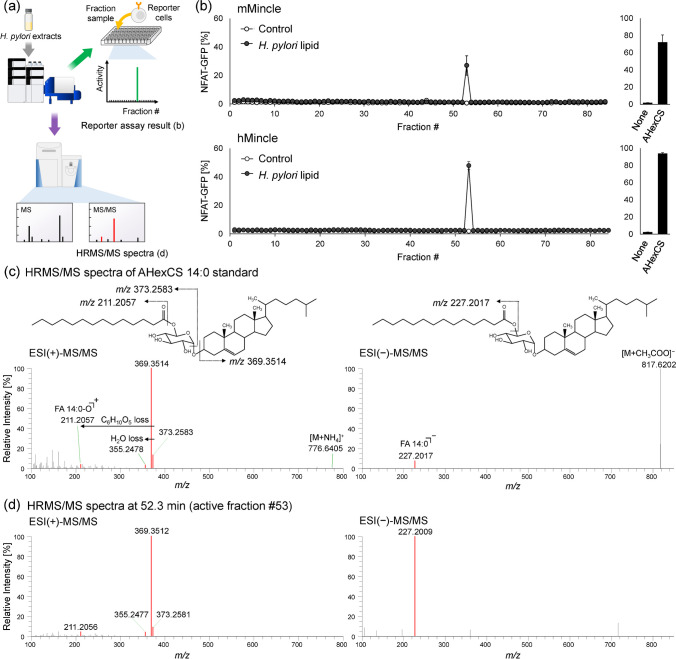
Results of reporter assay with Mincle reporter cells in the fractionated samples and HRMS/MS spectra of α-cholesteryl glucoside (AHexCS) standard and present in the active fraction. **a** Flowchart of the experiment and overview of the acquired data. **b** Results of reporter assay with 2B4-NFAT-GFP reporter cells expressing mMincle (upper) or hMincle (lower) in the fractionated samples of *H. pylori* extracts and AHexCS standard (10 µg/well). Values are mean ± standard deviation (*n* = 3). **c** Fragmentation pattern and HRMS/MS spectra of the AHexCS 14:0 standard. **d** HRMS/MS spectra of AHexCS 14:0 in the active fraction #53 (RT = 52.3 min). Specific product ions derived from AHexCS are shown in red (**c** and **d**)

Reporter cell assays were performed on fractionated and dried 96-well microplate samples of *H. pylori* lipid extracts, using 2B4-NFAT-GFP reporter cells expressing hMincle or mMincle (Fig. 5a). As a control, the procedure blank was subjected to the same process of fractionating *H. pylori* lipid extracts on 96-well microplates and performed in the same manner. Reporter cell assay results showed significant activity of fraction 53 in both hMincle and mMincle with high repeatability. Because fraction 53 corresponded to an analysis time of 52‒53 min, the compounds annotated at this time were examined, and the results demonstrated that the only compound with a consistent RT was AHexCS. The HRMS/MS spectra were thereafter annotated using the structural information available in the in-house lipidomic library, and the spectral pattern was consistent with that of the standard, confirming that the compound was AHexCS 14:0 (Fig. 5b and c). Finally, a reporter cell assay was performed using the AHexCS standard, which confirmed that the assay exhibits significant activity (Fig. 5a). Recent studies have shown that *H. pylori* uses host cholesterol to synthesize AHexCS, which is recognized by the host Mincle and triggers an inflammatory response leading to gastritis [22]. The proposed method demonstrated the ability to rapidly identify lipid ligands of immune receptors from trace samples.

## Conclusions

In the present study, an advanced platform was established for the detection of lipid ligands using the LC-FRC/HRMS/MS system, reporter cell assays for fractionated samples, structural information from in an in-house lipidomic library, and interpretation of molecular substructures based on HRMS/MS spectra. Using the developed analytical system, we identified AHexCS, a lipid ligand for Mincle, from a lipid extract of *H. pylori* with crude cell extracts of approximately 5 mg dry cell/mL.

The developed in-house lipidomic library is limited to the types of lipid subclasses stored in it. Therefore, the expansion of lipid subclasses is expected to improve the throughput of structural analysis through initial screening by comparison and matching with the predicted structures in silico. Another important issue is the development of a method to estimate the structure of unknown lipid peaks from HRMS/MS spectra. The reporter cell assay is not limited to CLRs as it can be applied to various receptors, such as GPCRs. In future, this analytical platform could be used as an effective tool for high-throughput and comprehensive lipid ligand discovery and functional evaluation of novel metabolites in combination with other biological assays.

## Supplementary Information

Below is the link to the electronic supplementary material.

Supplementary file1 (PDF 6828 KB)

Supplementary file2 (DOCX 171 KB)
