# Supplementary material for: Efficient lipidomic approach for the discovery of lipid ligands for immune receptors by combining LC-HRMS/MS analysis with fractionation and reporter cell assay
Source: Anal Bioanal Chem. 2023 Dec 23;416(25):5445–56. doi: 10.1007/s00216-023-05111-w (PMC11427514; doi:10.1007/s00216-023-05111-w)
Supplement: Supplementary file 1 — Supplementary file1 (PDF 6828 KB) [file 216_2023_5111_MOESM1_ESM.pdf]

Supplementary information

Efficient lipidomic approach for the discovery of lipid ligands for immune receptors by combining LC-HRMS/MS analysis with fractionation and reporter cell assay

Noriyuki Tomiyasu[1] · Masatomo Takahashi[1,2] · Kenji Toyonaga[3,4] · Sho Yamasaki[3,5] · Takeshi Bamba[1,2] · Yoshihiro Izumi[1,2]

[1] Department of Systems Life Sciences, Graduate School of Systems Life Sciences, Kyushu University, Fukuoka, Japan

[2] Division of Metabolomics/Mass Spectrometry Center, Medical Research Center for High Depth Omics, Medical Institute of Bioregulation, Kyushu University, Fukuoka, Japan

[3] Department of Molecular Immunology, Research Institute for Microbial Diseases, Osaka University, Osaka, Japan

[4] Section of Infection Biology, Department of Functional Bioscience, Fukuoka Dental College, Fukuoka, Japan

[5] Laboratory of Molecular Immunology, Immunology Frontier Research Center (IFReC), Osaka University, Osaka, Japan

Corresponding author
Yoshihiro Izumi
izumi@bioreg.kyushu-u.ac.jp

Contents

Supplementary Fig. 1 Optimization of the flow path design of the LC-FRC system for simultaneous fractionation and HRMS/MS analysis.

Supplementary Fig. 2 Flow chart of the data processing with Compound Discoverer 3.2.

Supplementary Fig. 3 HRMS/MS spectra and fragmentation patterns of 90 lipid subclass standards obtained by FI-HRMS/MS.

Supplementary Fig. 4 Relationship between the length of capillary tubing used in the flow path from the T-piece to the four-way valve and the actual flow velocity (mean ± standard deviation, n = 3).

Supplementary Fig. 5 RT and fractionation patterns of each lipid subclass standard. (a) LC-FRC-HRMS chromatogram of each lipid subclass standard. Polarity and RT were described in the chromatogram. (b) Peak area values of the fractionated samples obtained by FI-HRMS analysis (mean ± standard deviation, n = 3).

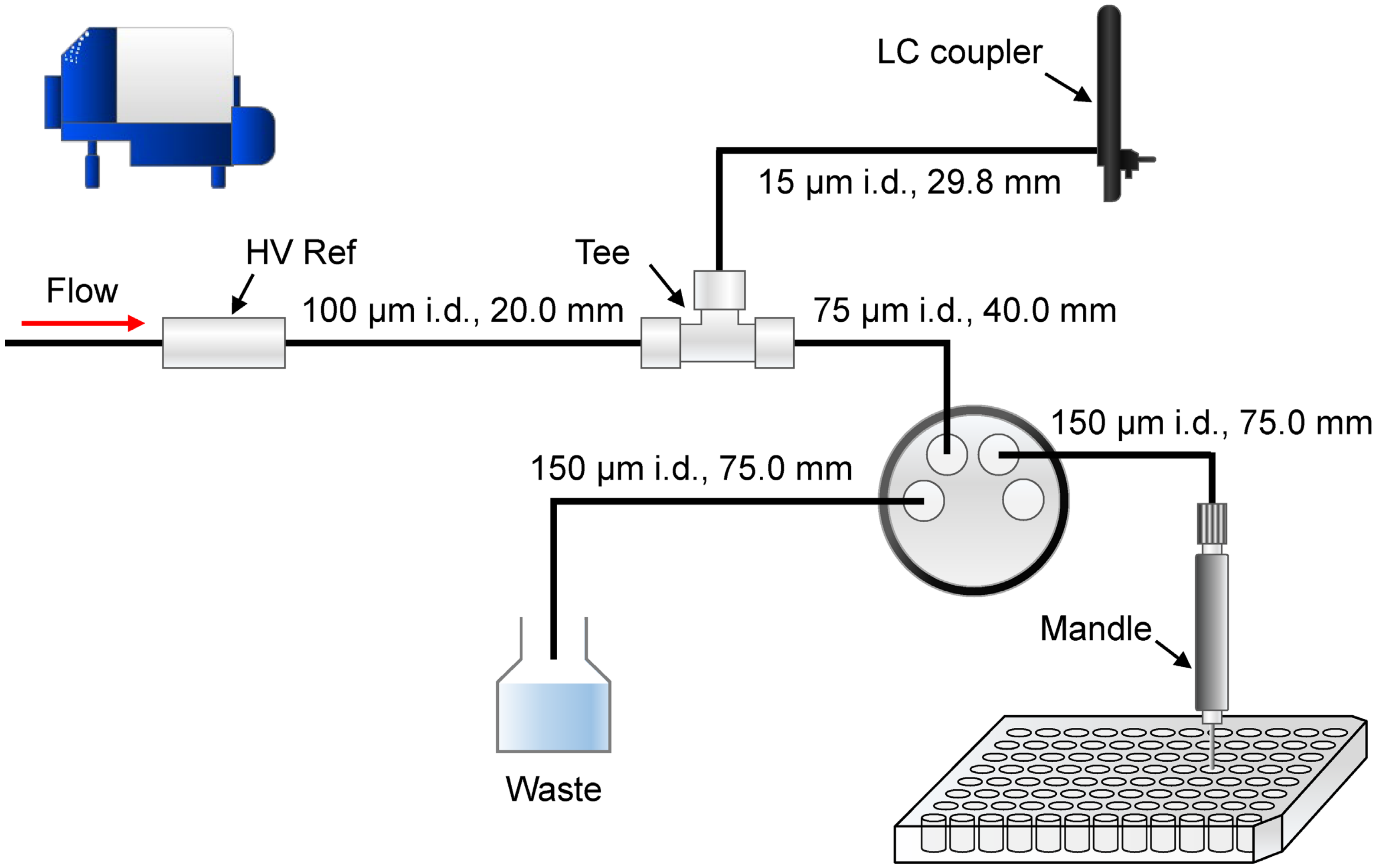

Supplementary Fig. 1 Optimization of the flow path design of the LC-FRC system for simultaneous fractionation and HRMS/MS analysis

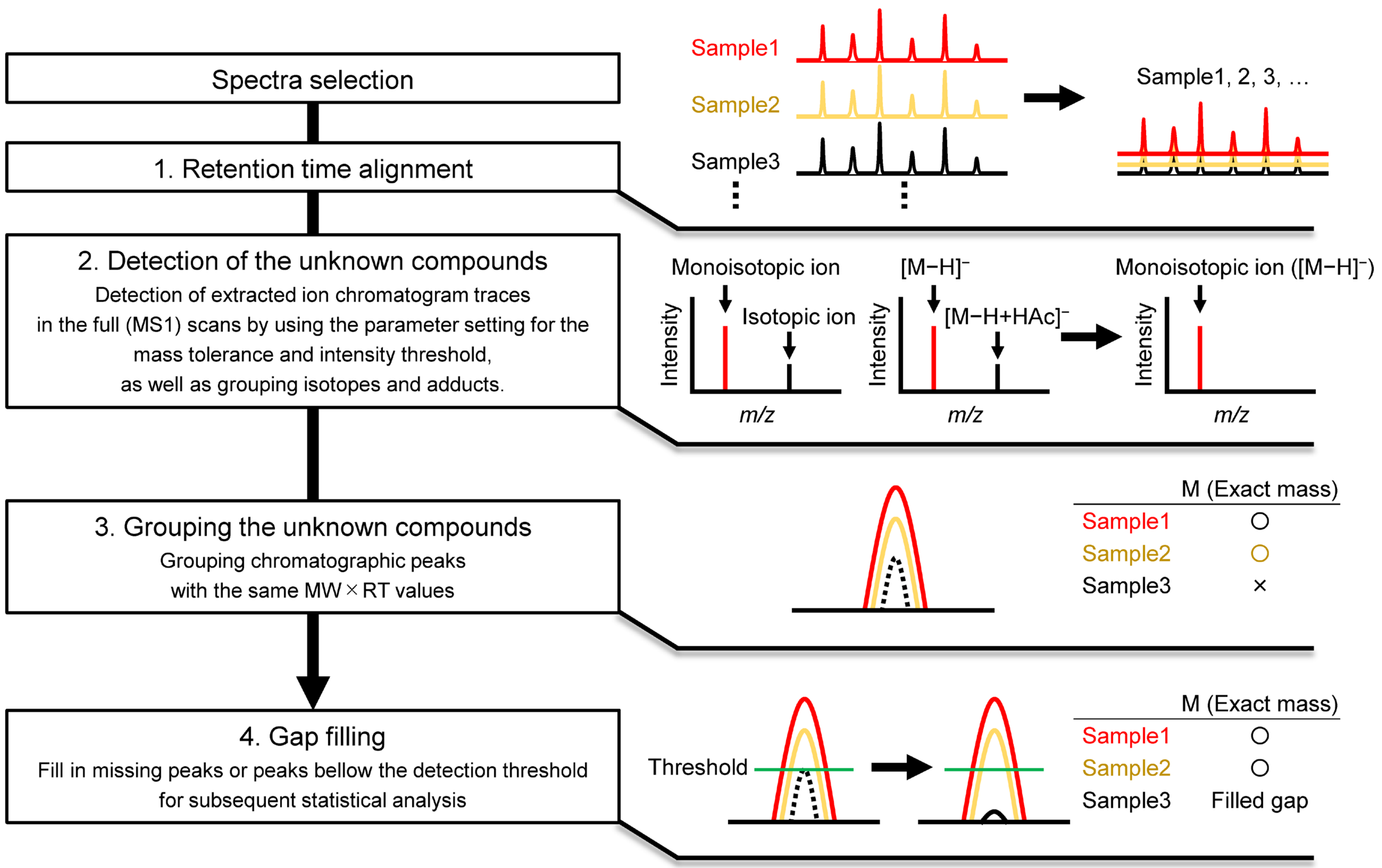

Supplementary Fig. 2 Flow chart of the data processing with Compound Discoverer 3.2.

aFA 15:0

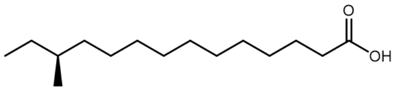

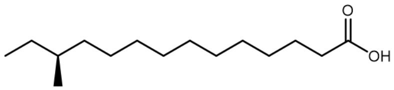

ESI(+)-MS/MS

N.D.

ESI(–)-MS/MS

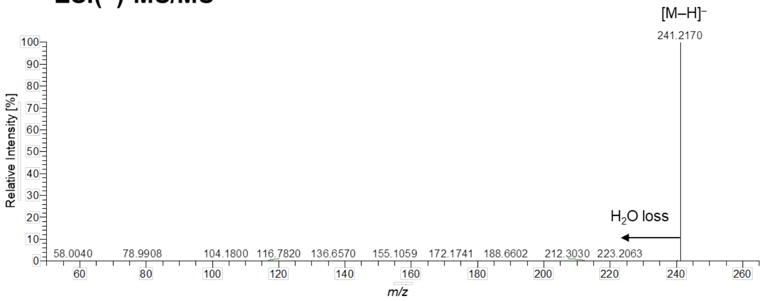

FA 32:6

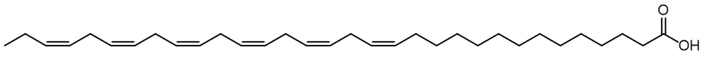

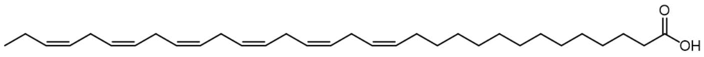

ESI(+)-MS/MS

N.D.

ESI(–)-MS/MS

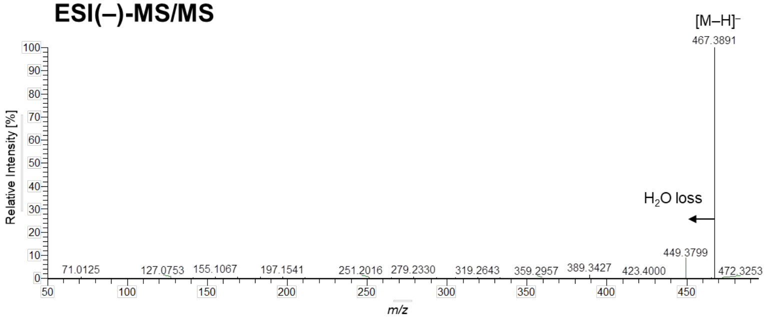

Supplementary Fig. 3 HRMS/MS spectra and fragmentation patterns of 90 lipid subclass standards obtained by FI-HRMS/MS.

Continued

HFA 18:1

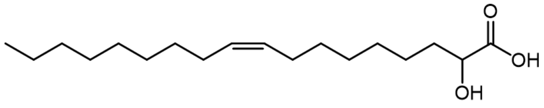

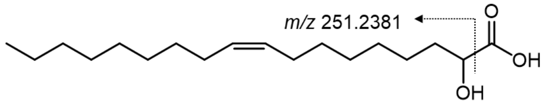

ESI(+)-MS/MS

N.D.

ESI(–)-MS/MS

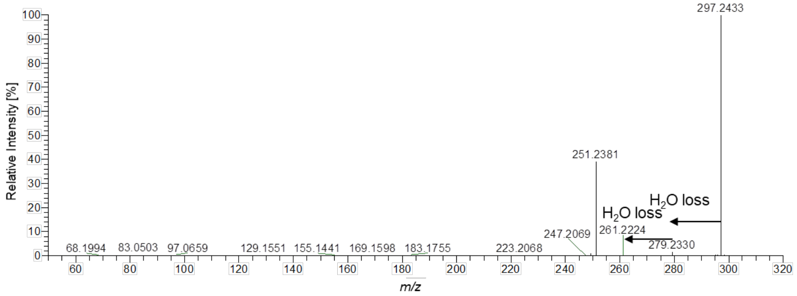

α-MA (FA80:2;O)

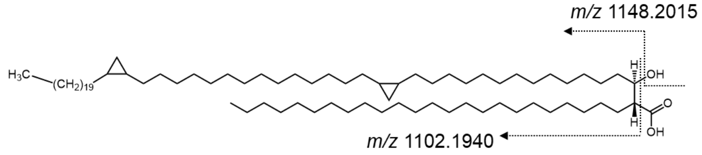

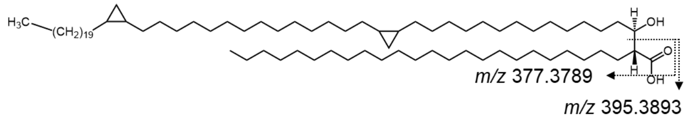

ESI(+)-MS/MS

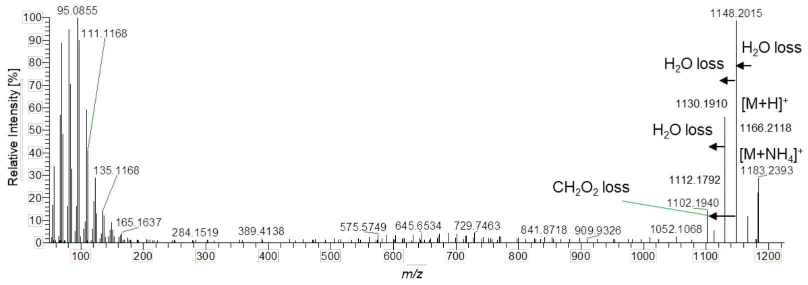

ESI(–)-MS/MS

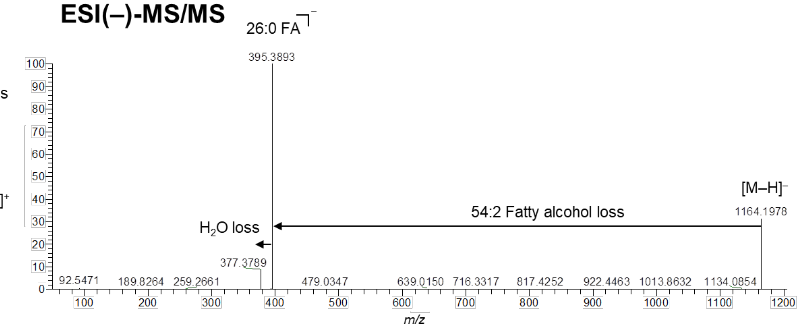

keto-MA (FA 86:2;O2)

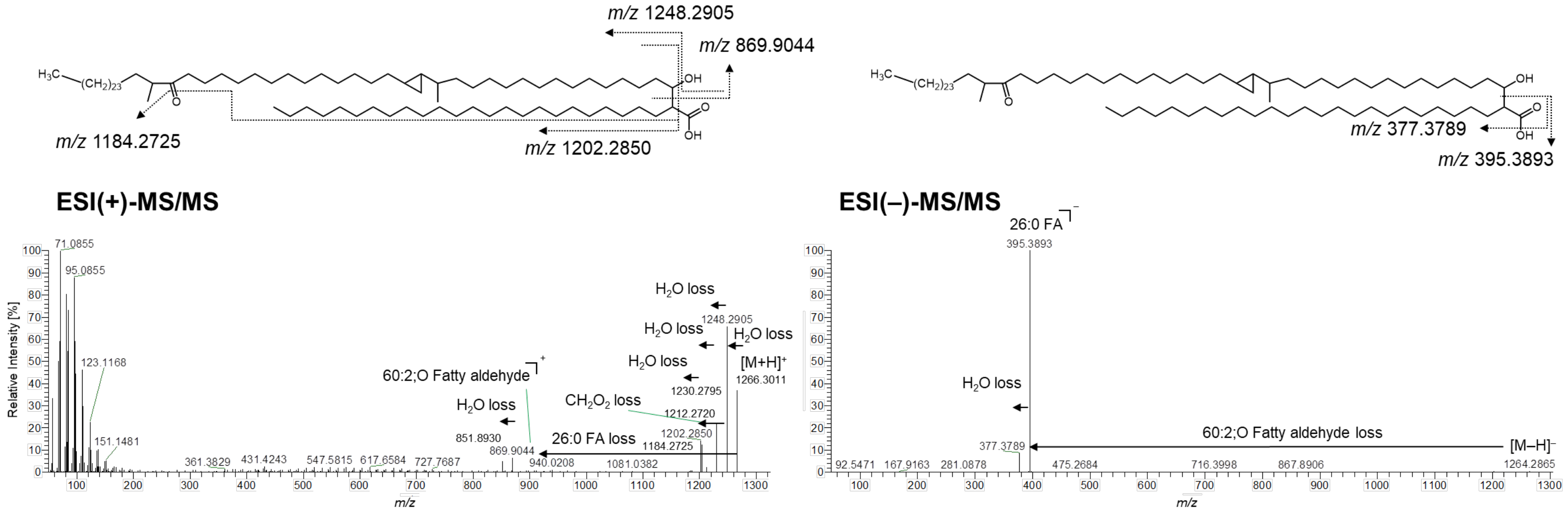

Methoxy-MA (FA 85:1;O2)

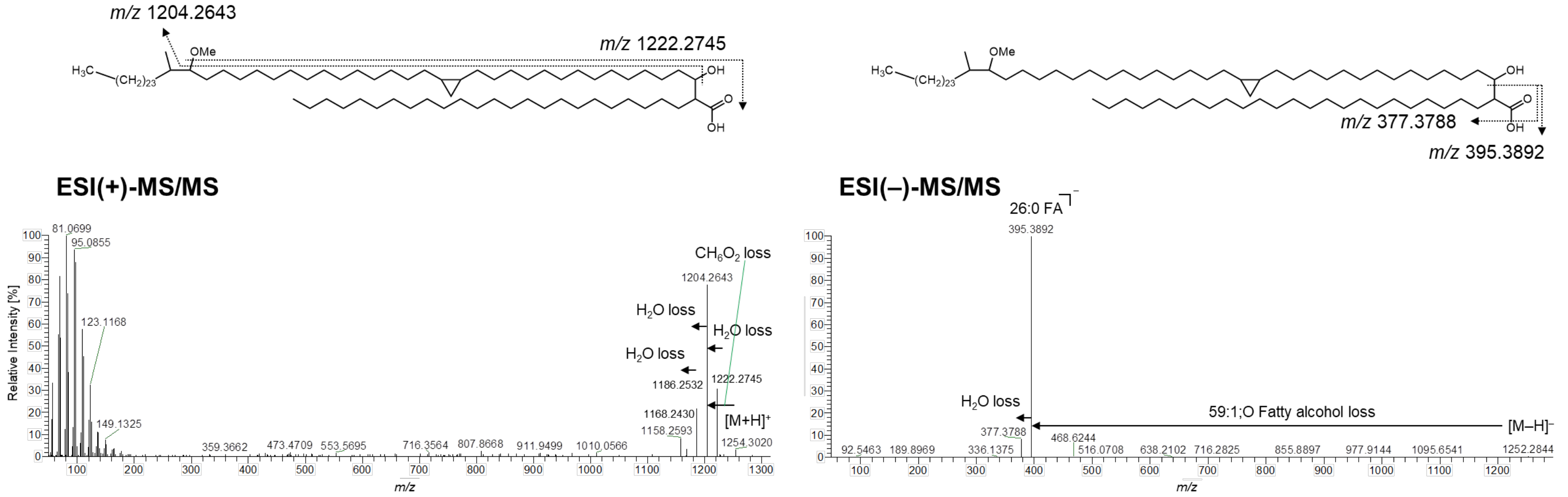

[*d*9] Fatty aldehyde 16:0

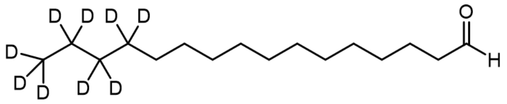

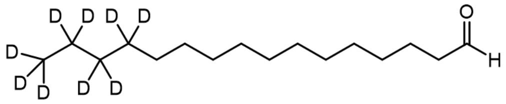

ESI(+)-MS/MS

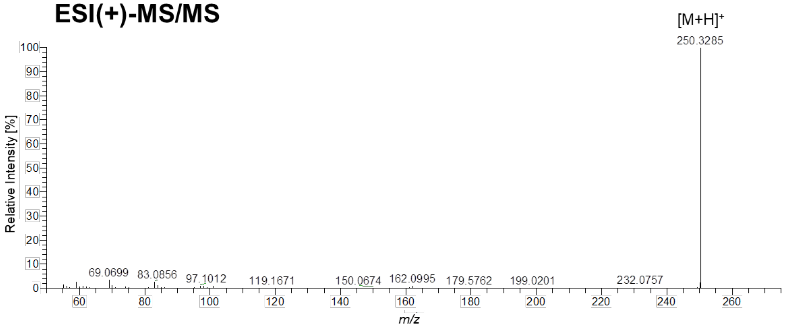

ESI(–)-MS/MS

N.D.

CAR 16:0

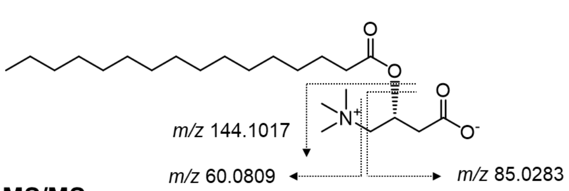

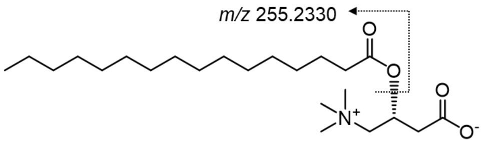

ESI(+)-MS/MS

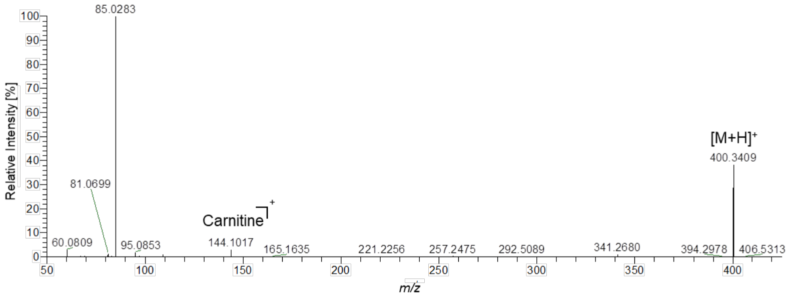

ESI(–)-MS/MS

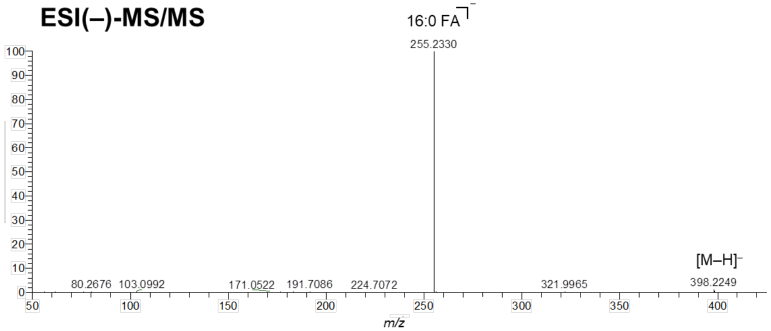

FAHFA 16:0/9-O-18:0

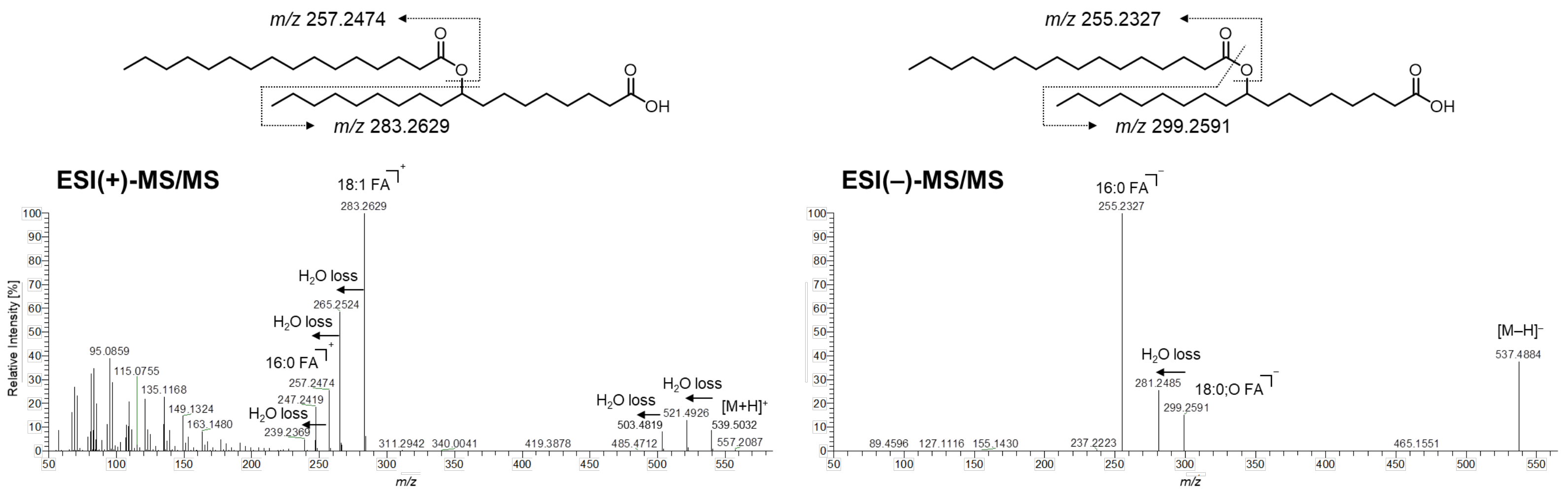

NAGly 18:1

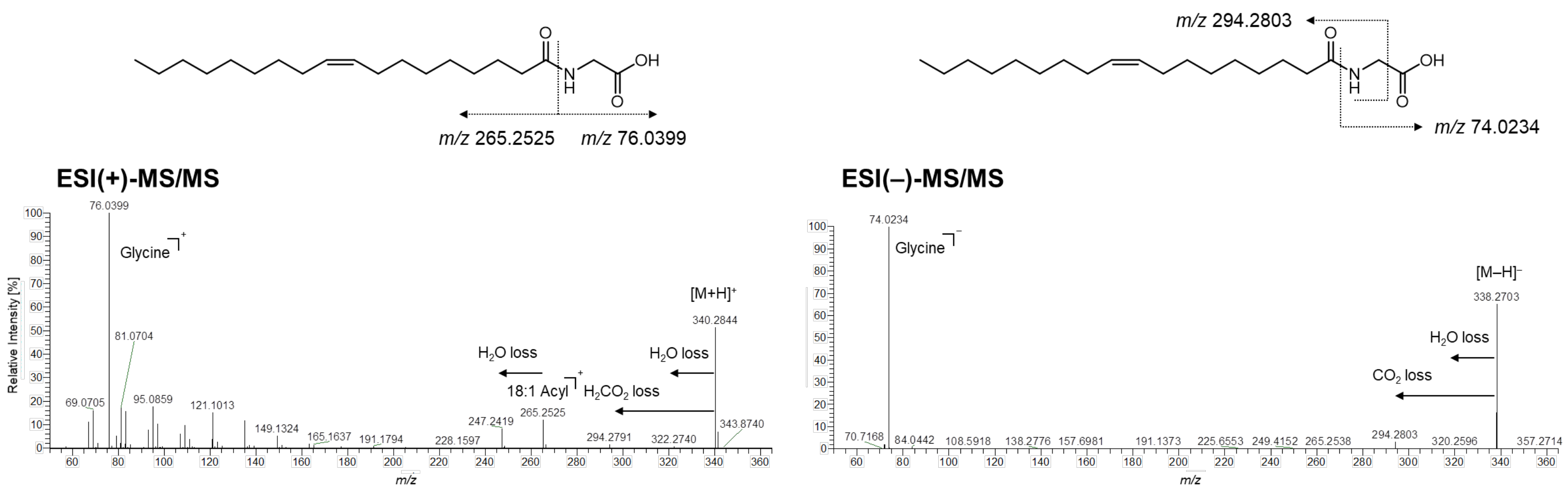

NAHC 16:0

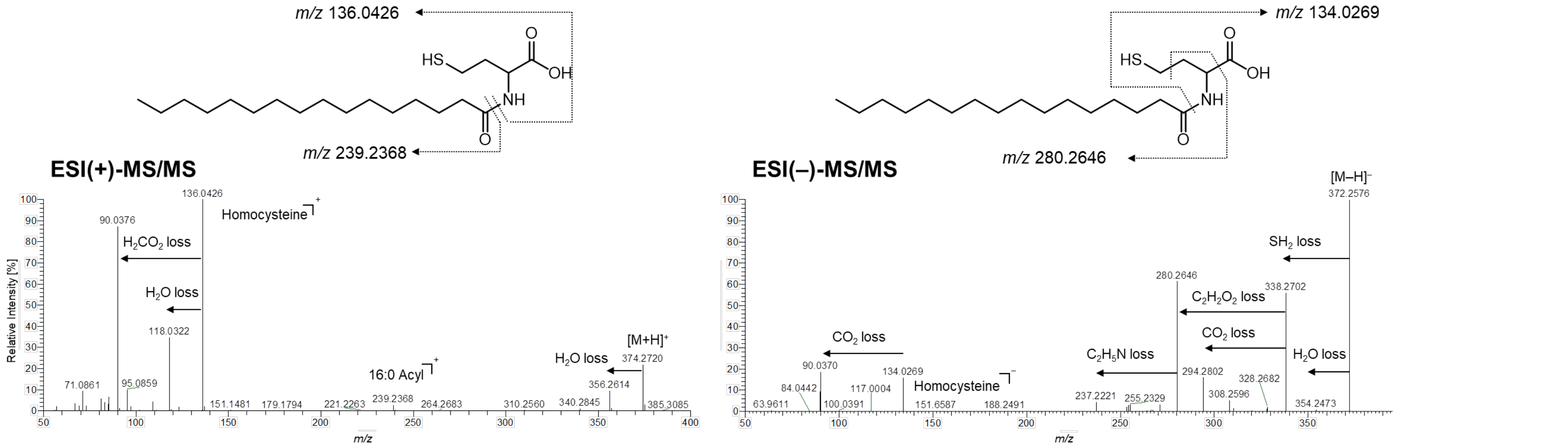

NATau 16:0

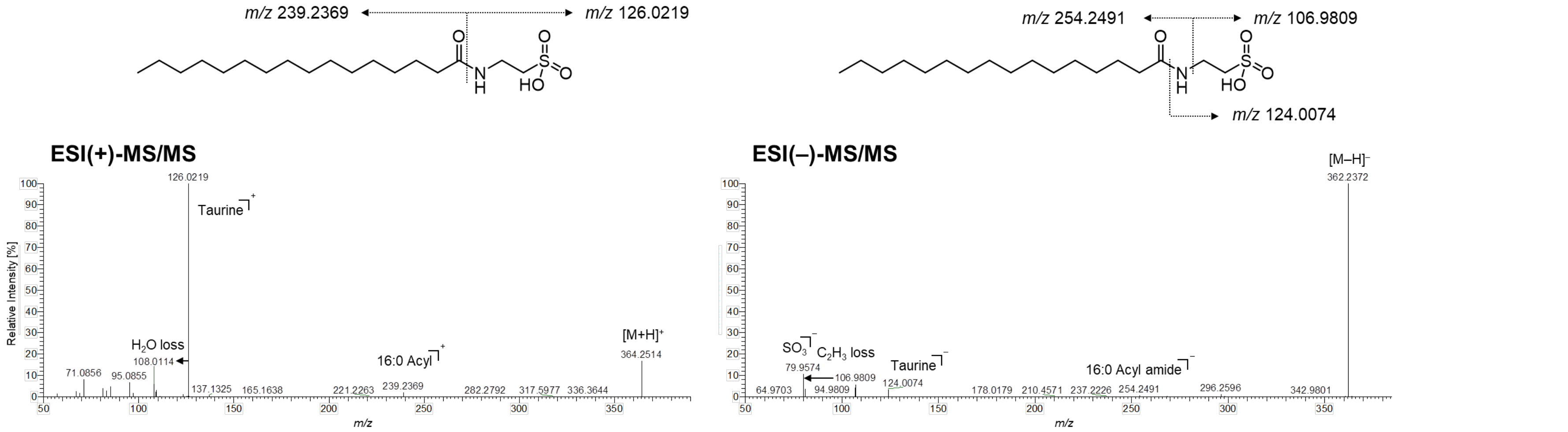

MG 16:0

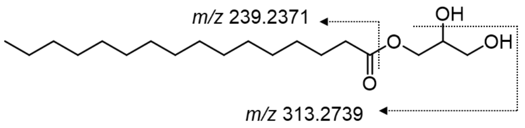

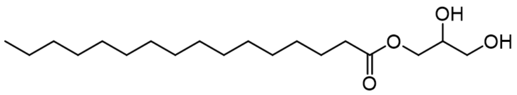

ESI(+)-MS/MS

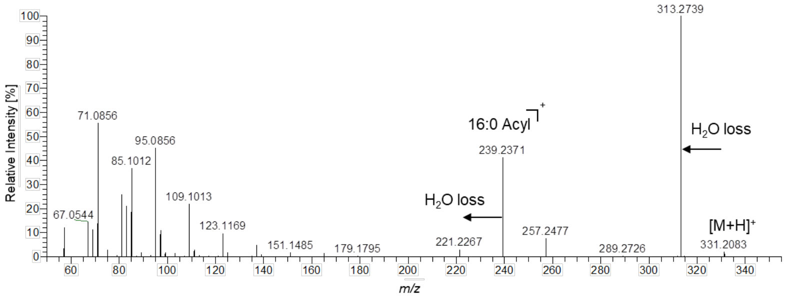

ESI(–)-MS/MS

MG O-16:0

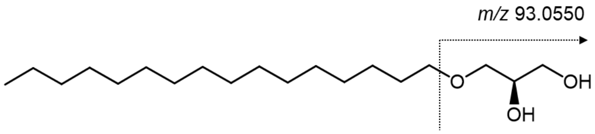

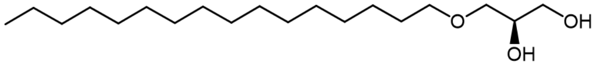

ESI(+)-MS/MS

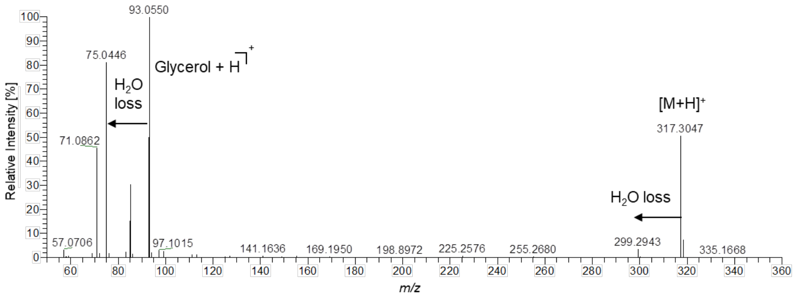

ESI(–)-MS/MS

MG P-18:0

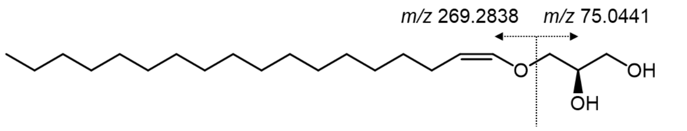

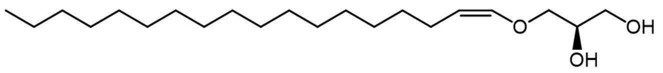

ESI(+)-MS/MS

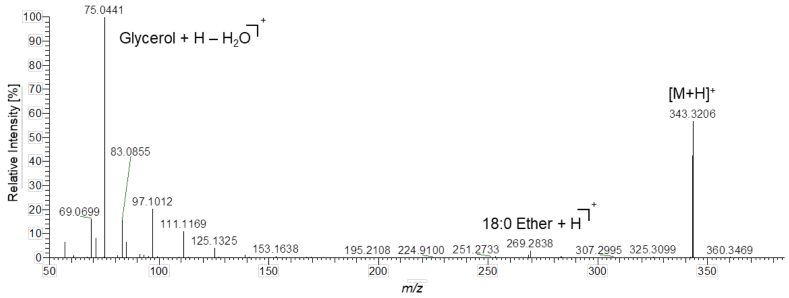

ESI(–)-MS/MS

DG 18:0/20:4

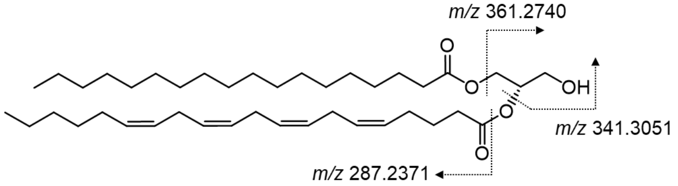

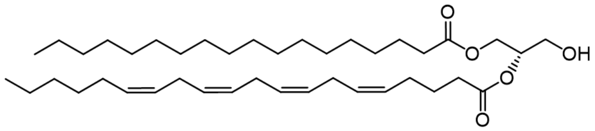

ESI(+)-MS/MS

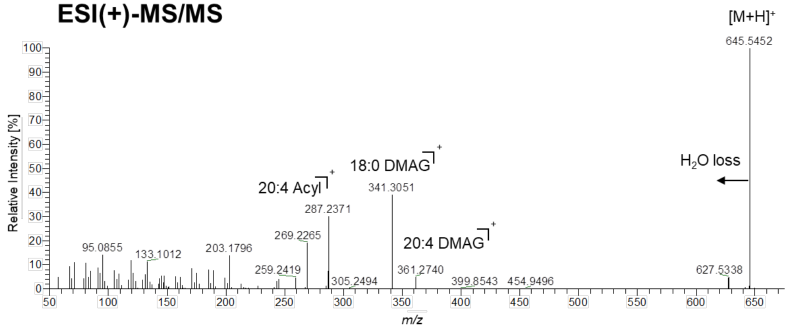

ESI(–)-MS/MS

TG 18:0/18:0/18:0

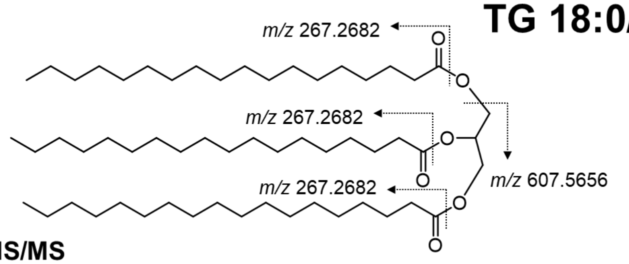

ESI(+)-MS/MS

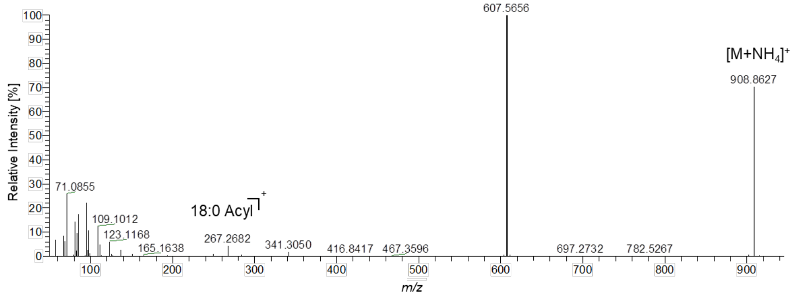

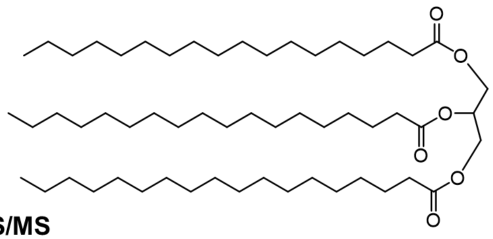

ESI(–)-MS/MS

N.D.

PC 16:0/18:1

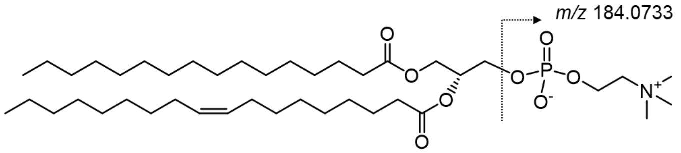

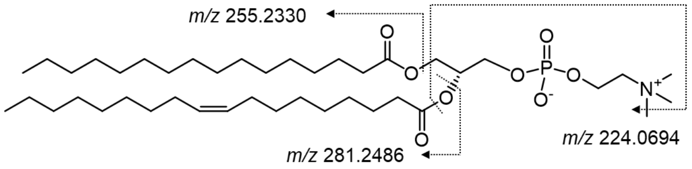

ESI(+)-MS/MS

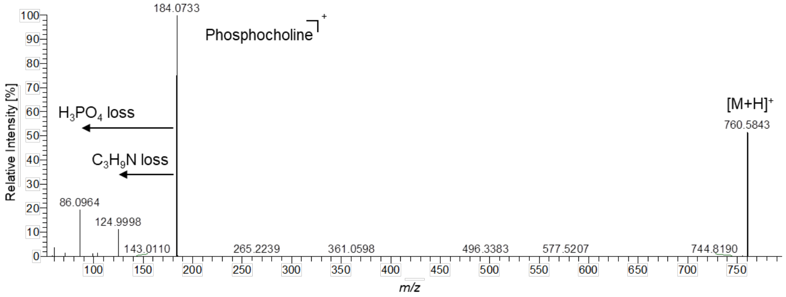

ESI(–)-MS/MS

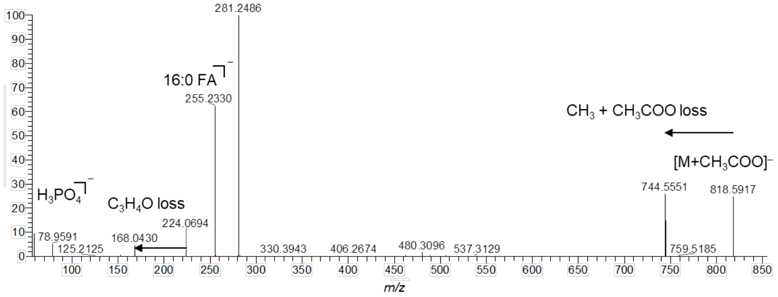

PC O-16:0/20:4

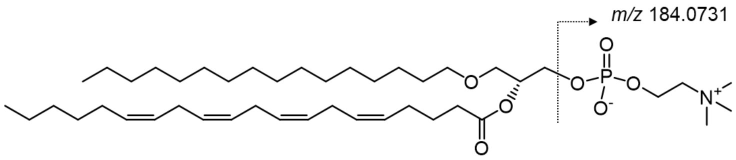

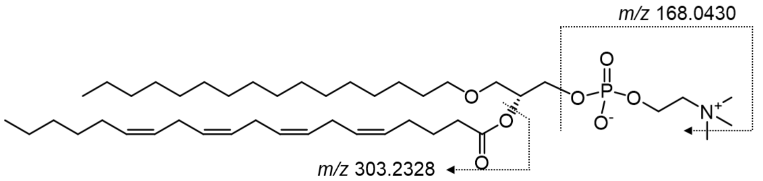

ESI(+)-MS/MS

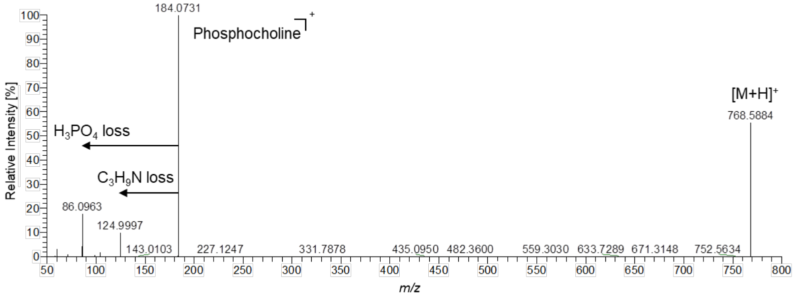

ESI(–)-MS/MS

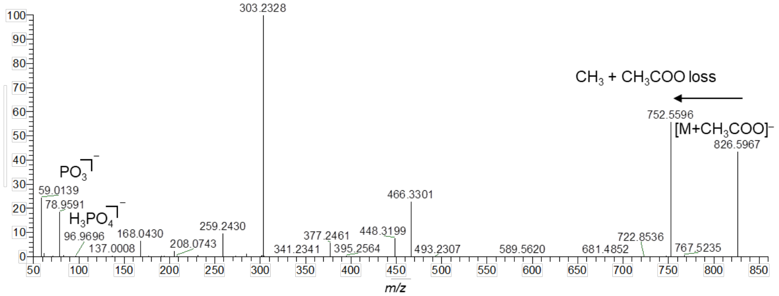

PC P-18:0/20:4

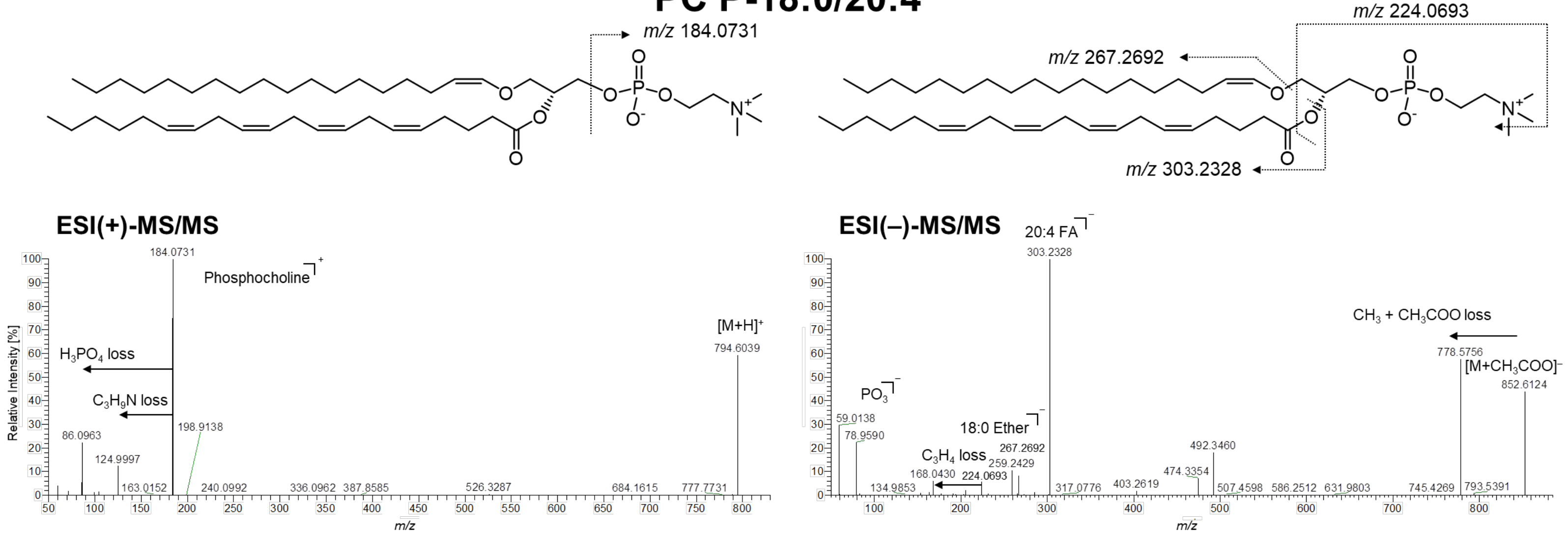

LPC 18:0

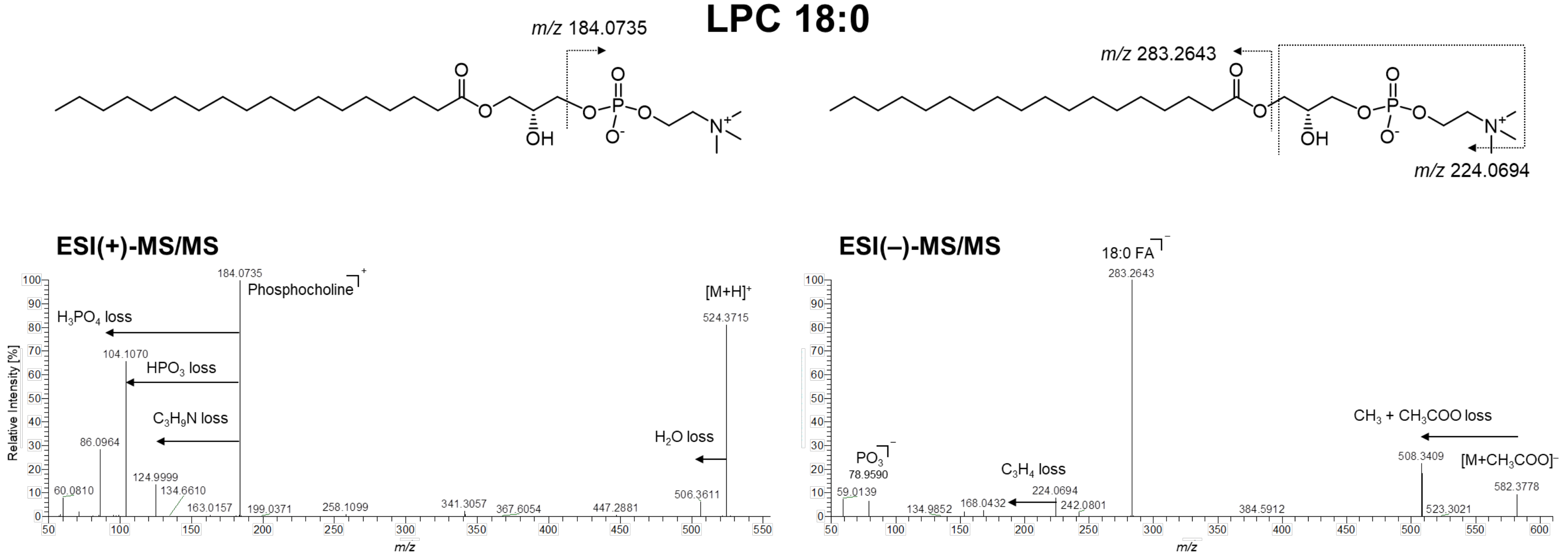

LPC O-18:0

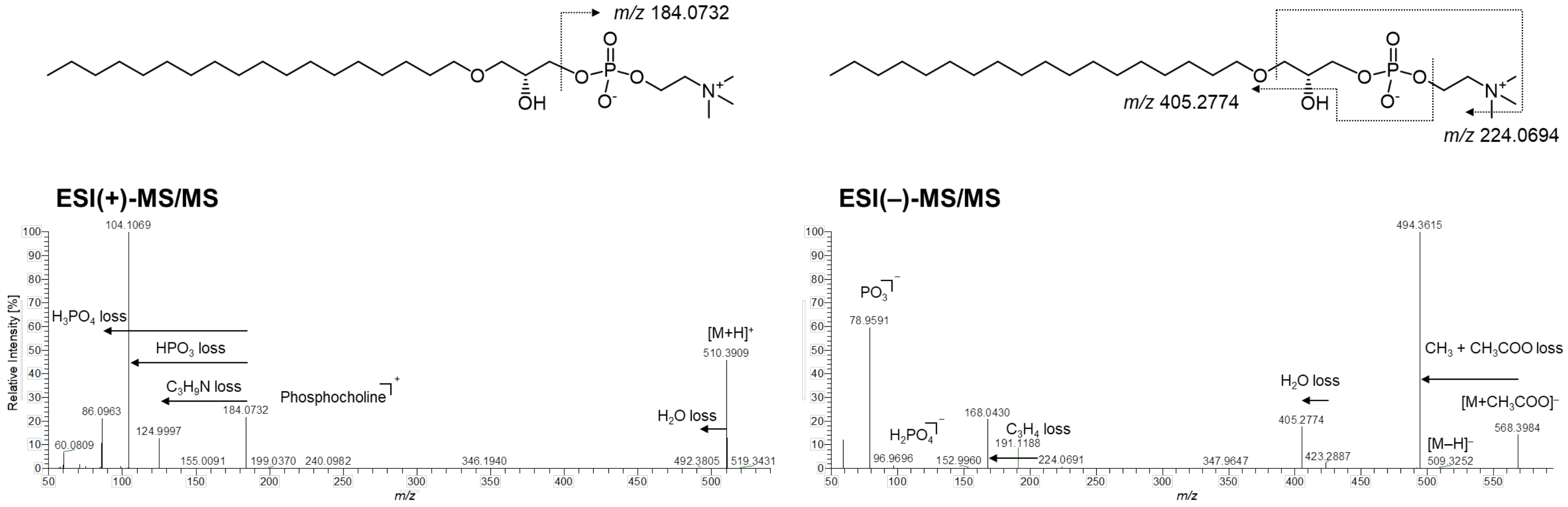

LPC P-18:0

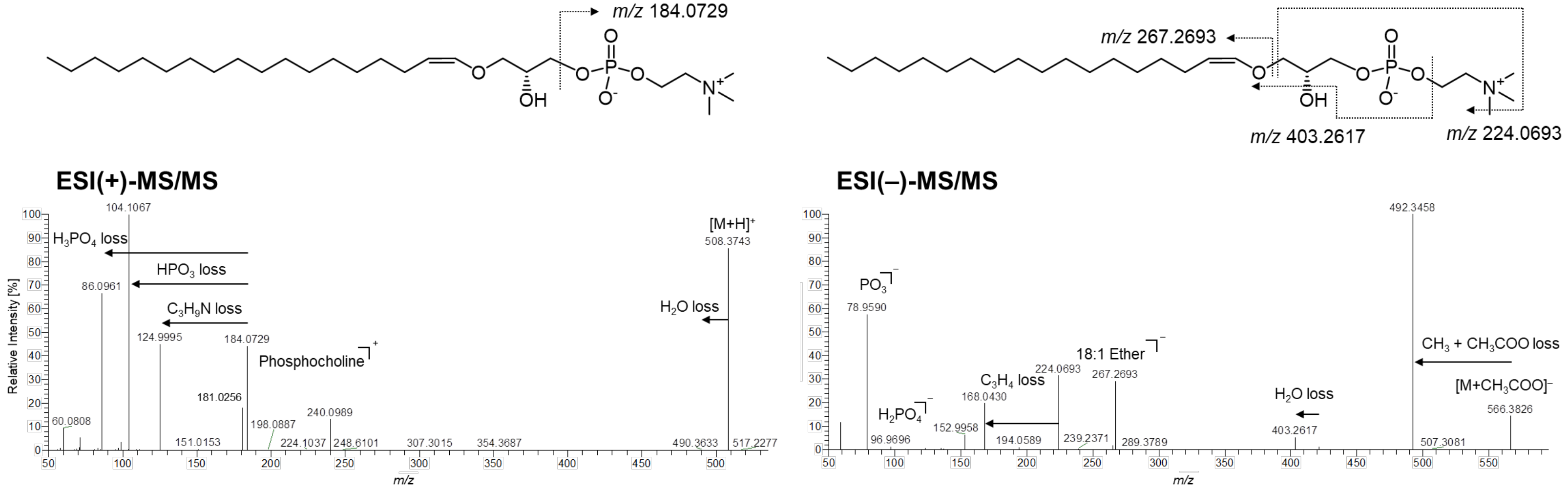

OxPC 16:0/9:0;O

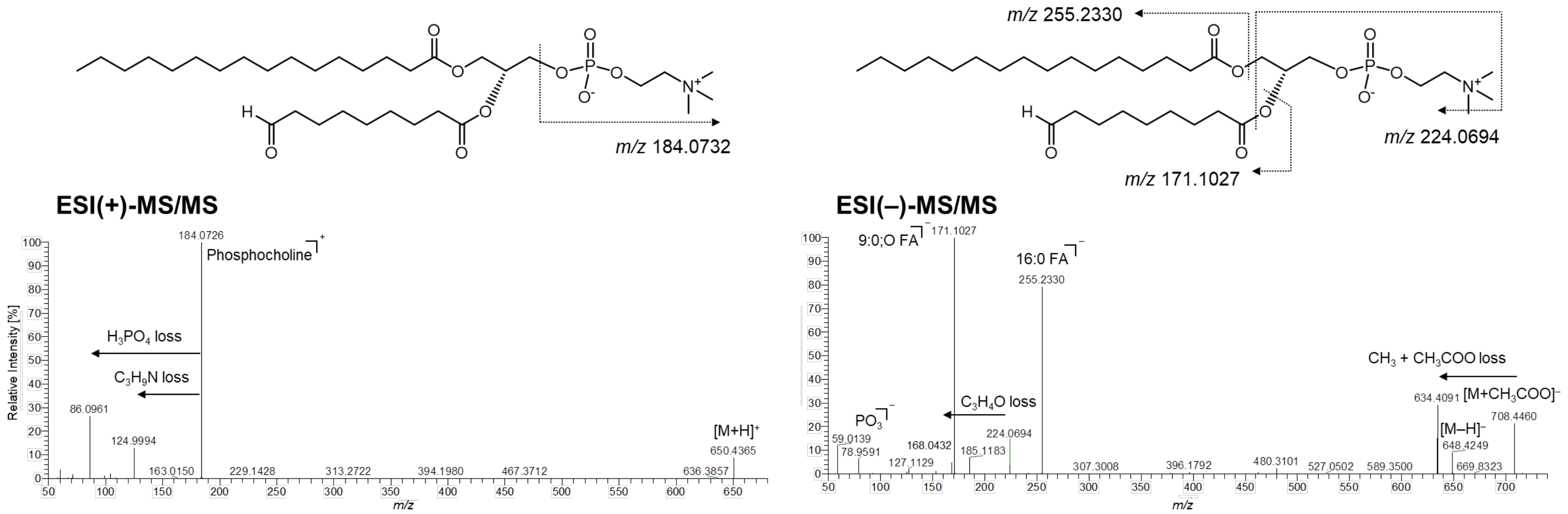

OxPC 16:0/4:0;O2

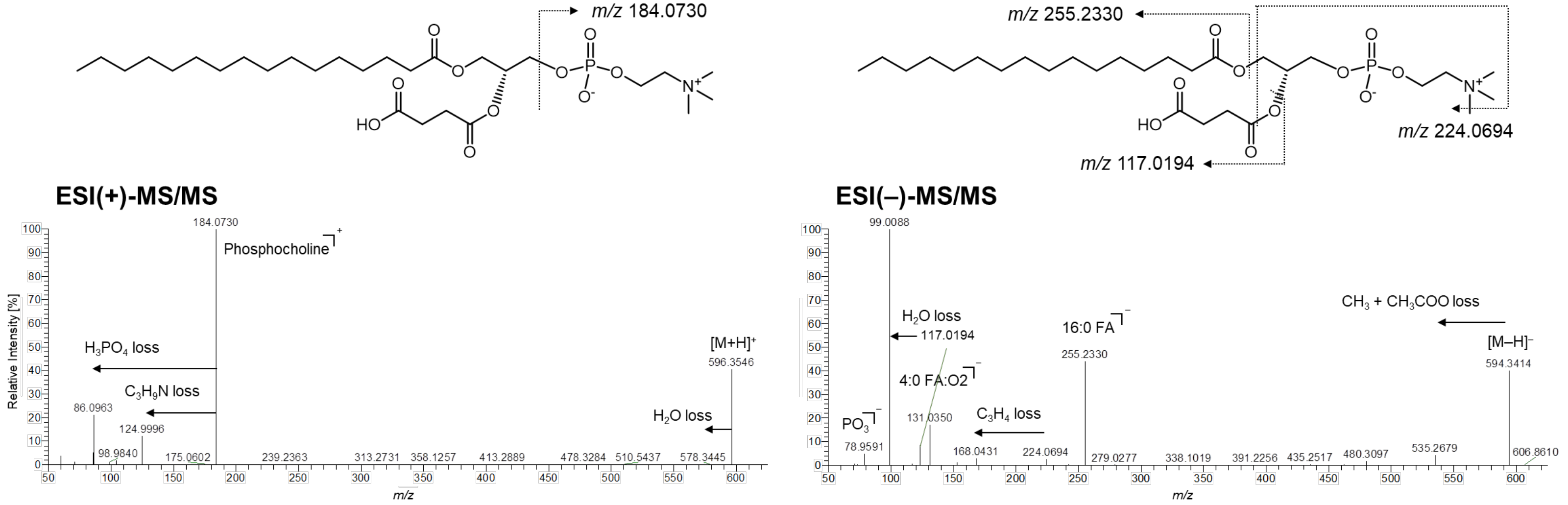

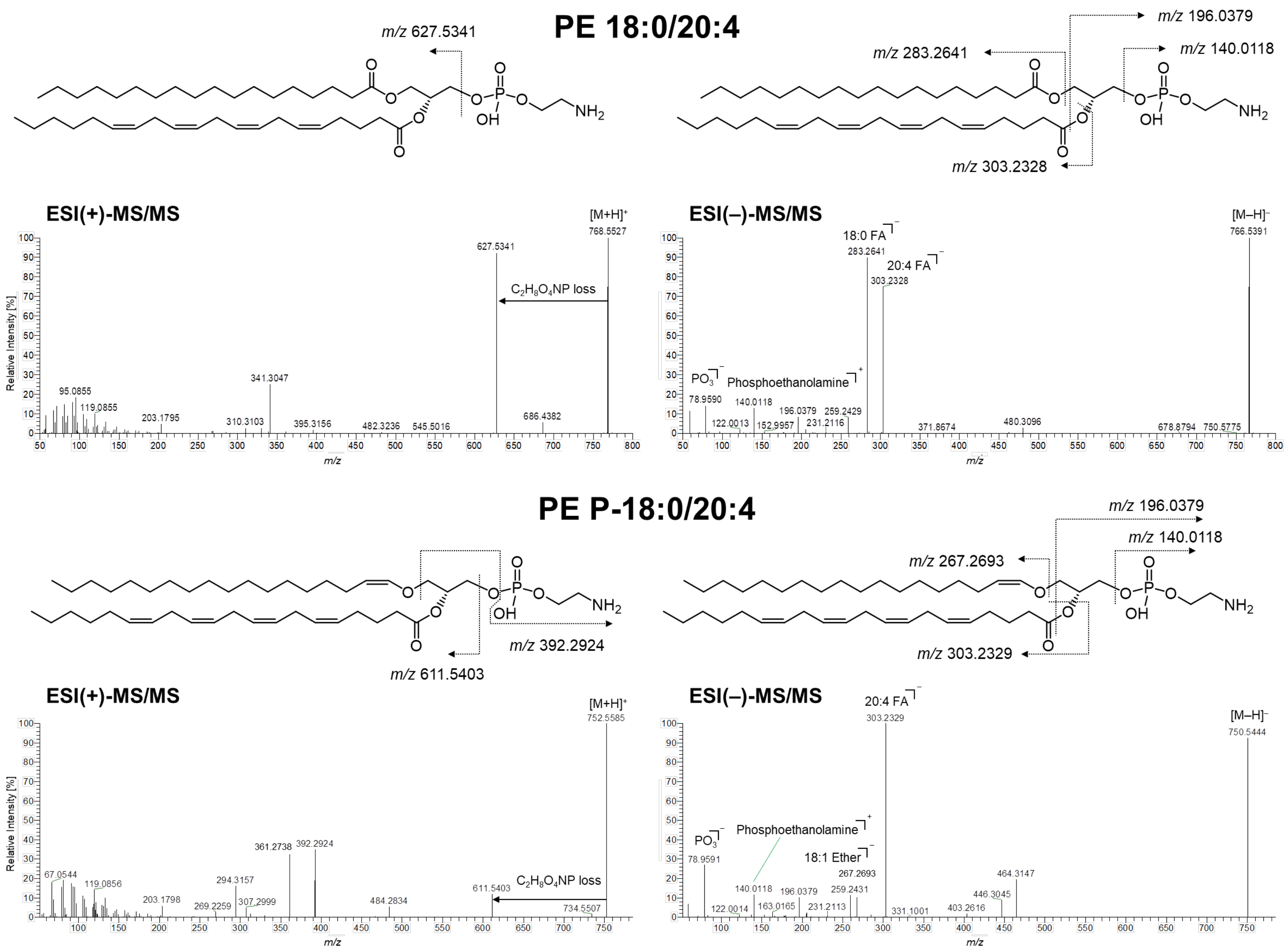

PE 18:0/20:4
m/z 627.5341
m/z 196.0379
m/z 283.2641
m/z 140.0118
NH2
OH
m/z 303.2328
ESI(+)-MS/MS
[M+H]+
768.5527
627.5341
C2H8O4NP loss
Relative Intensity [%]
95.0855
119.0855
203.1795
310.3103
341.3047
395.3156
482.3236
545.5016
686.4382
m/z
ESI(–)-MS/MS
[M–H]–
766.5391
18:0 FA
283.2641
20:4 FA
303.2328
PO3
Phosphoethanolamine
78.9590
122.0013
140.0118
152.9957
196.0379
231.2116
259.2429
371.8674
480.3096
678.8794
750.5775
PE P-18:0/20:4
m/z 196.0379
m/z 140.0118
m/z 267.2693
m/z 392.2924
m/z 611.5403
m/z 303.2329
ESI(+)-MS/MS
[M+H]+
752.5585
67.0544
119.0856
203.1798
269.2259
294.3157
307.2999
361.2738
392.2924
484.2834
611.5403
C2H8O4NP loss
734.5507
ESI(–)-MS/MS
20:4 FA
303.2329
[M–H]–
750.5444
PO3
Phosphoethanolamine
18:1 Ether
78.9591
122.0014
140.0118
163.0165
196.0379
231.2113
259.2431
267.2693
331.1001
403.2616
446.3045
464.3147

MPE 16:0/16:0

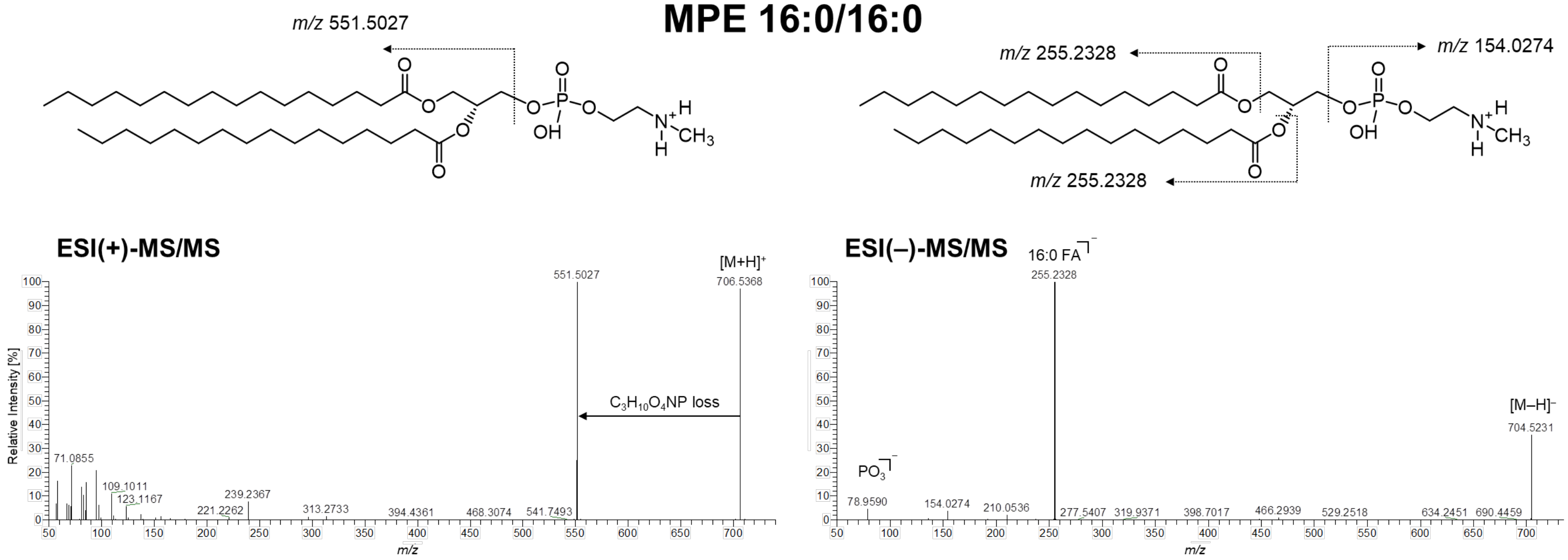

LPE 18:0

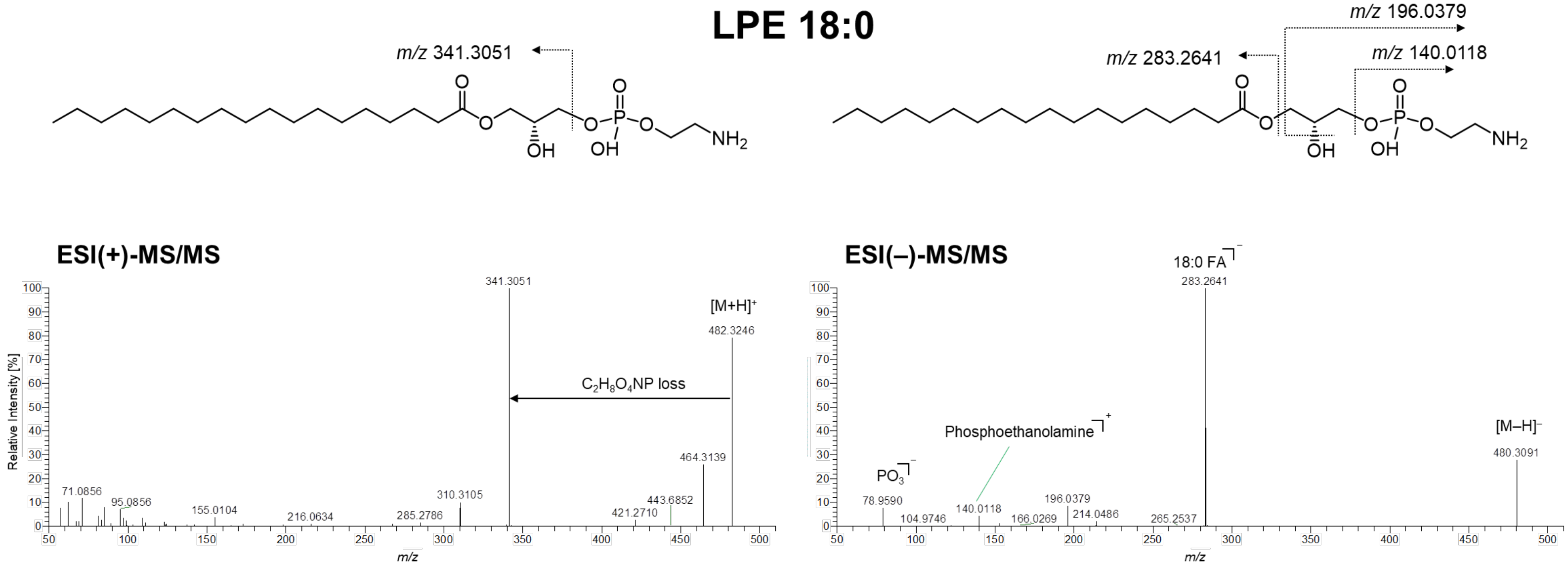

LPE P-18:0

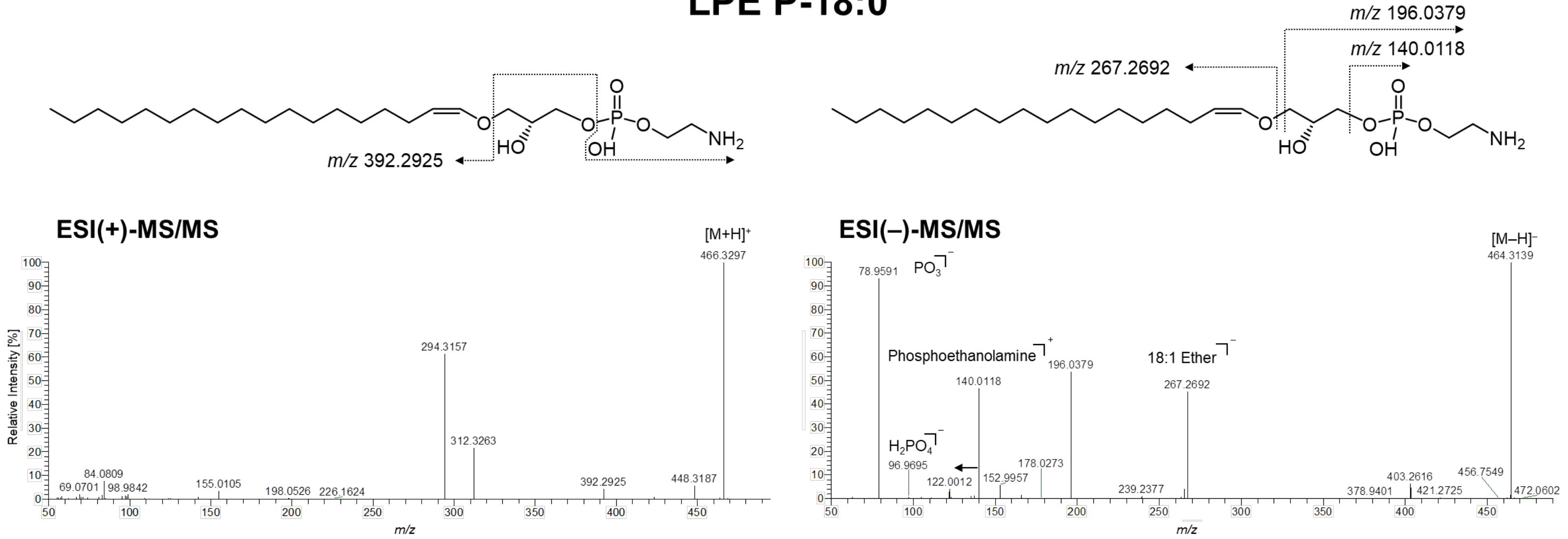

Lac-PE 18:1/18:1

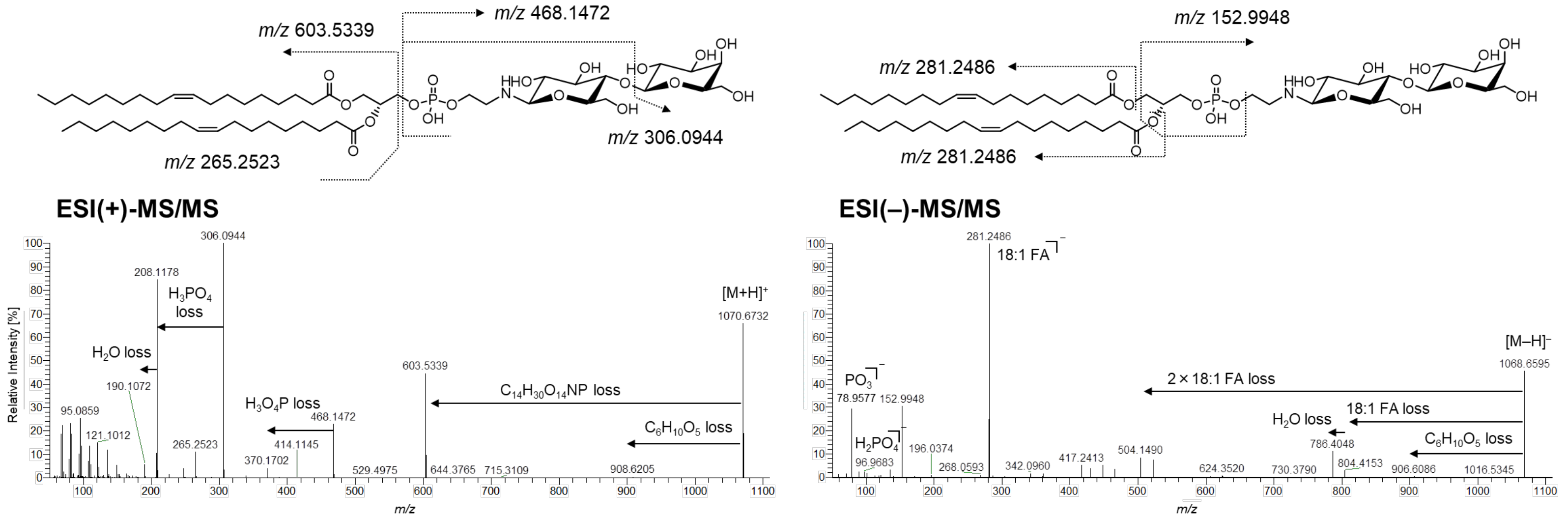

Suc-PE 18:1/18:1

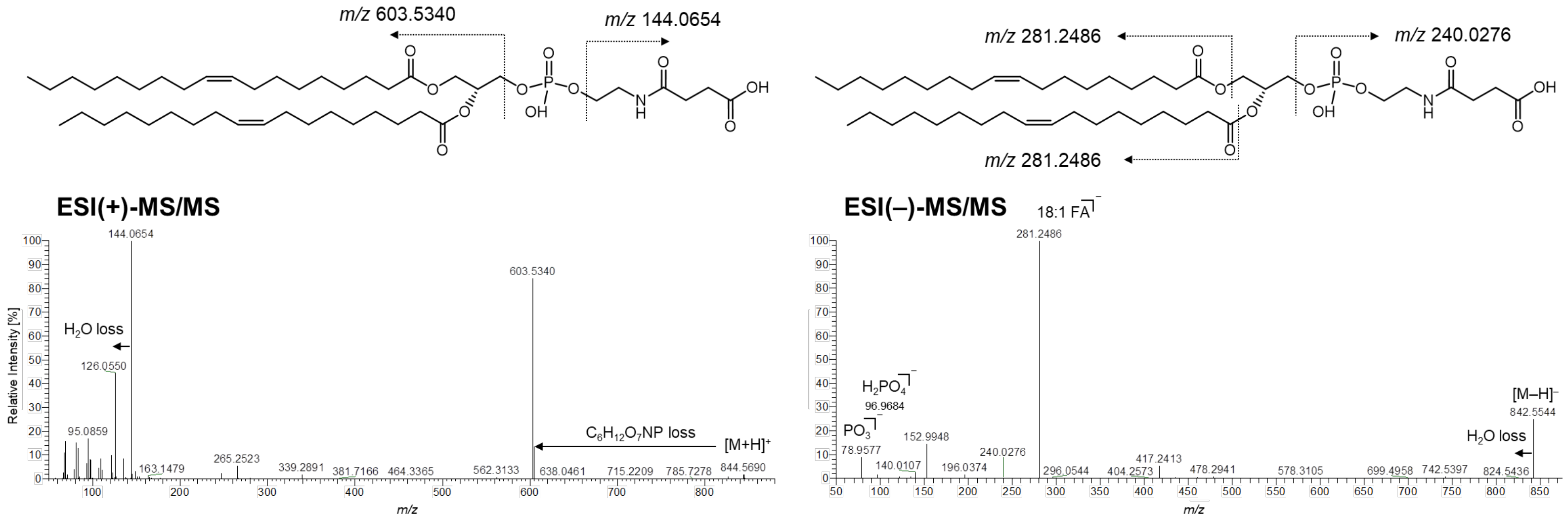

PS 18:0/18:1

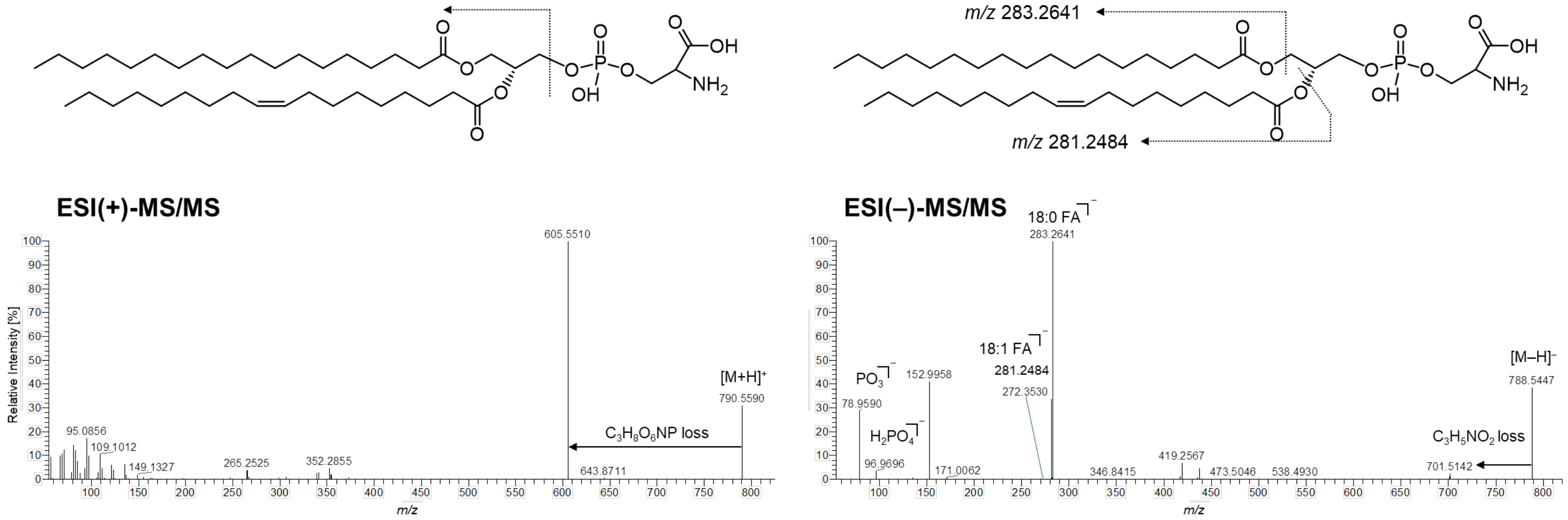

NAPS 18:1/18:1/19:0

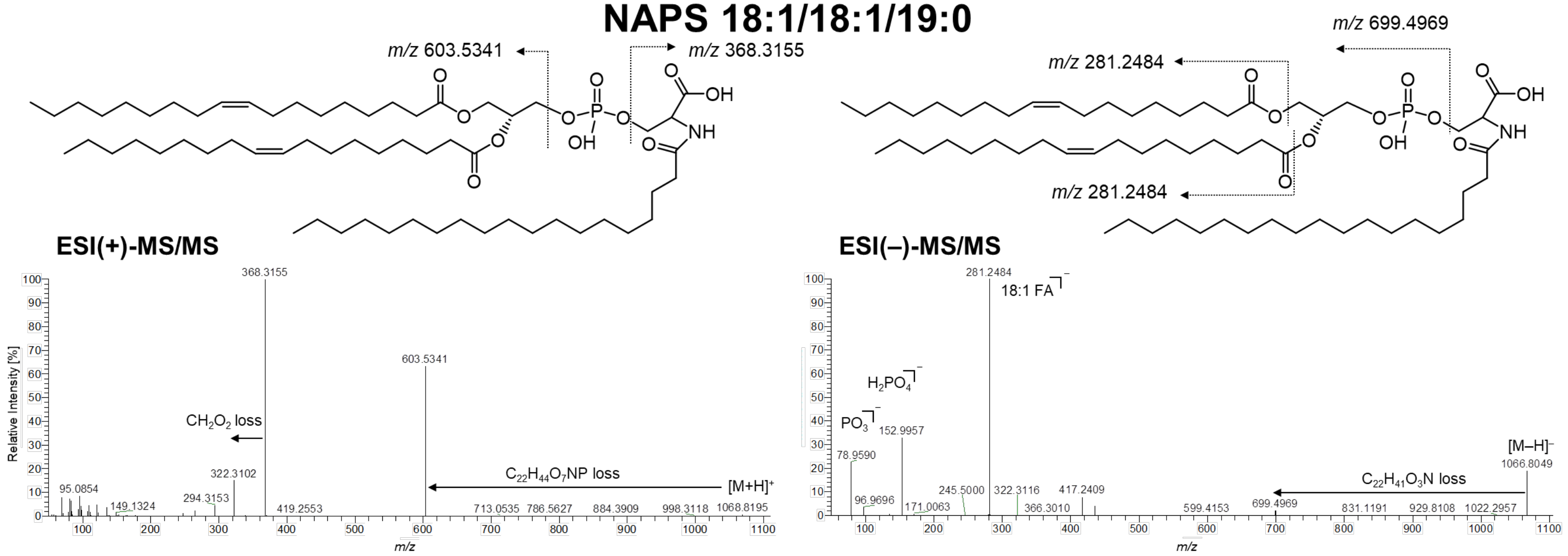

LPS 16:0

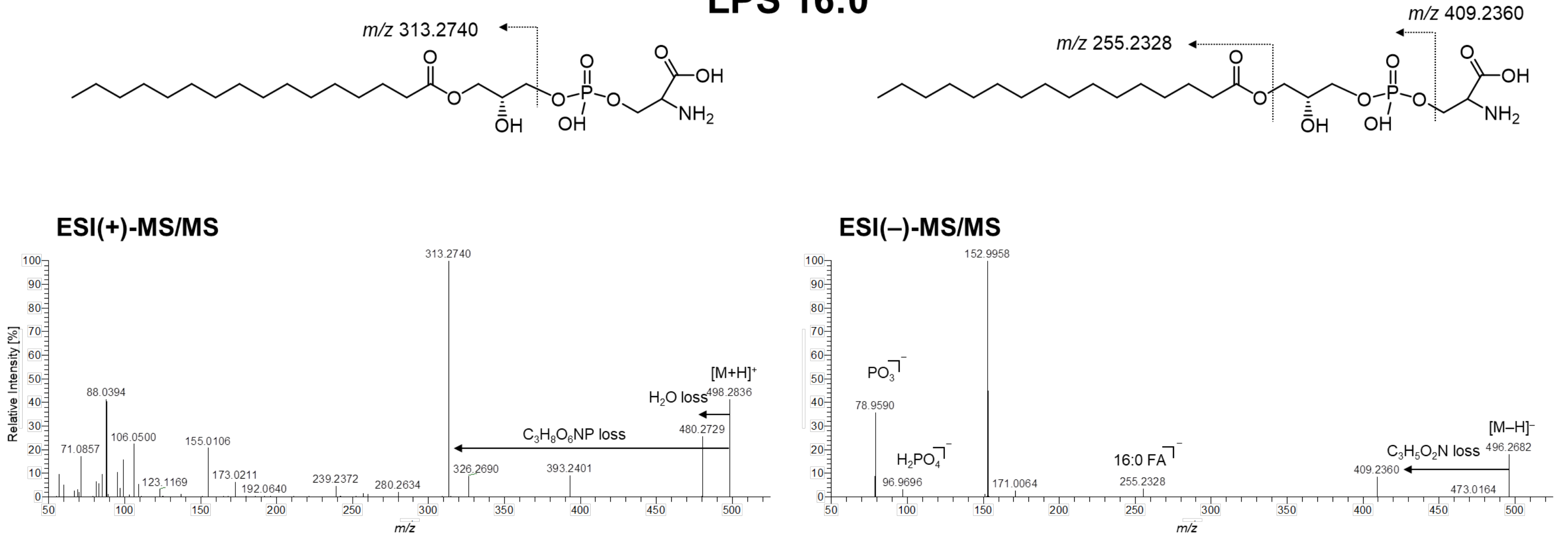

PG 16:0/18:1

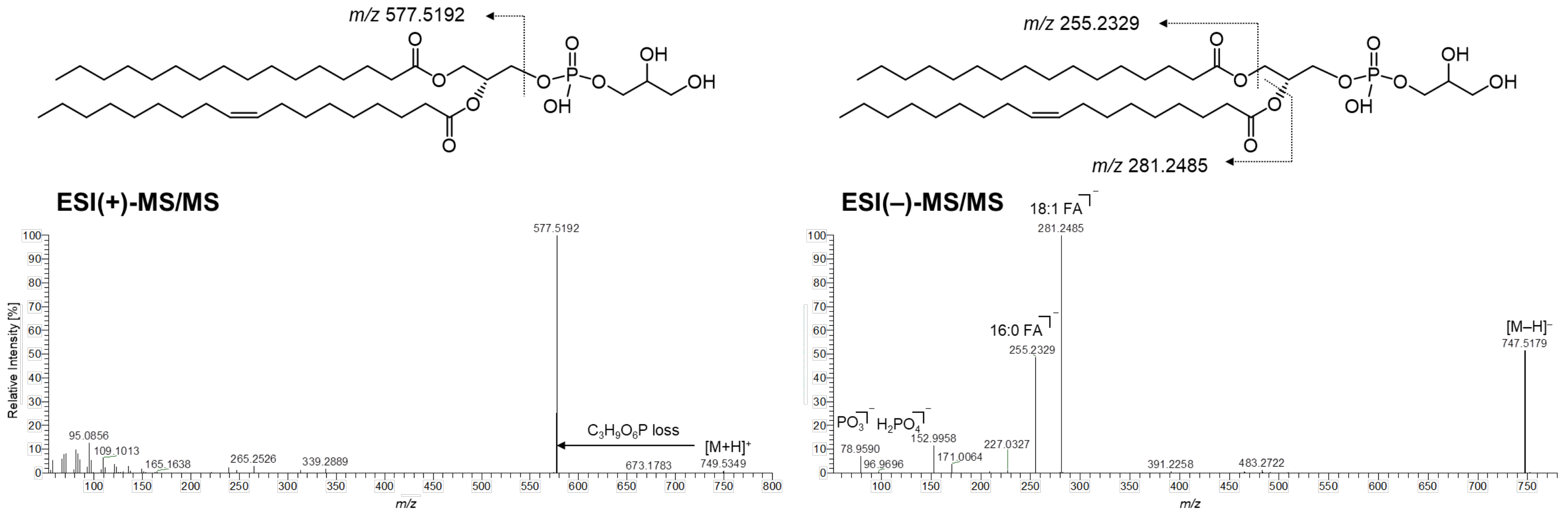

Lysyl-PG 16:0/16:0

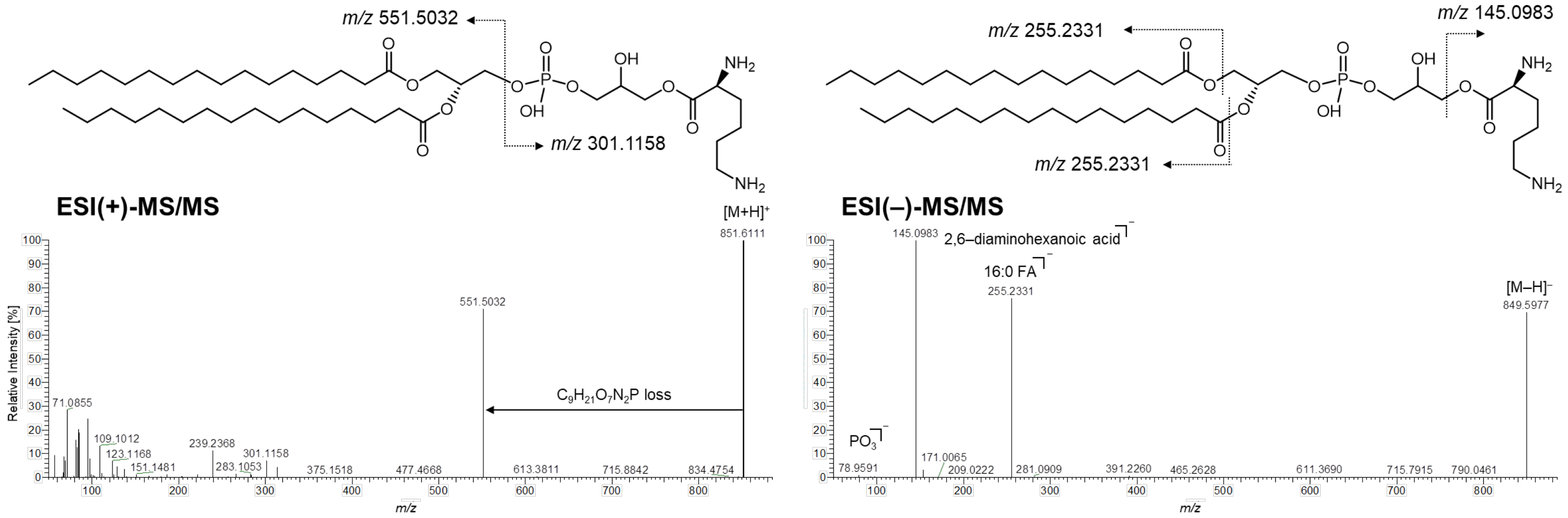

LPG 18:0

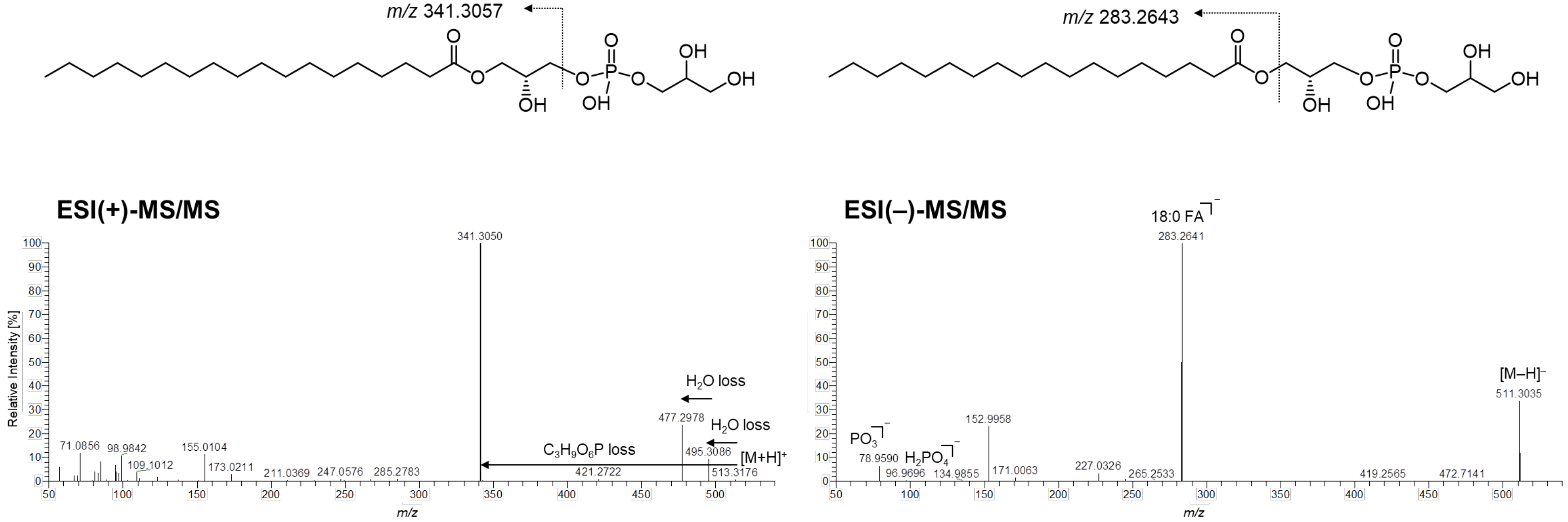

SLBPA 18:1/18:1/16:0

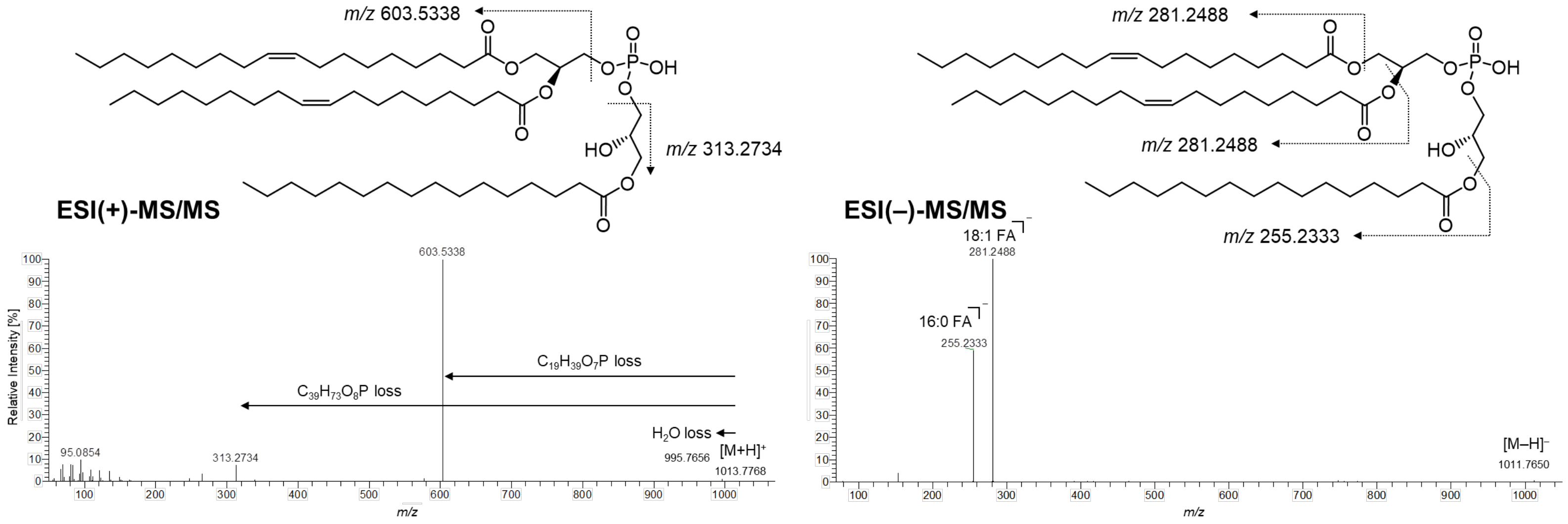

LBPA 18:1/18:1

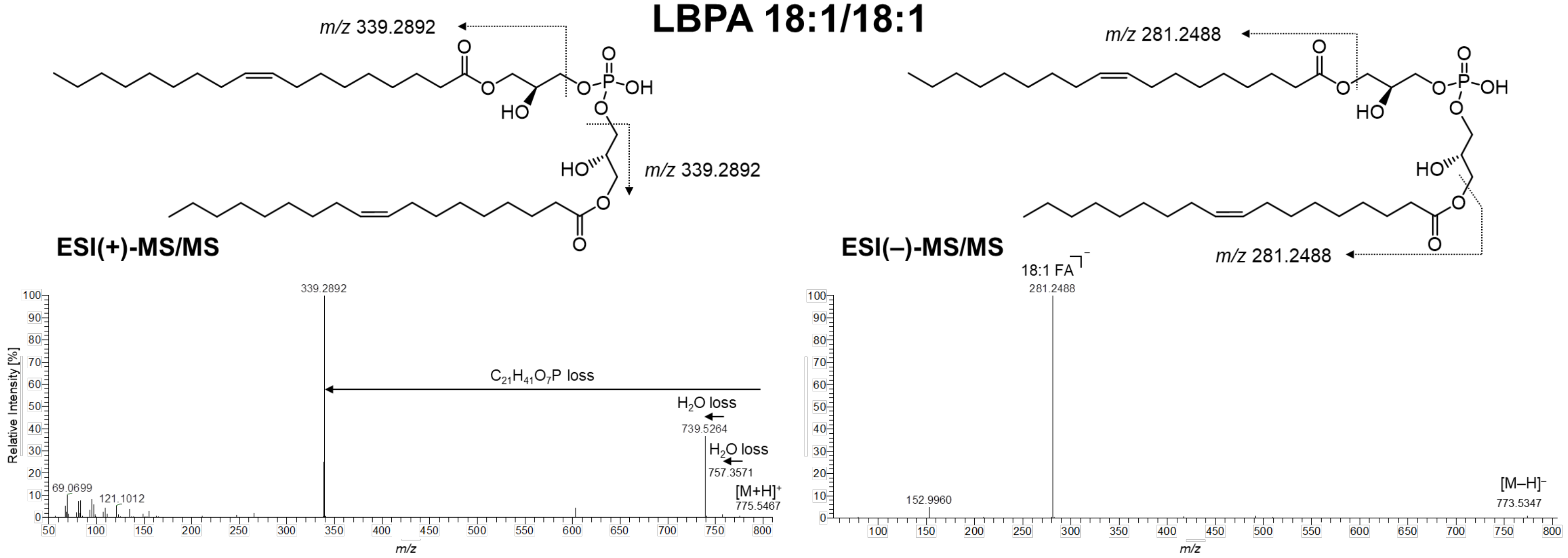

BPA 18:1/18:1/18:1/17:0

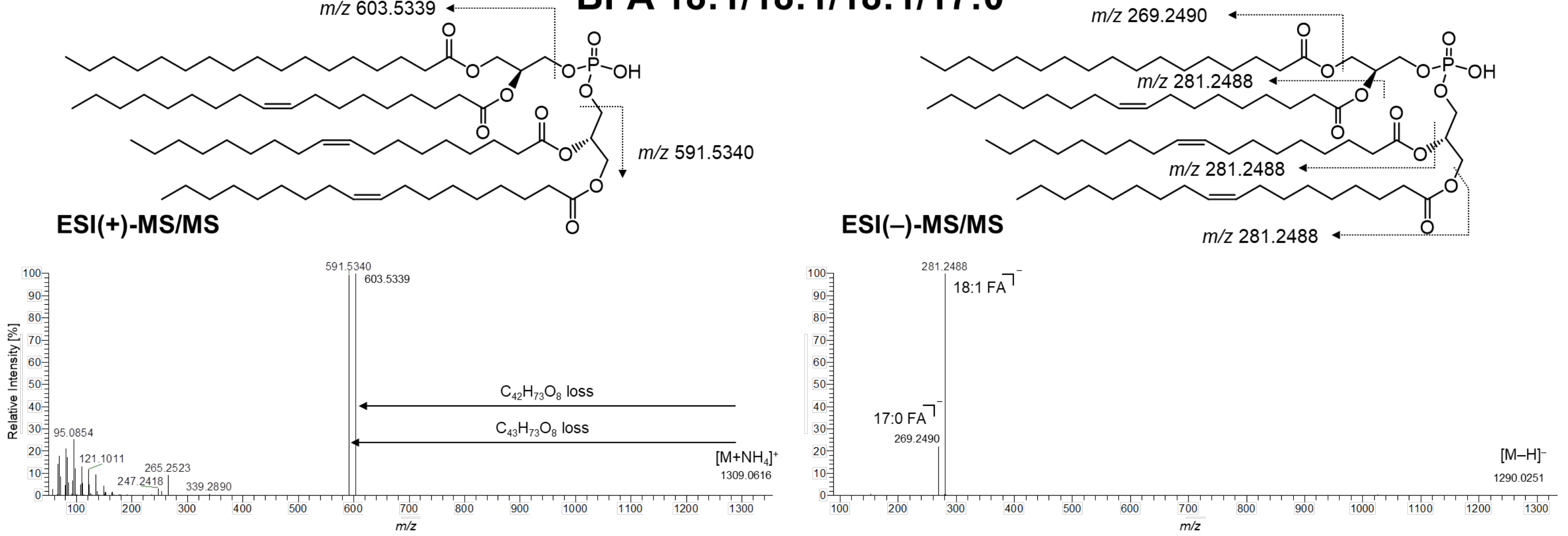

PI 18:0/20:4

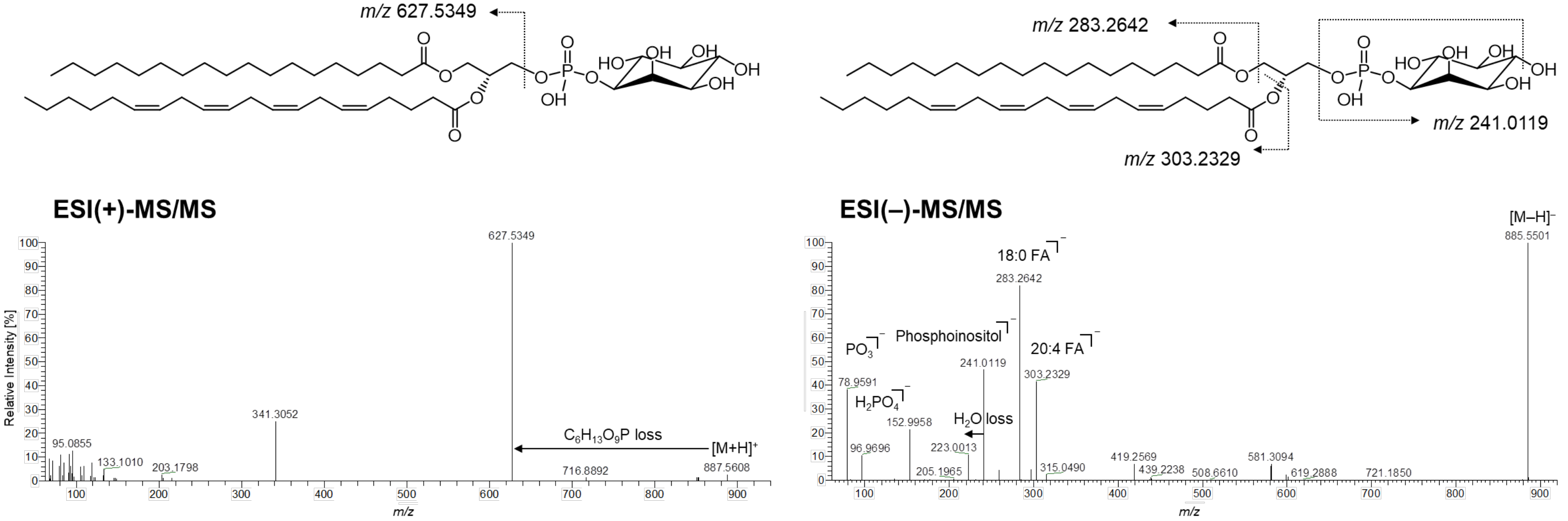

LPI 18:0

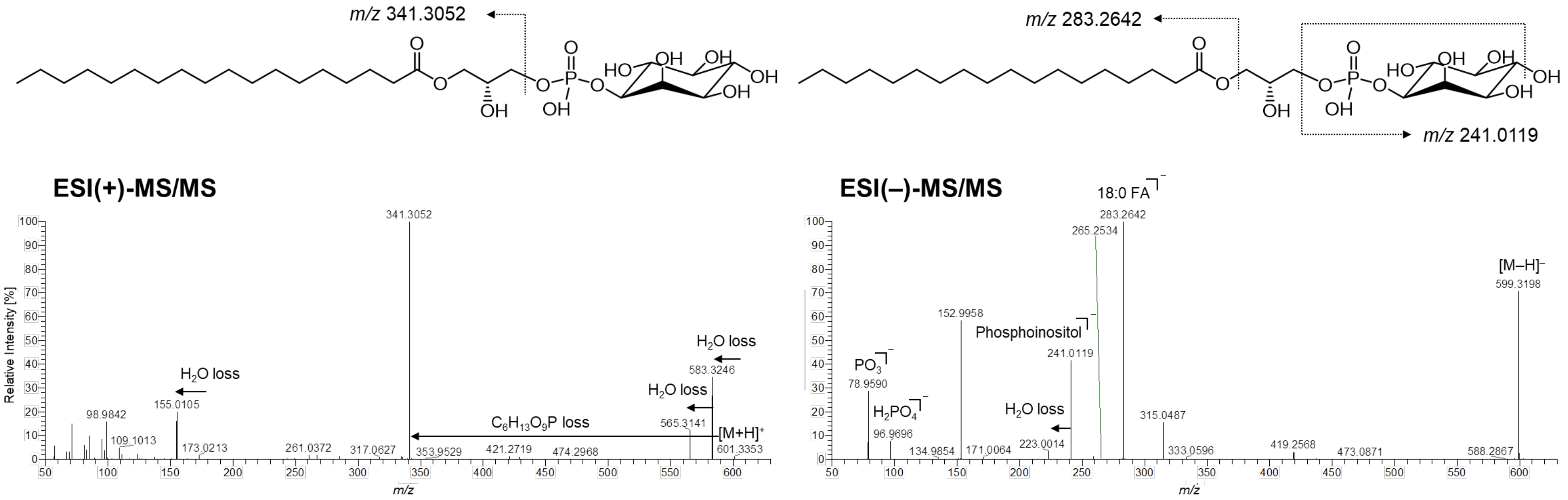

PIP 18:1/18:1

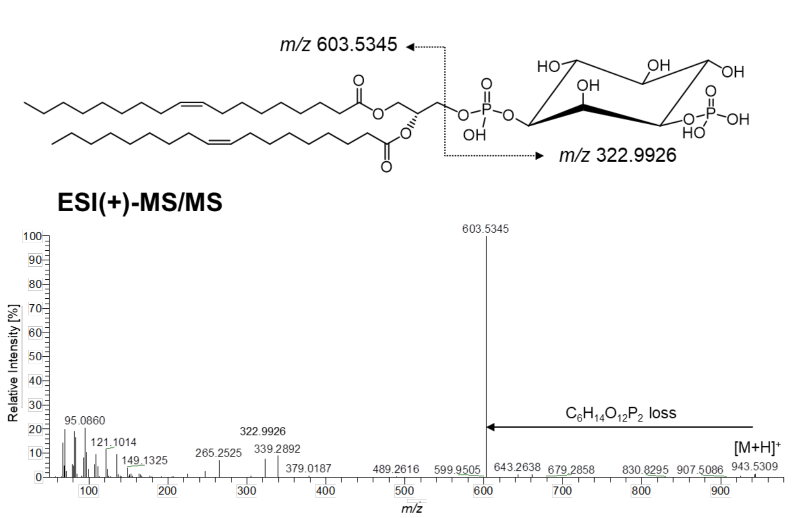

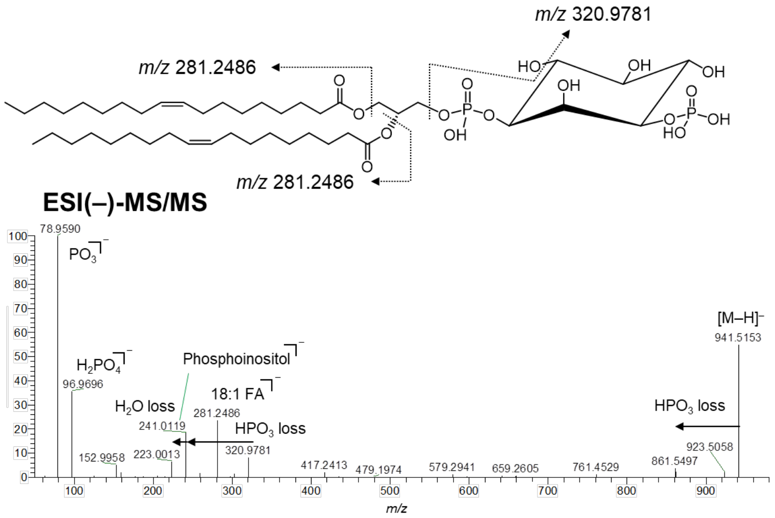

PIP2 18:1/18:1

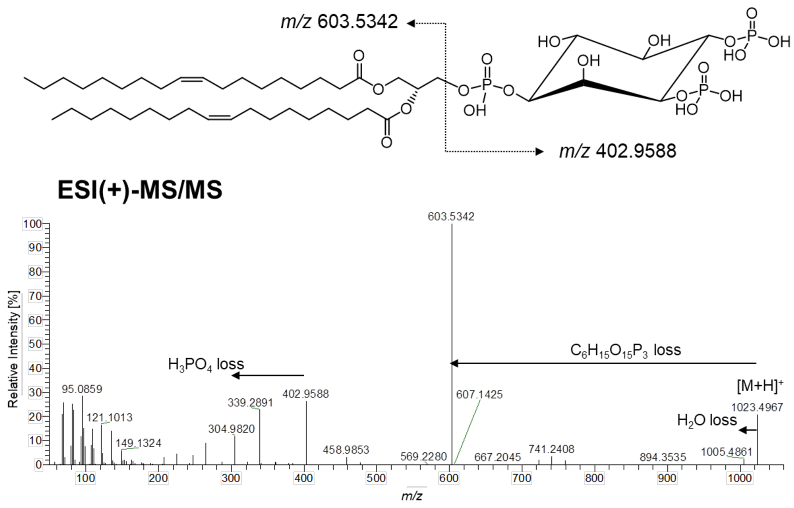

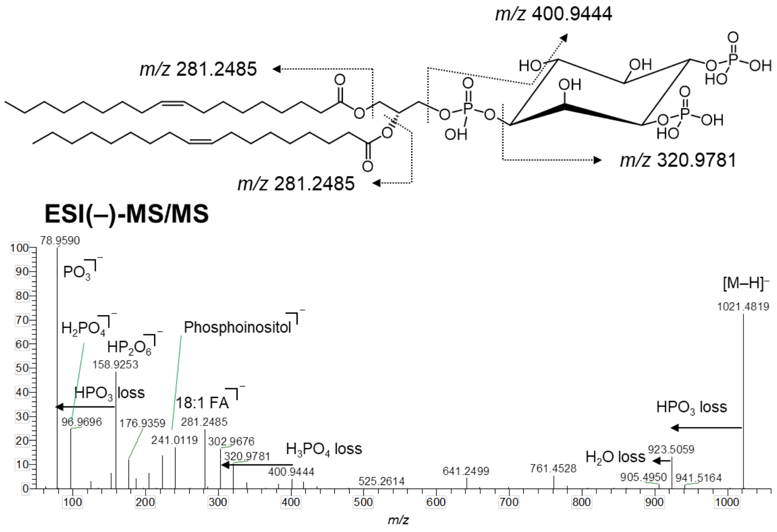

PIP3 16:0/16:0

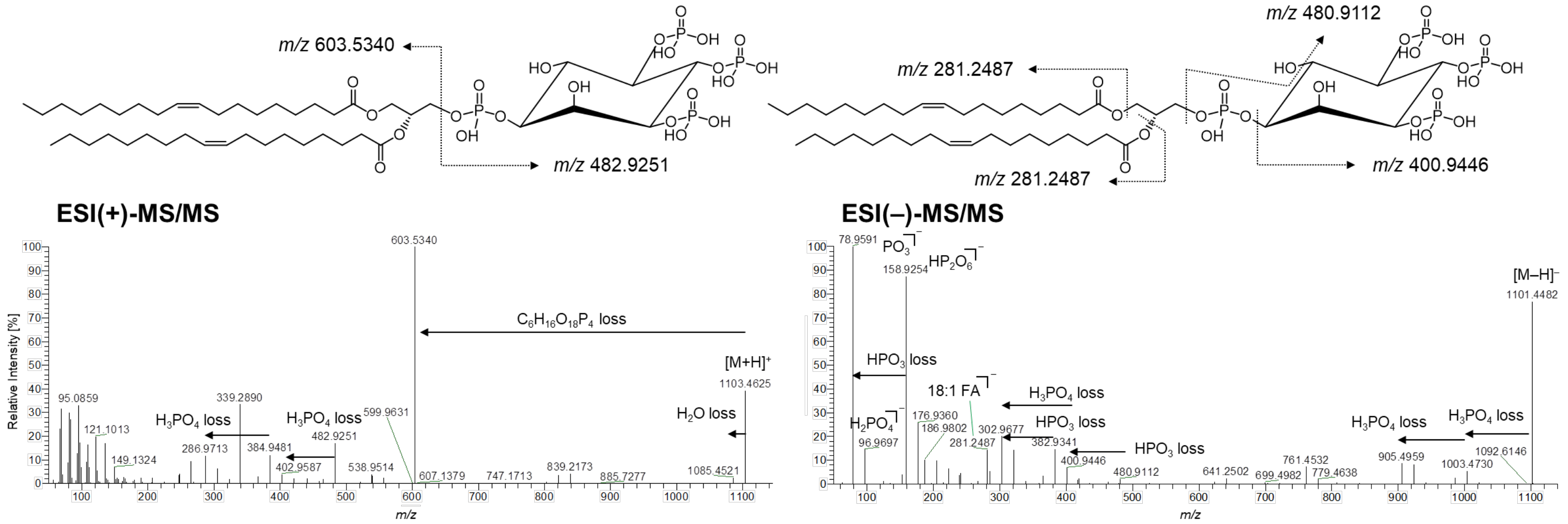

PA 16:0/18:1

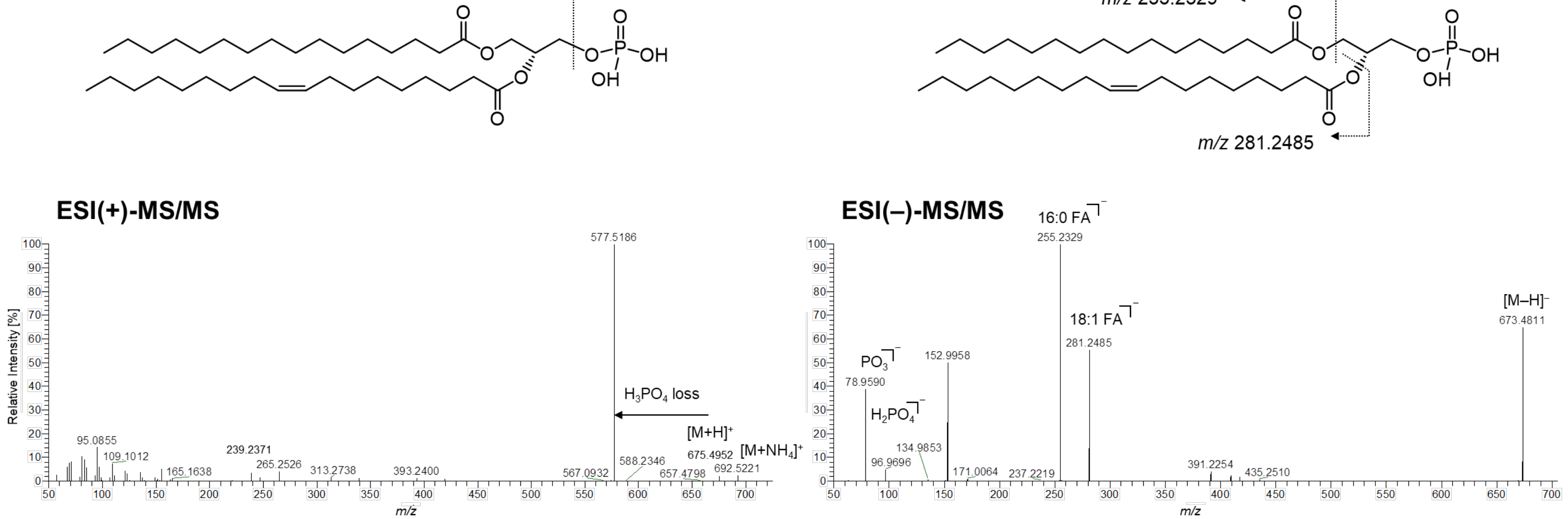

LPA 18:0

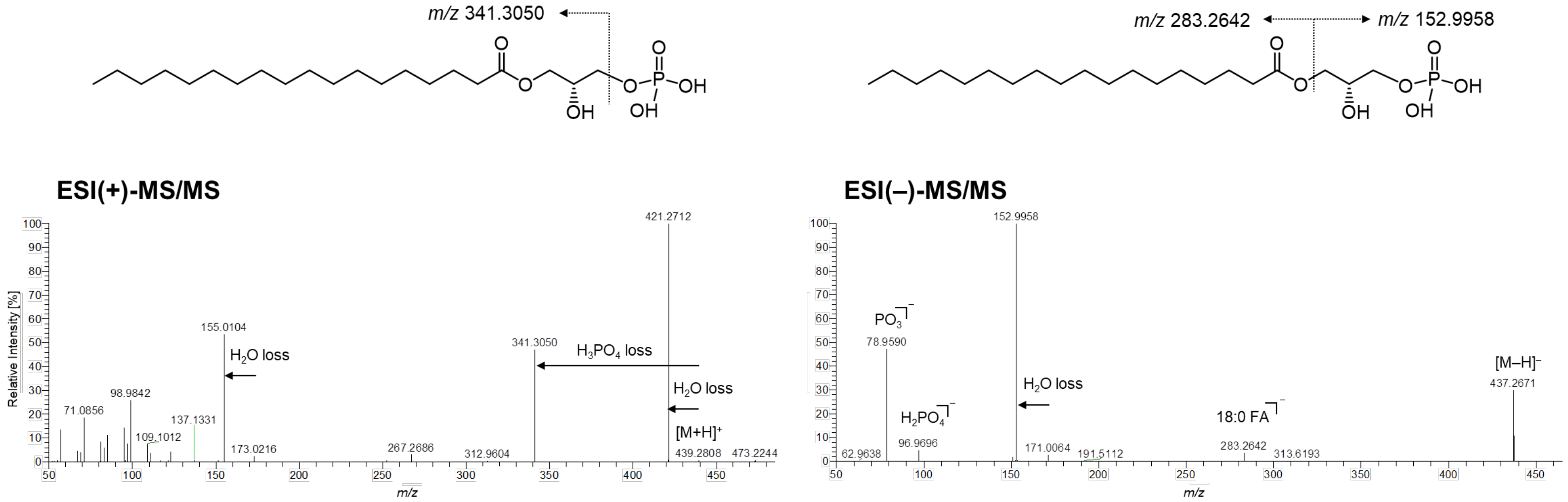

DGPP 18:1/18:1

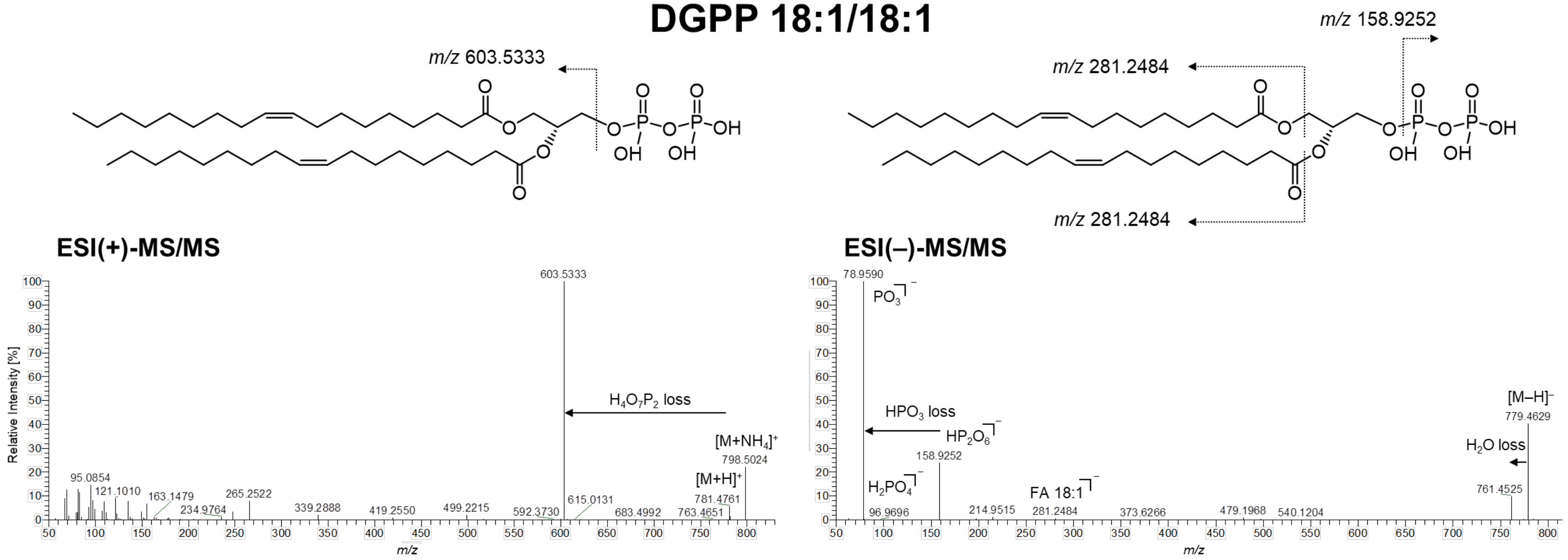

[d_5] CL 18:2/18:2

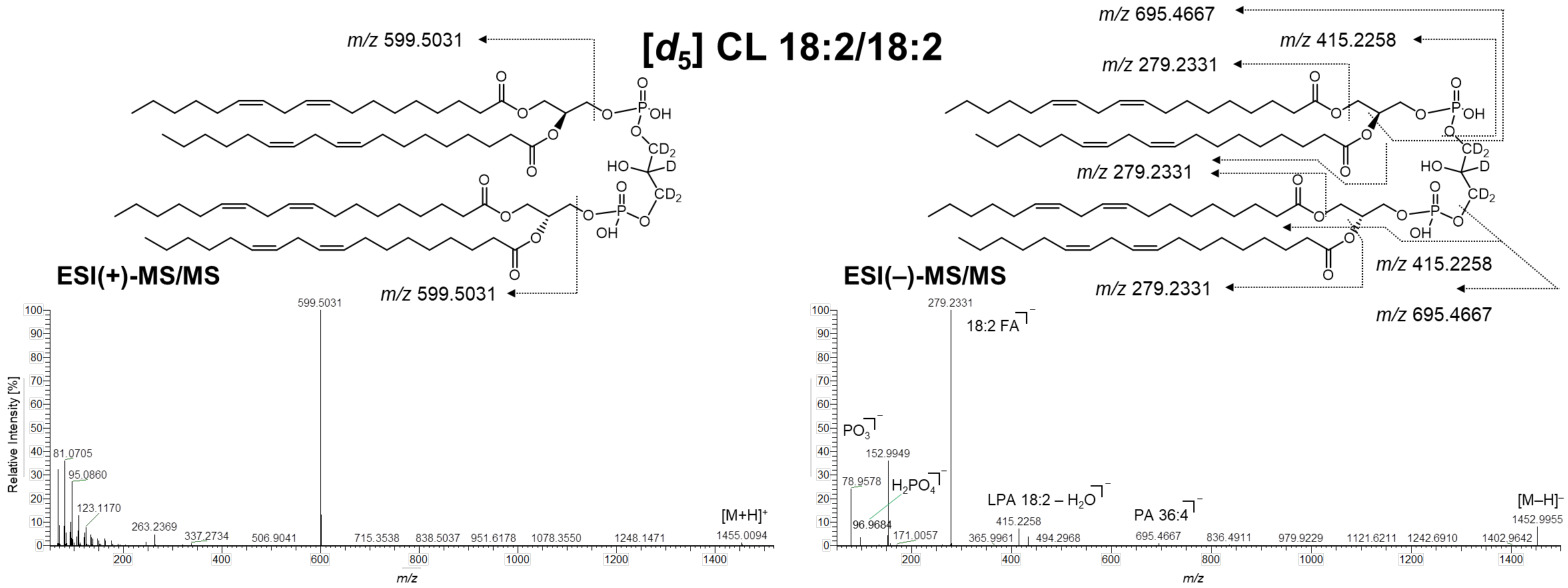

CDP-DG 16:0/16:0

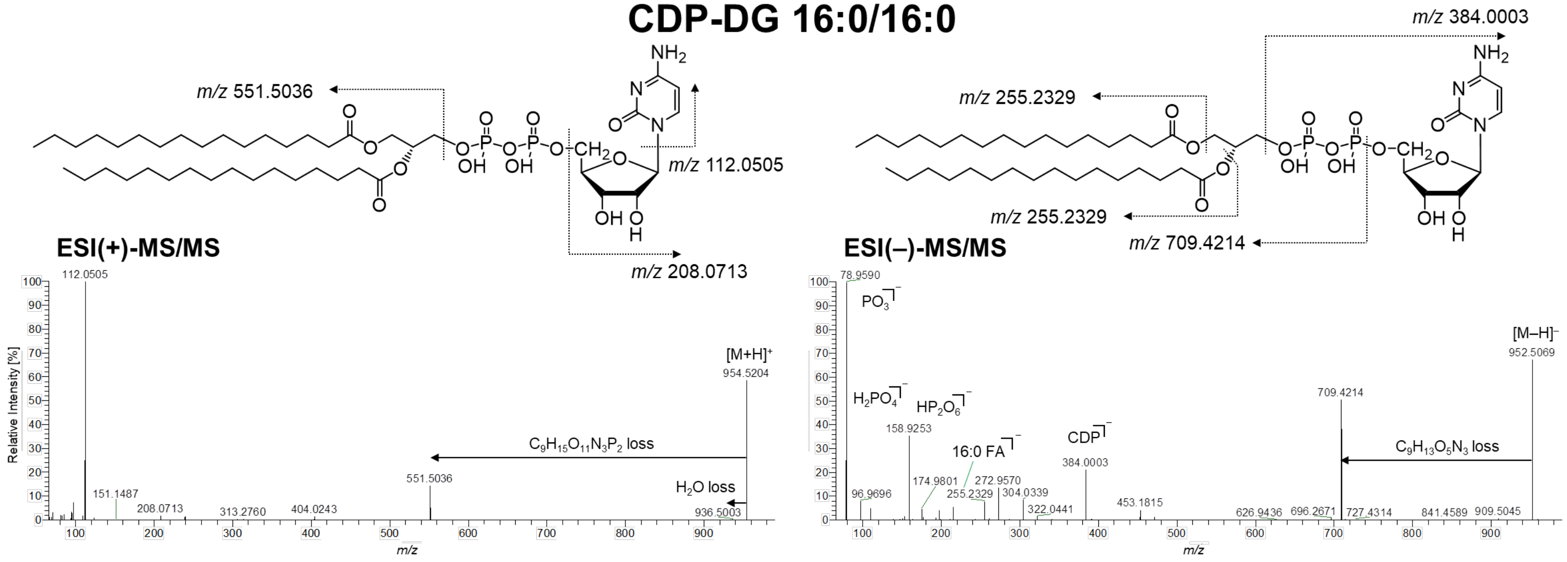

CPA 17:0

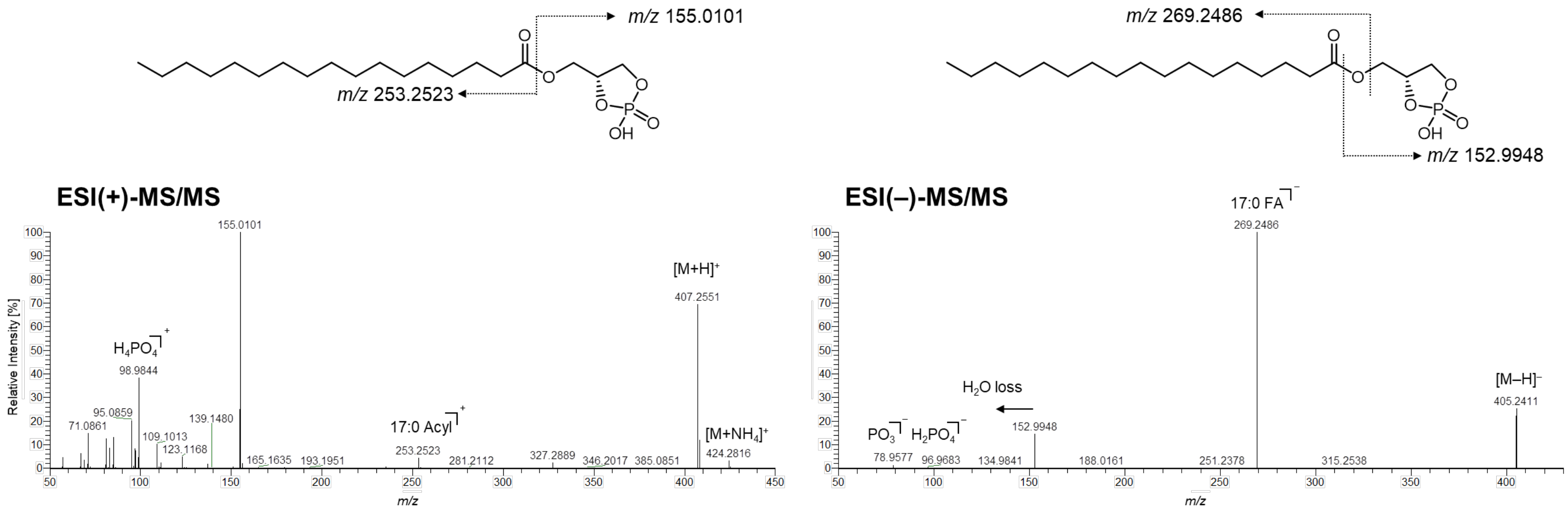

PTE 16:0/16:0

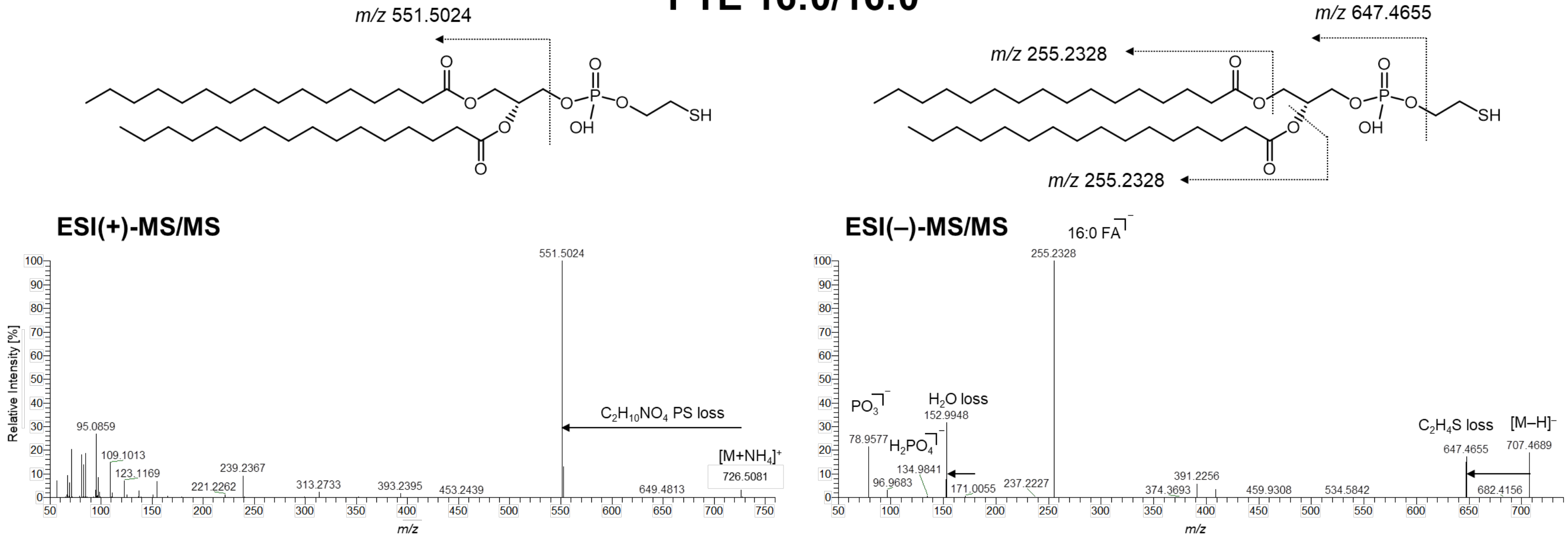

[d_7] SPB d18:1

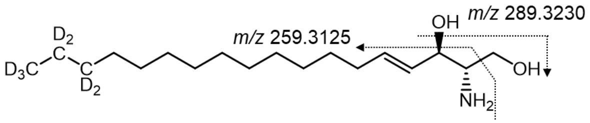

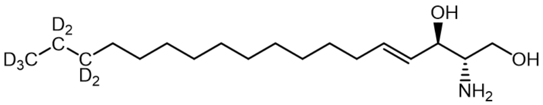

ESI(+)-MS/MS

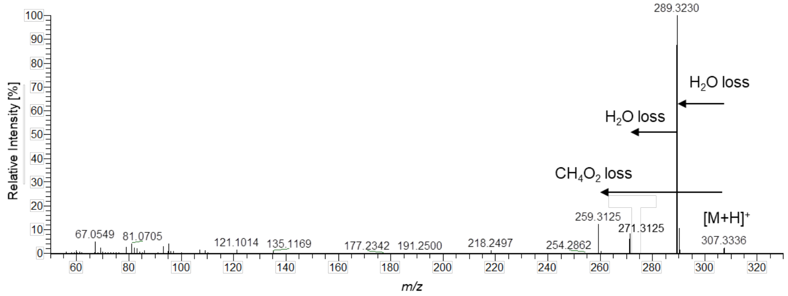

ESI(–)-MS/MS

[d_7] SPB d18:0

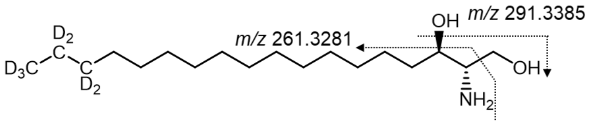

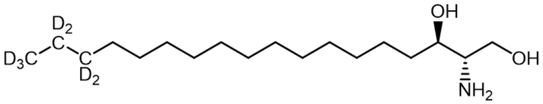

ESI(+)-MS/MS

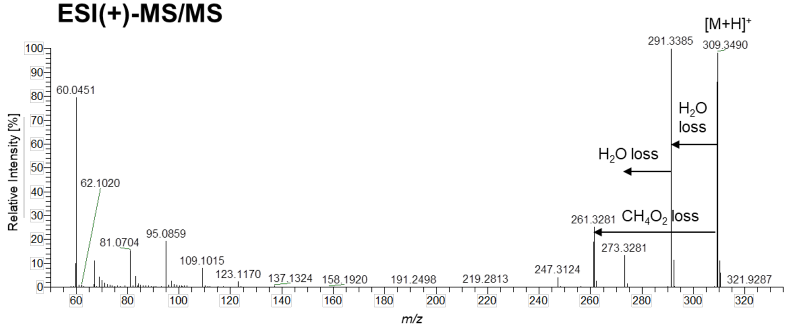

ESI(–)-MS/MS

[d_7] SPBP d18:1

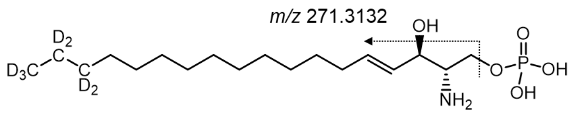

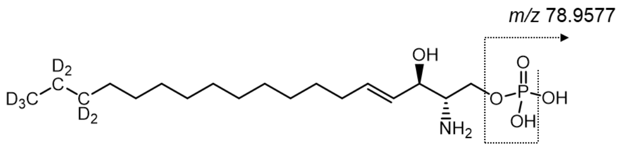

ESI(+)-MS/MS

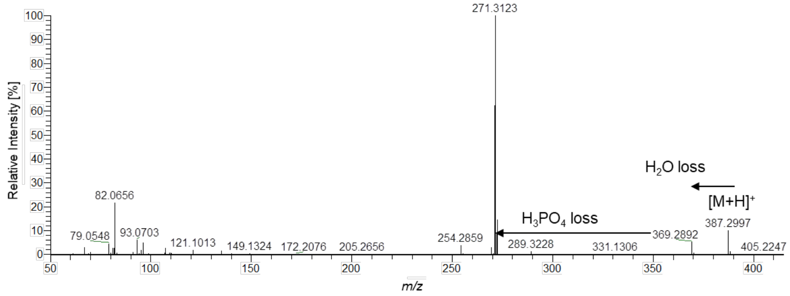

ESI(–)-MS/MS

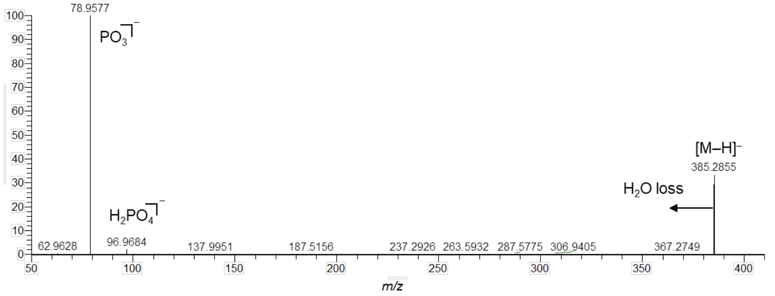

[d_7] SPBP d18:0

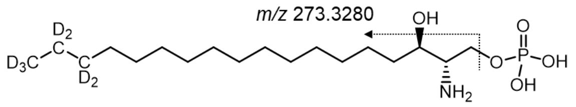

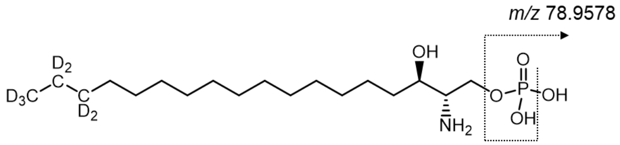

ESI(+)-MS/MS

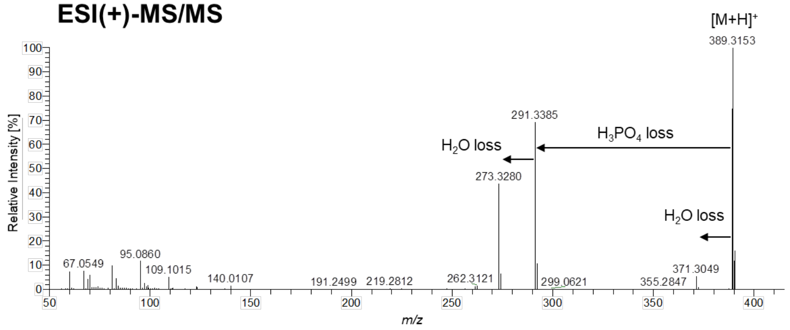

ESI(–)-MS/MS

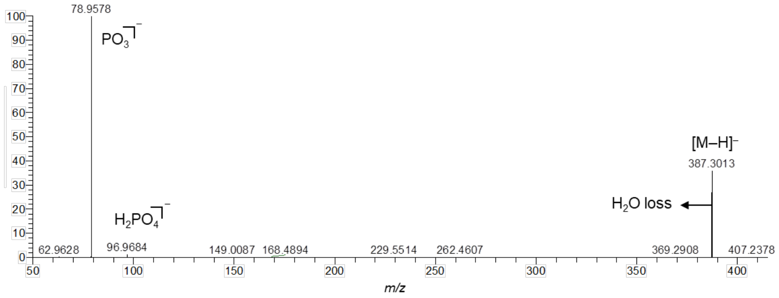

SPB m18:0

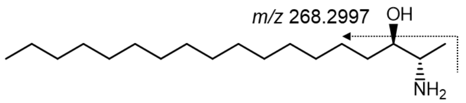

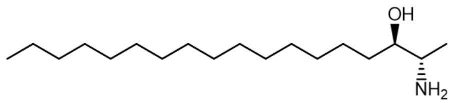

ESI(+)-MS/MS

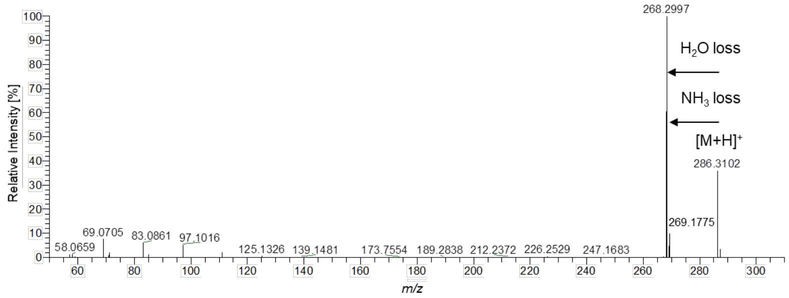

ESI(–)-MS/MS

N.D.

1-desoxymethyl SPB m17:0

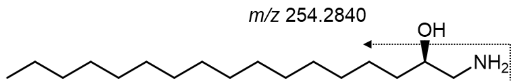

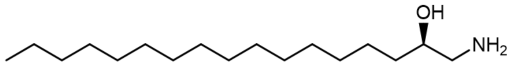

ESI(+)-MS/MS

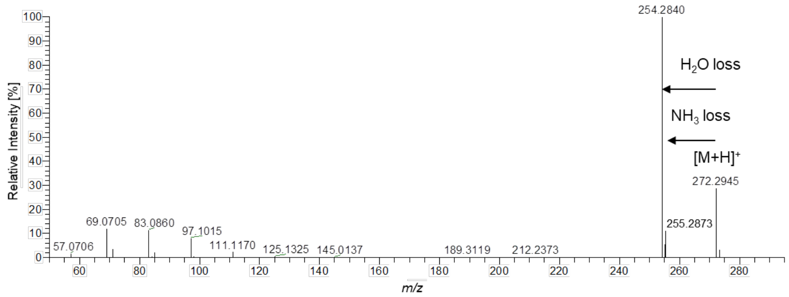

ESI(–)-MS/MS

N.D.

N,N-Dimethyl-SPB d17:1

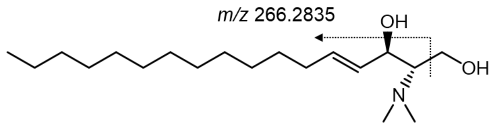

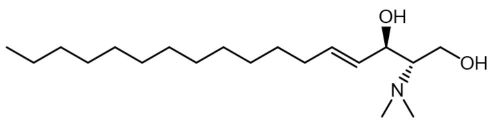

ESI(+)-MS/MS

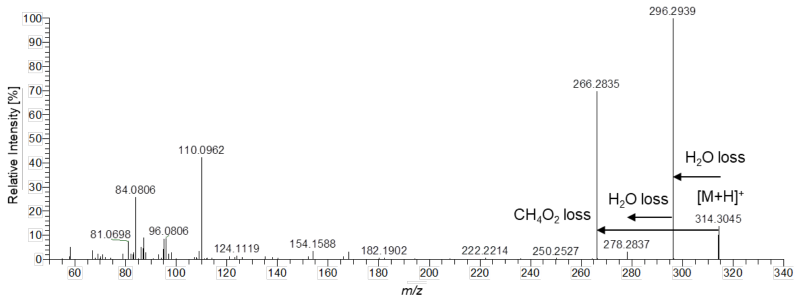

ESI(–)-MS/MS

SPBP t18:0

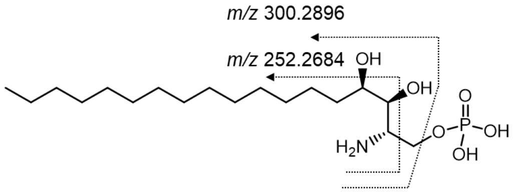

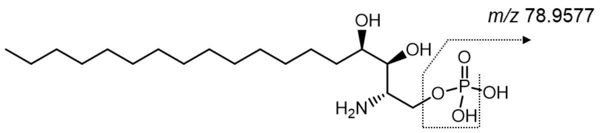

ESI(+)-MS/MS

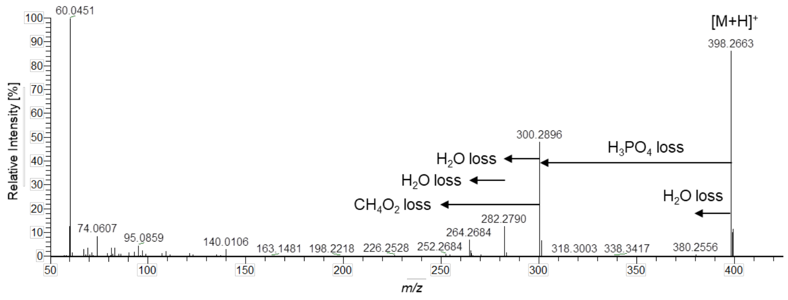

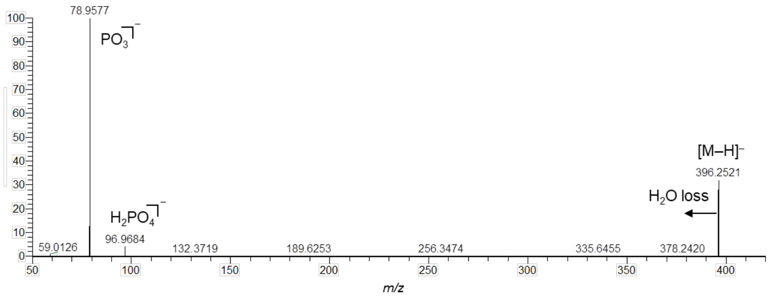

3-keto-SPB d12:1

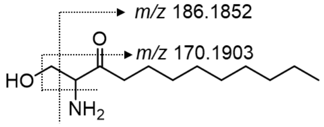

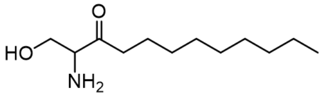

ESI(+)-MS/MS

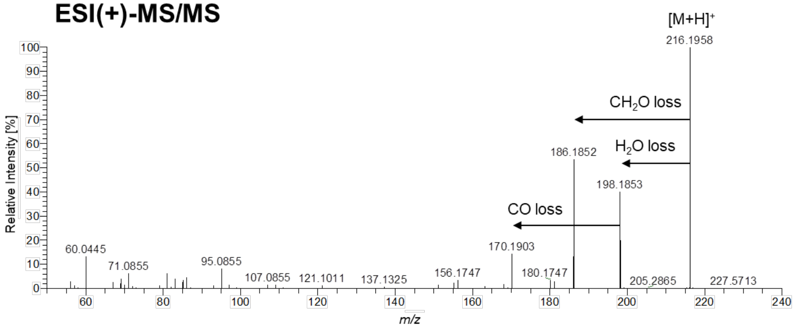

ESI(–)-MS/MS

SPB d18:2

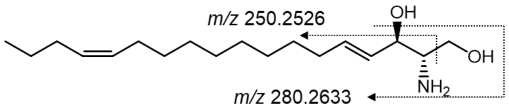

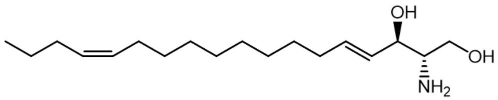

ESI(+)-MS/MS

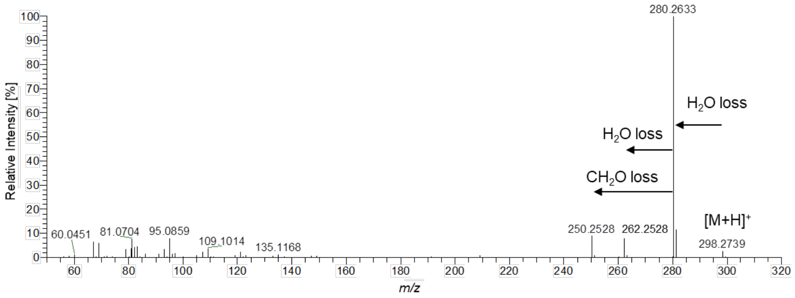

ESI(–)-MS/MS

Cer d18:1/18:0(2OH)

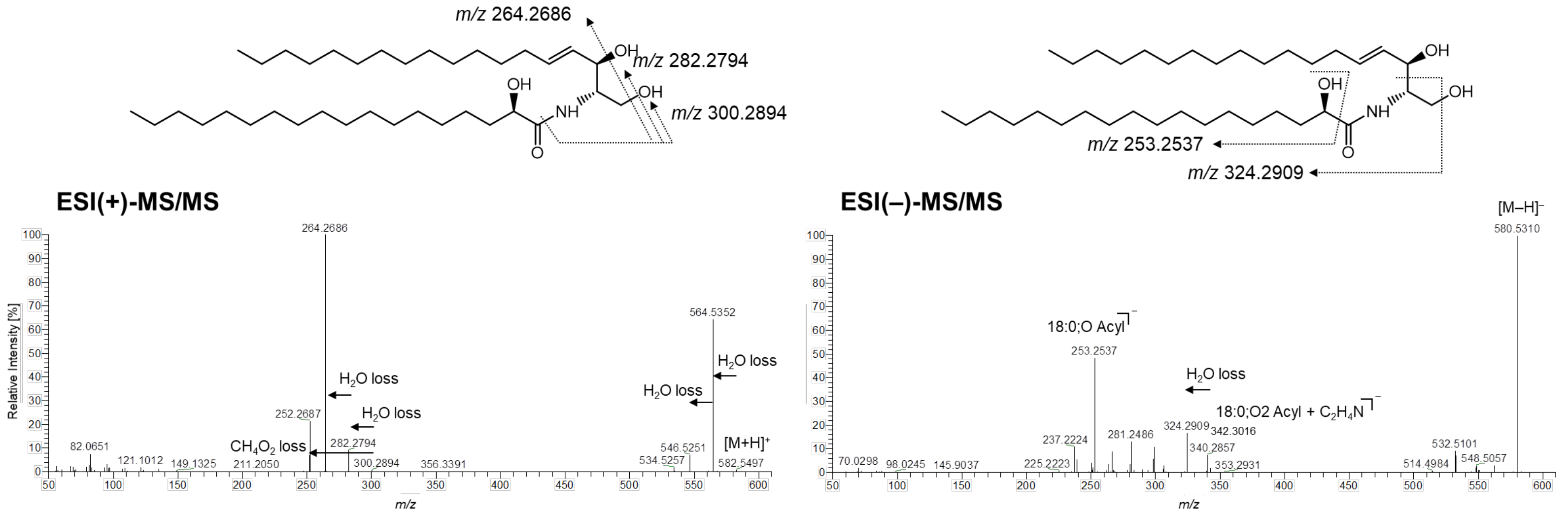

Cer d18:1/16:0

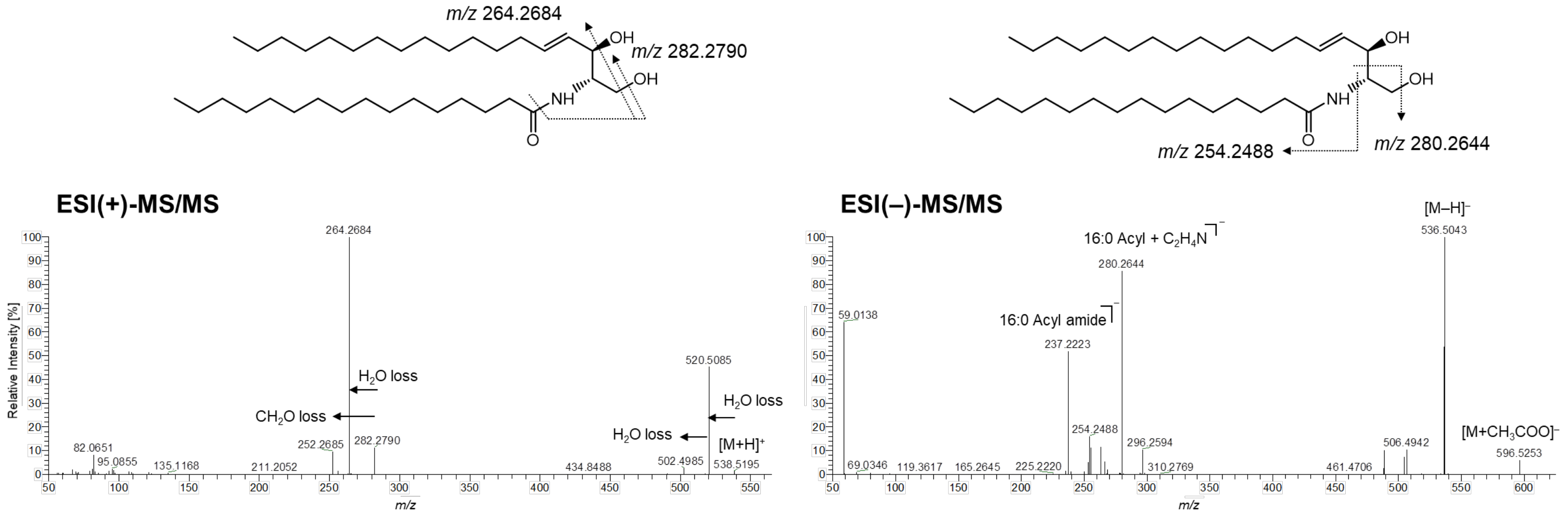

[d_9] Cer d18:0/16:0

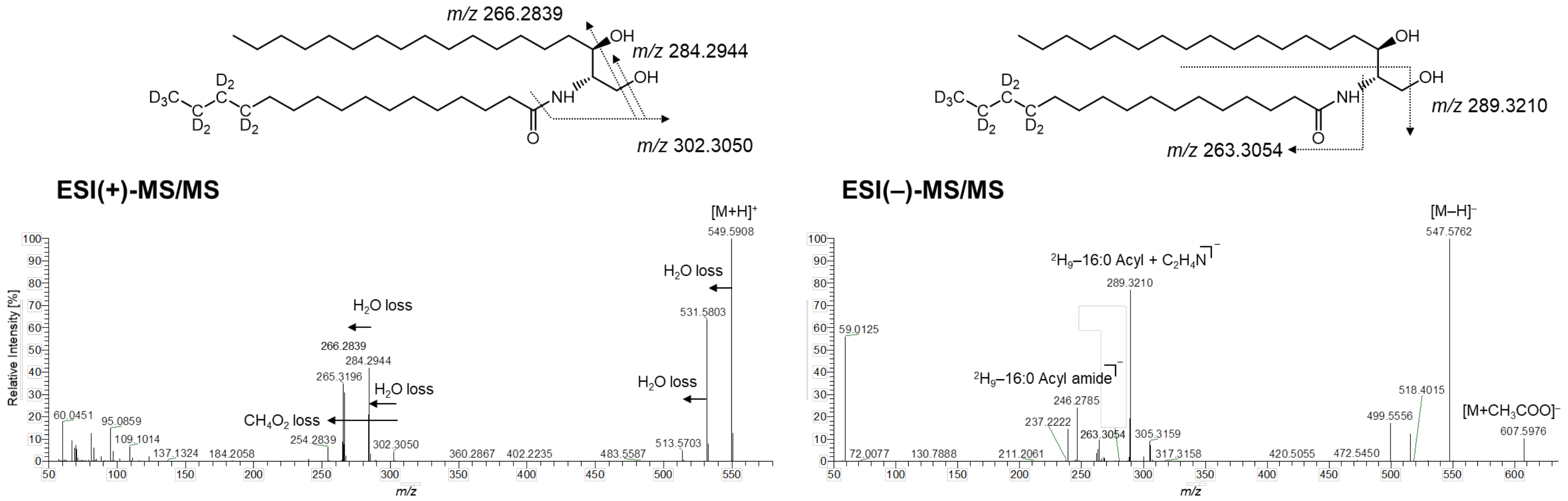

Cer t18:0/24:0

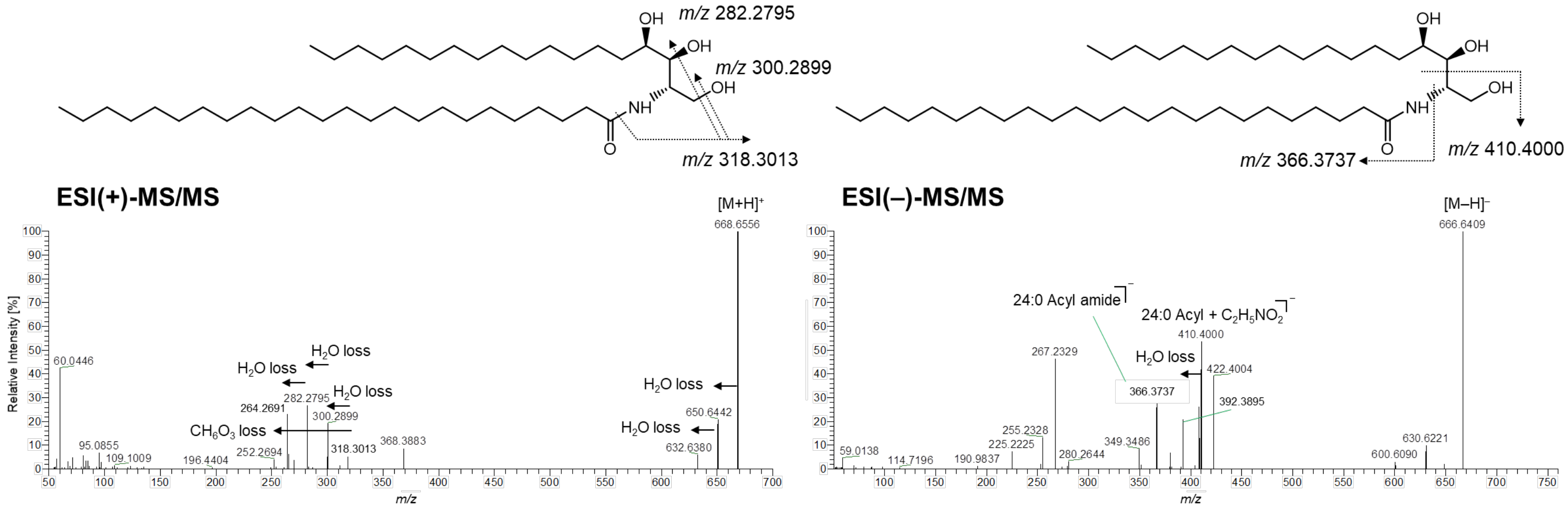

[d_9] Cer t18:0/16:0(2OH)

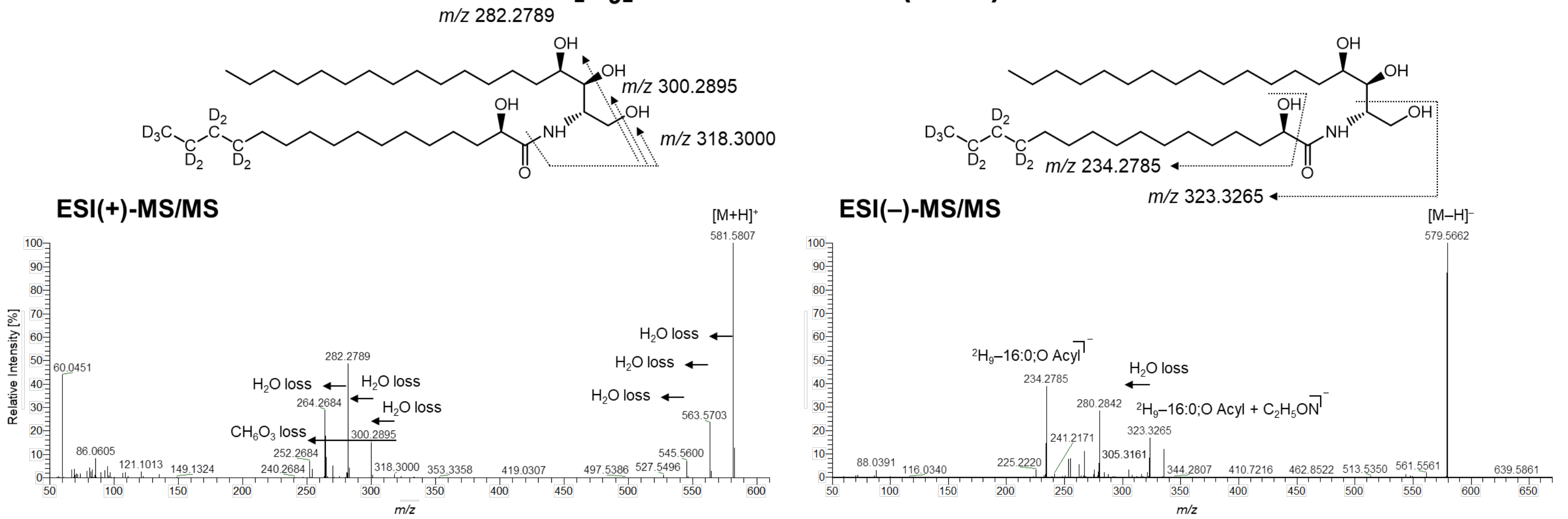

Cer t18:0/26:0/26-O-18:1

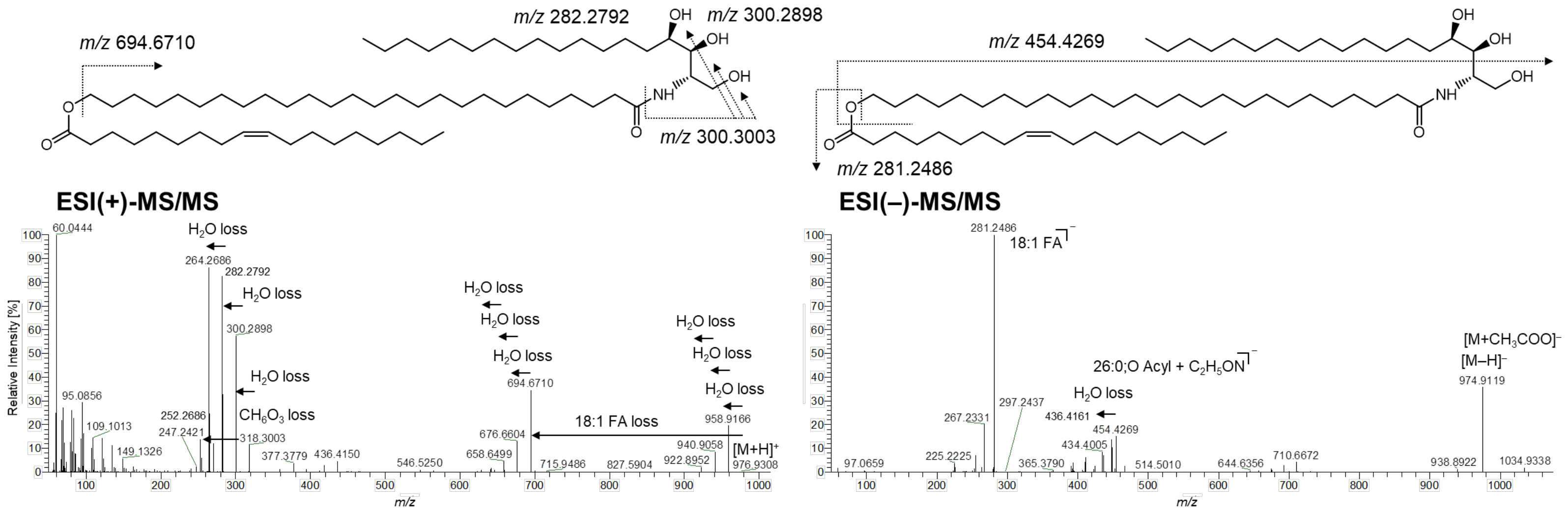

[d_9] Cer d18:1/26:0/26-O-18:1

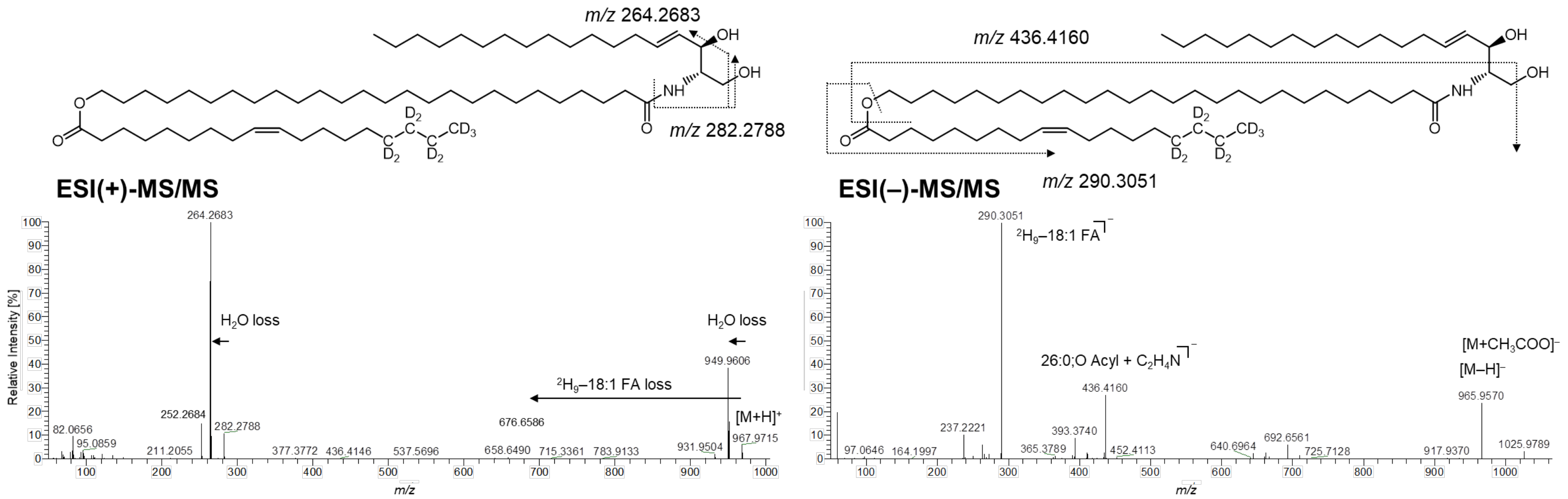

ACer (18:1) d18:1/17:0

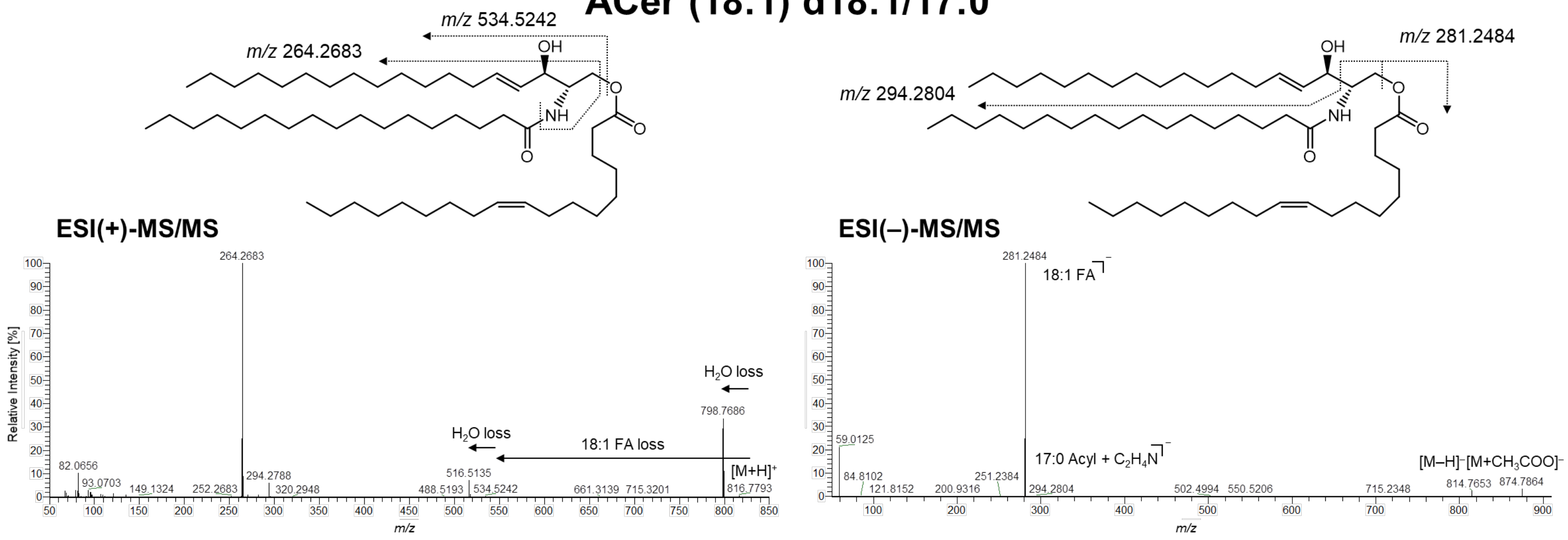

CerP d18:1/16:0

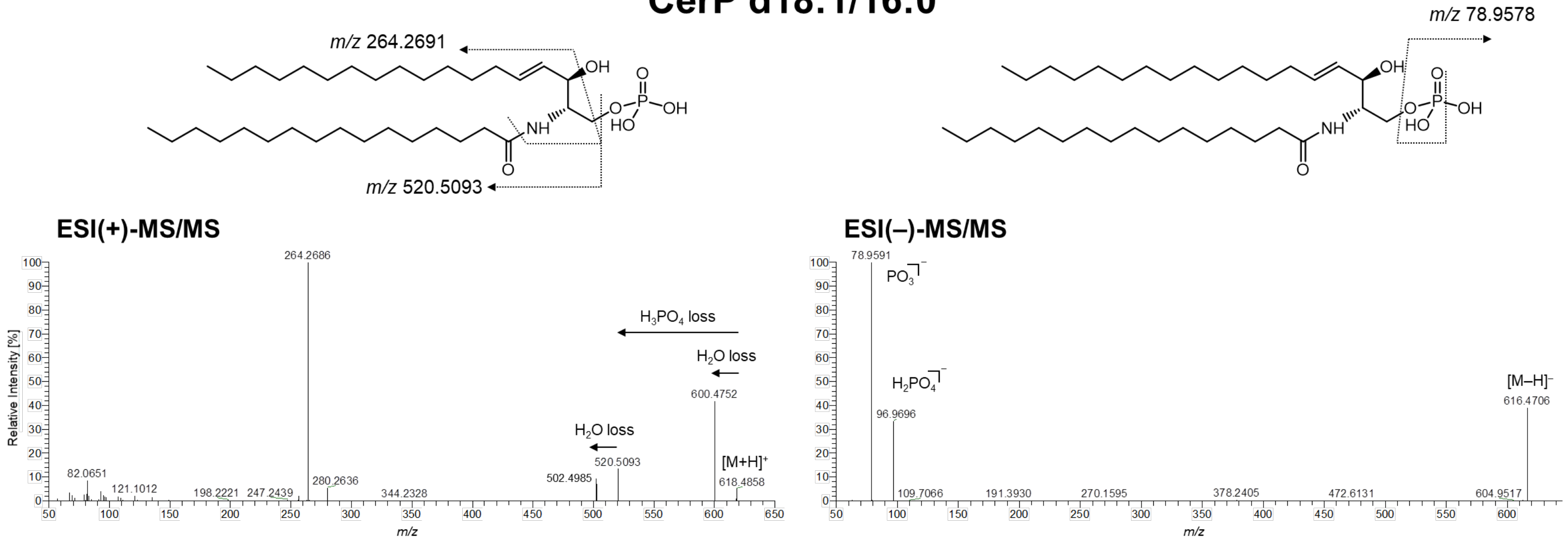

PE-SPB d18:1

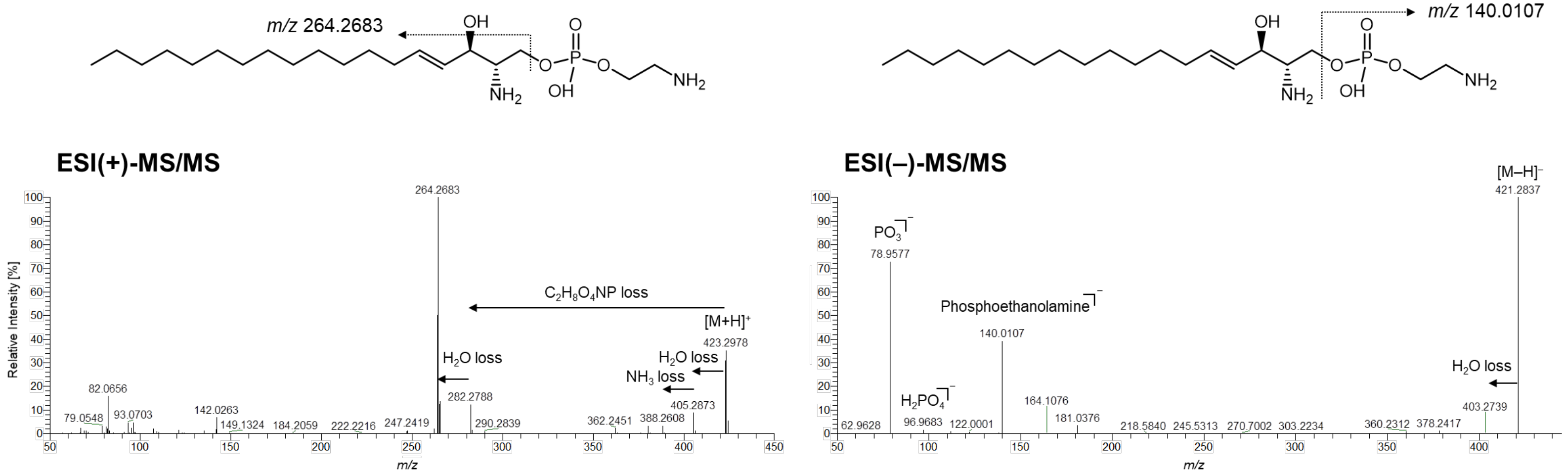

PI-SPB d18:1

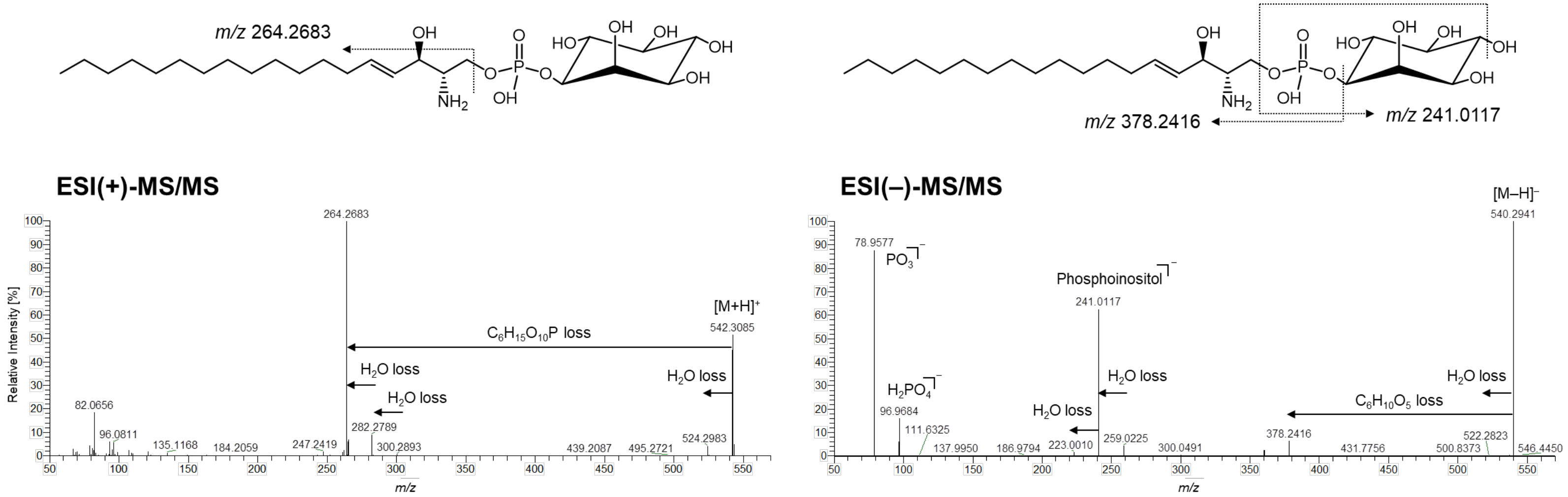

SM d18:1/18:0

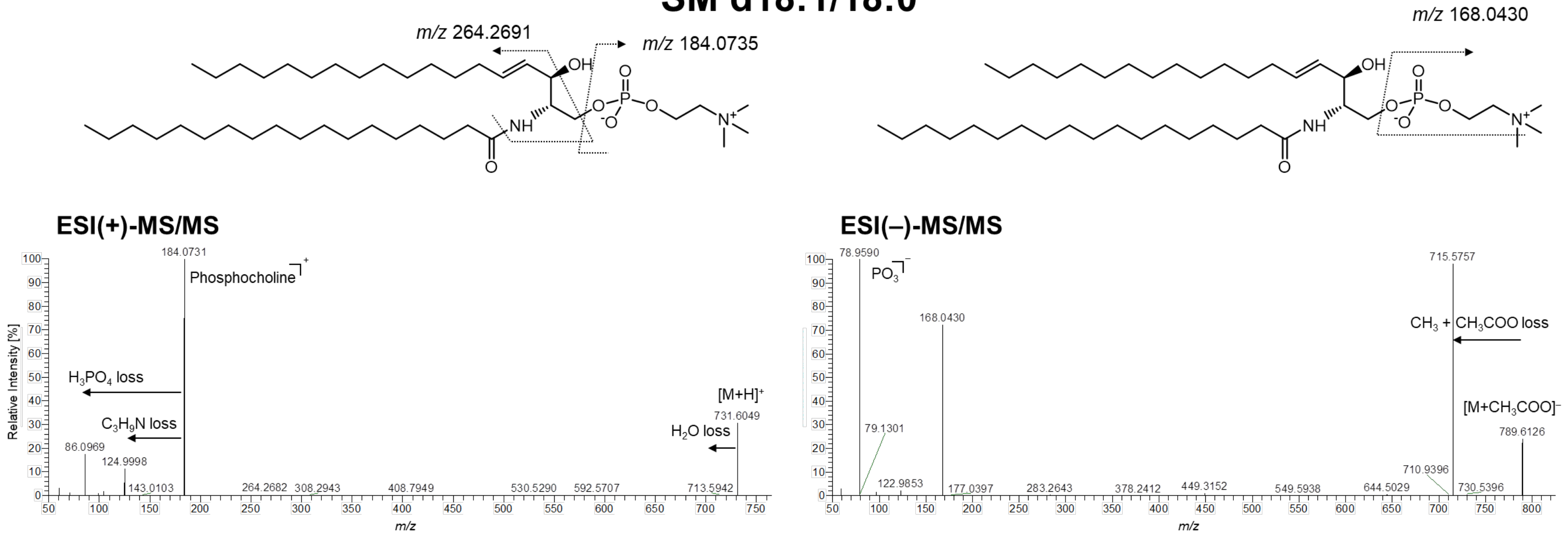

[d_9] LSM d18:1

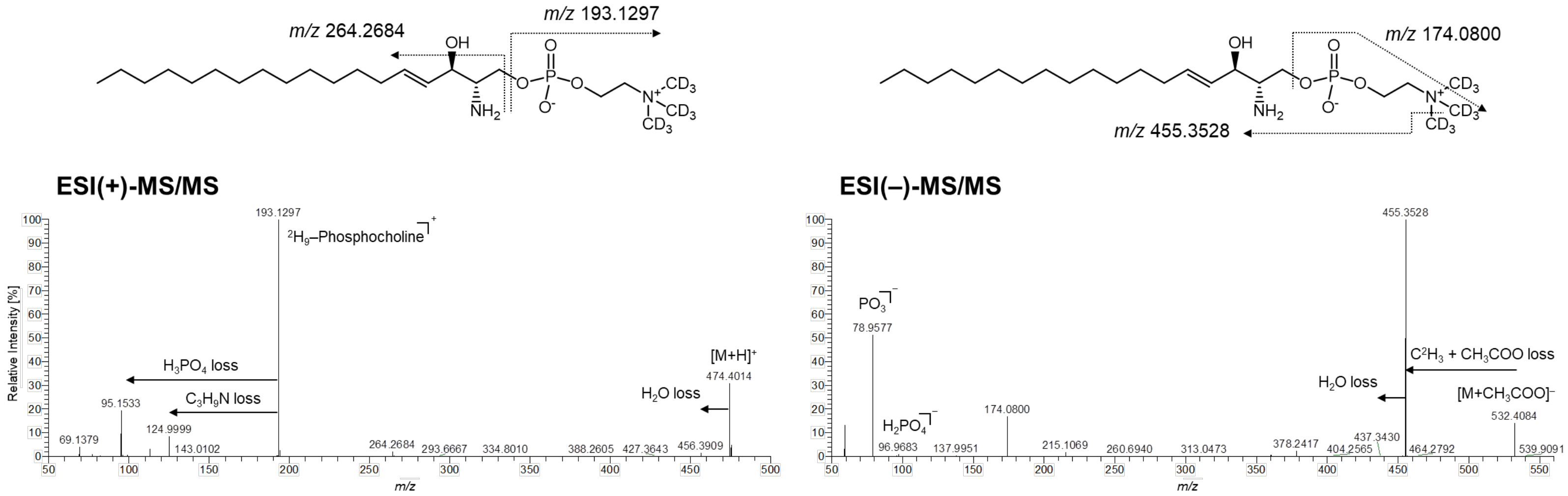

PE-Cer d17:1/12:0

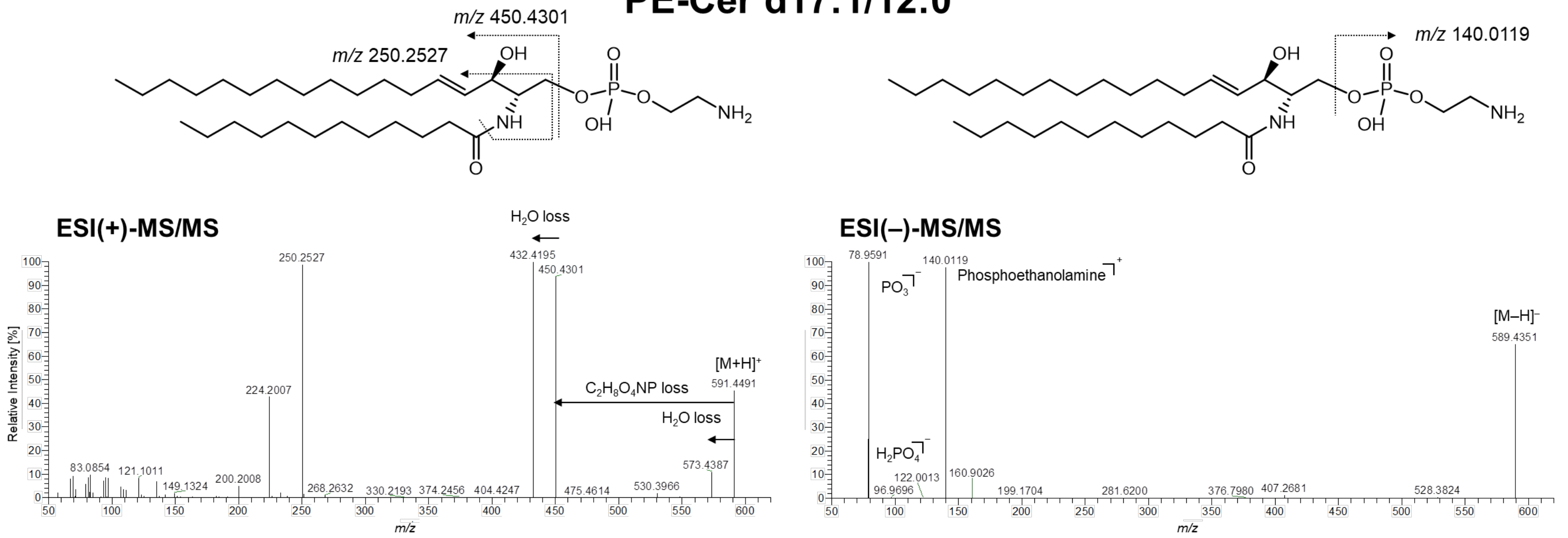

$[d_5]$ Hex-SPB d18:1

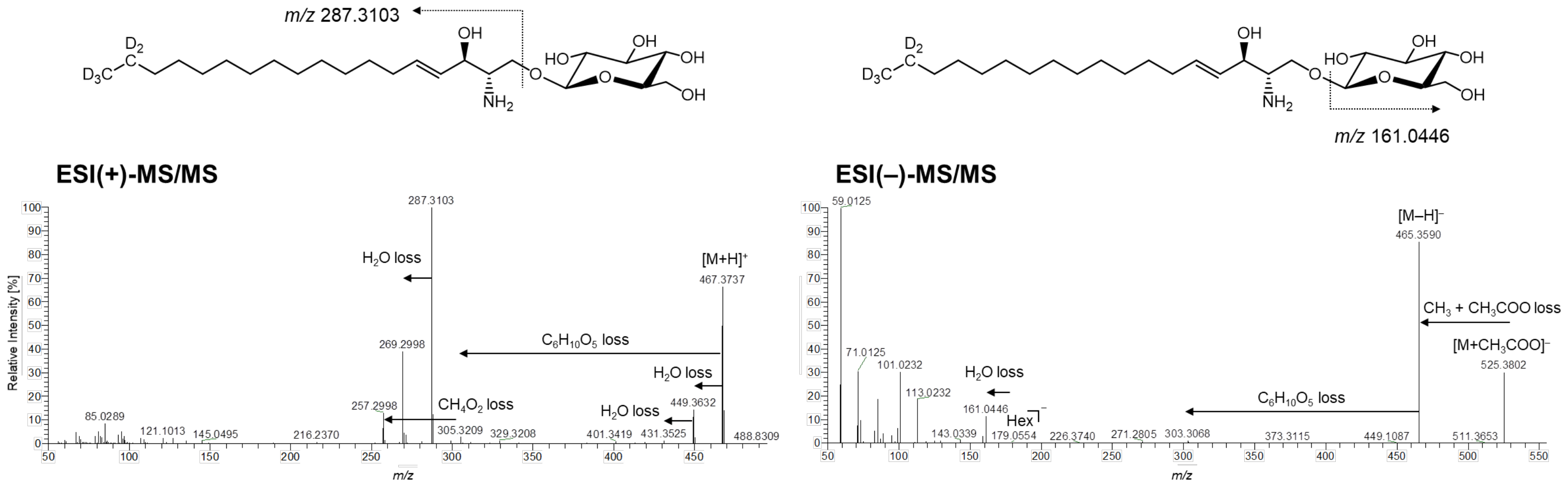

HexCer d18:0/22:0

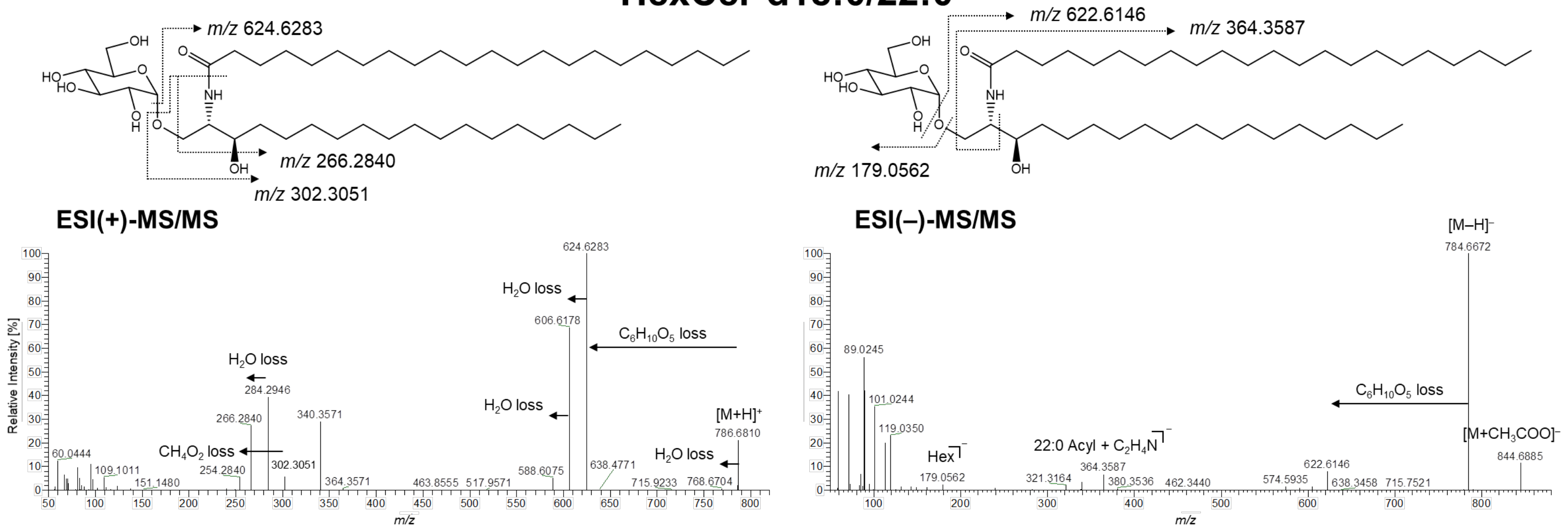

HexCer d18:1/16:0

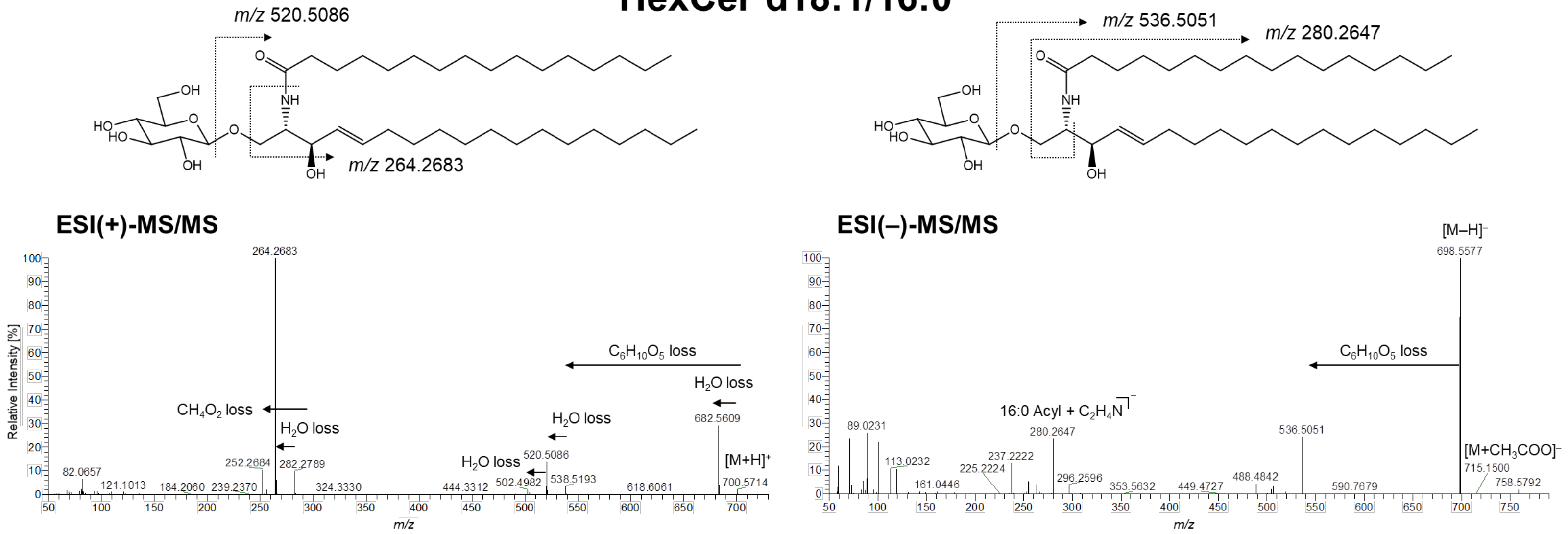

HexCer t18:0/26:0

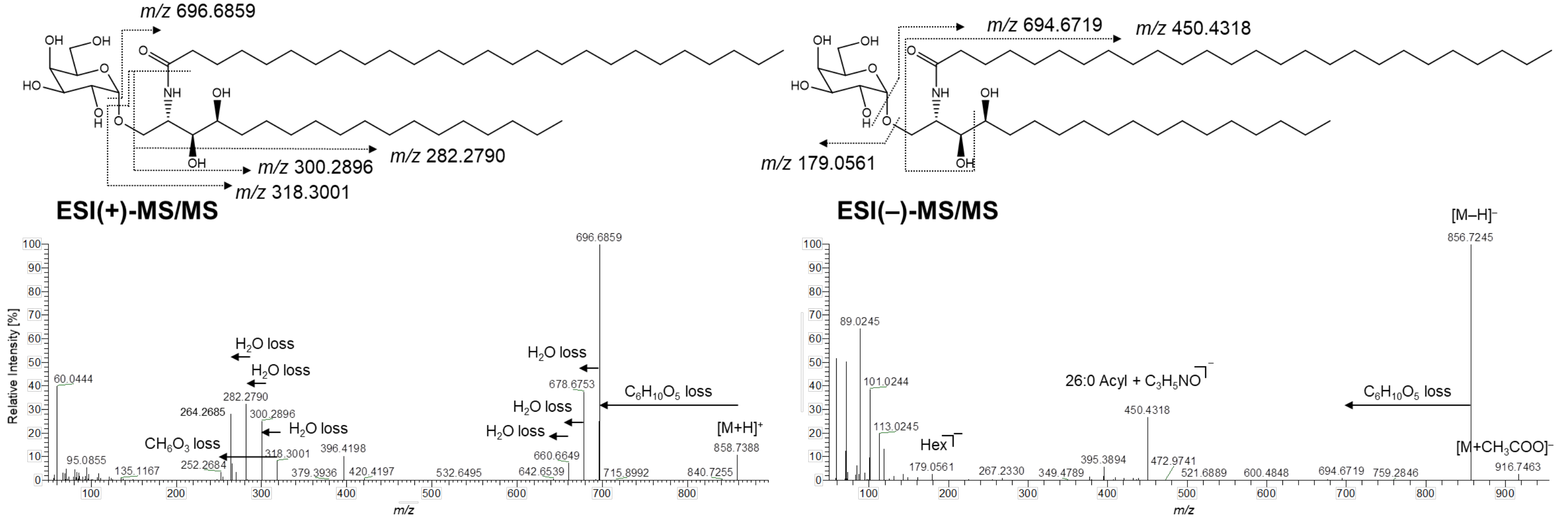

GB3 d18:1/17:0 (Hex3Cer d18:1/17:0)

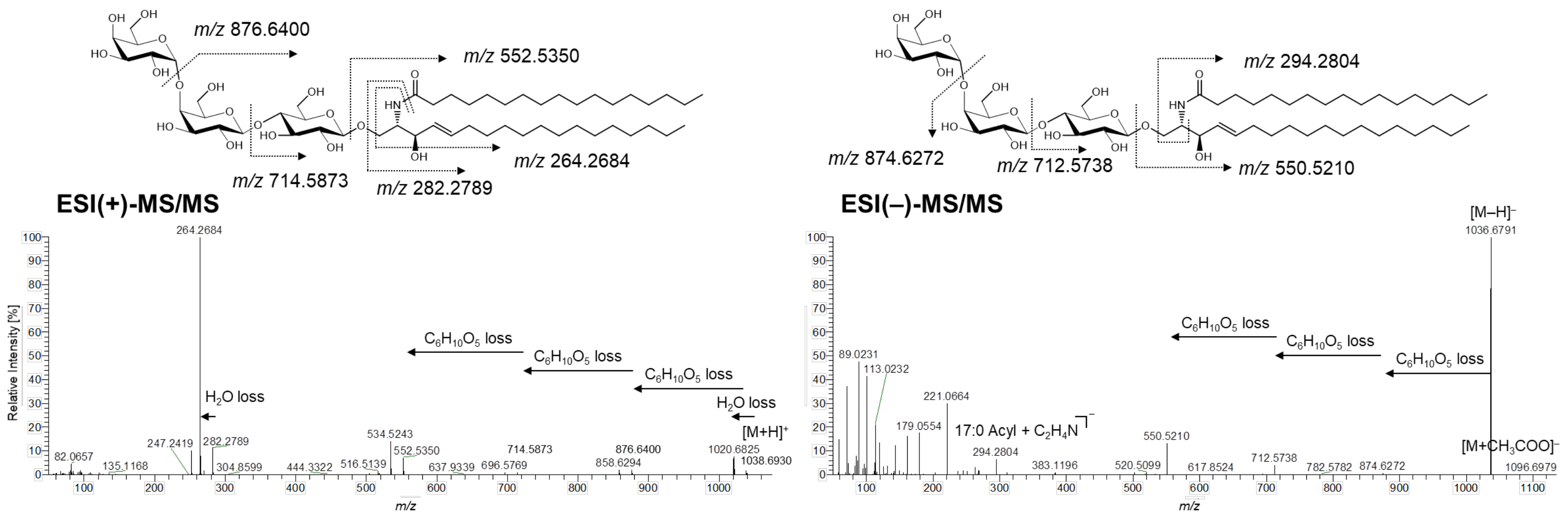

GA2 d18:1/17:0 (Hex2Cer d18:1/17:0)

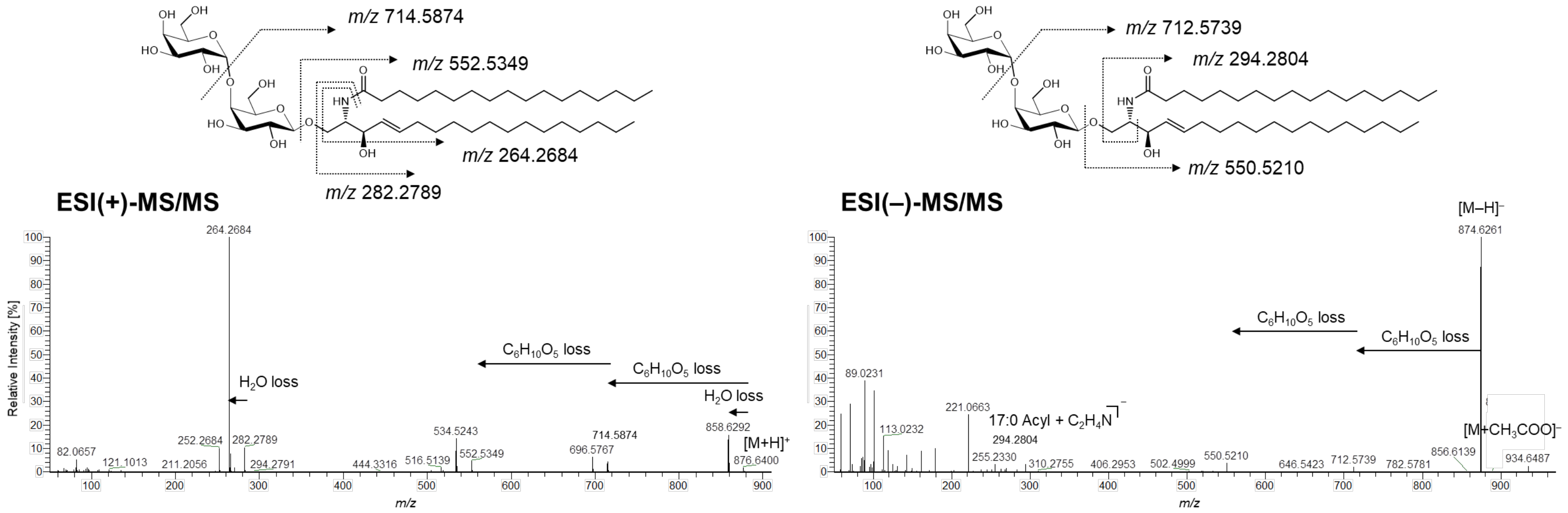

[d_5] GM3 d18:1/18:0

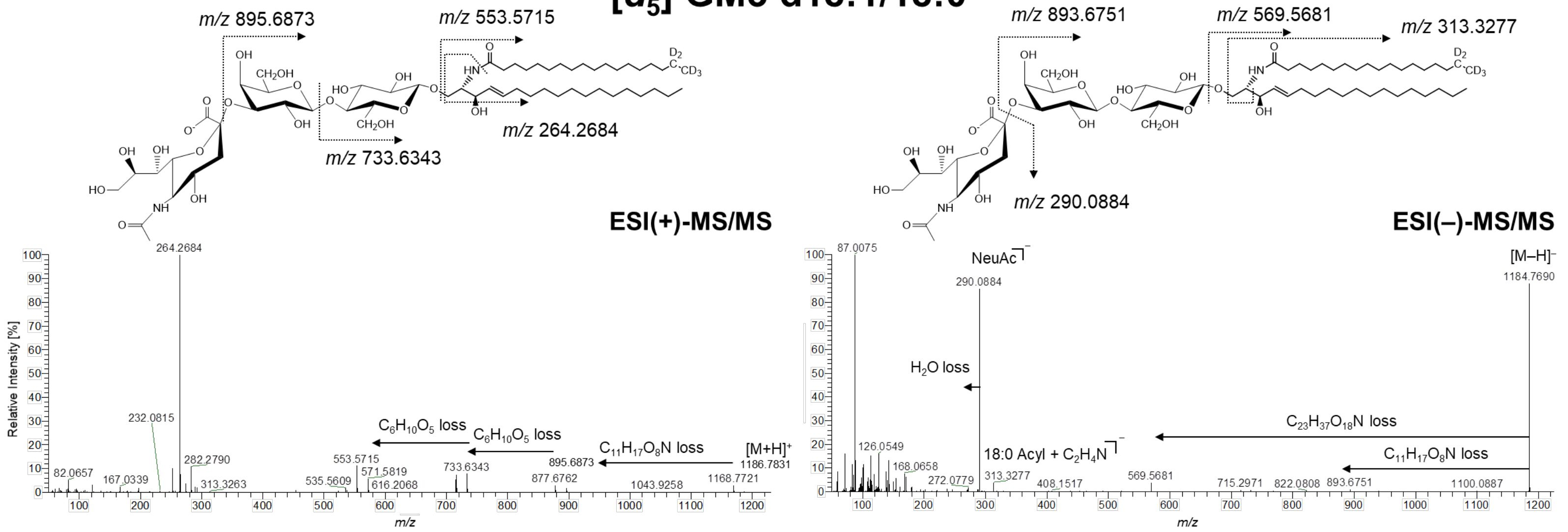

[d_5] GM1 d18:1/18:0

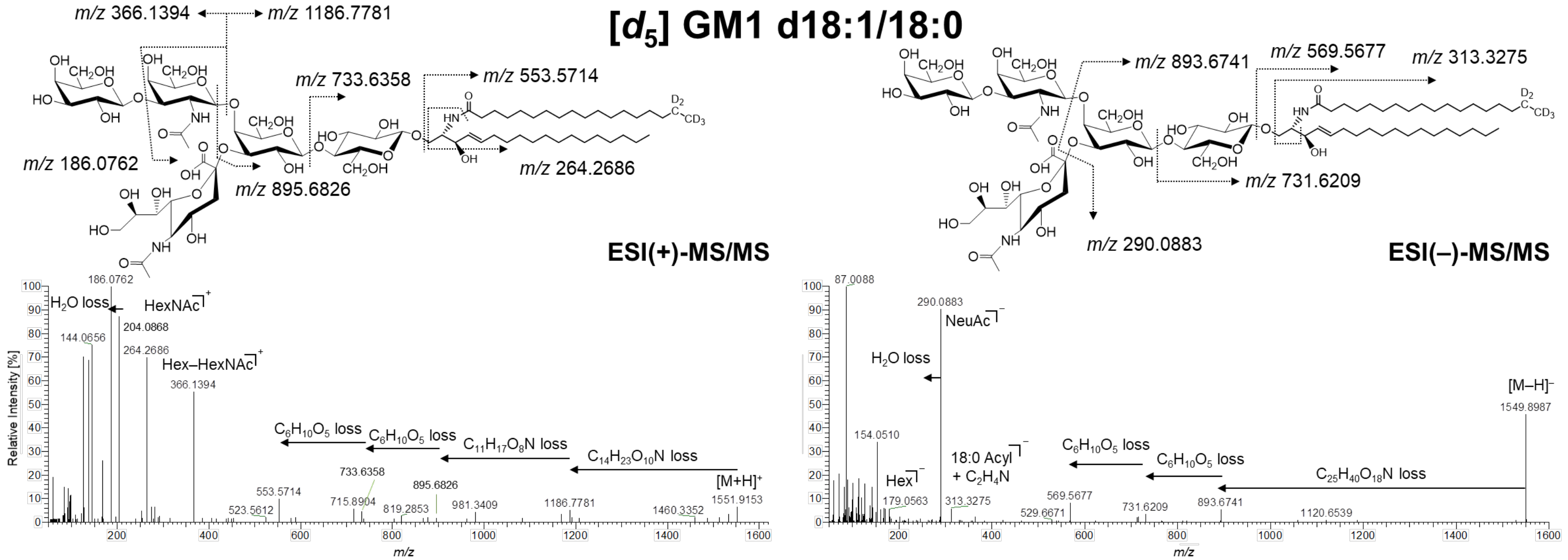

SHexCer d18:1/18:0(OH)

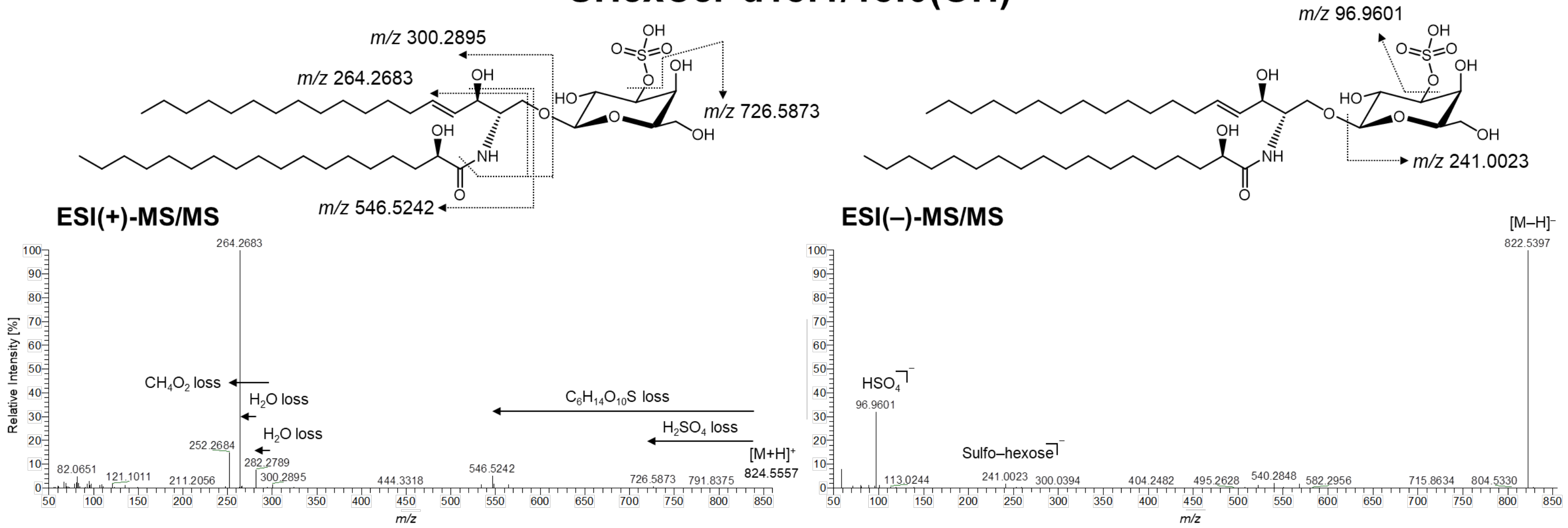

[d_7] SHexCer d18:1/13:0

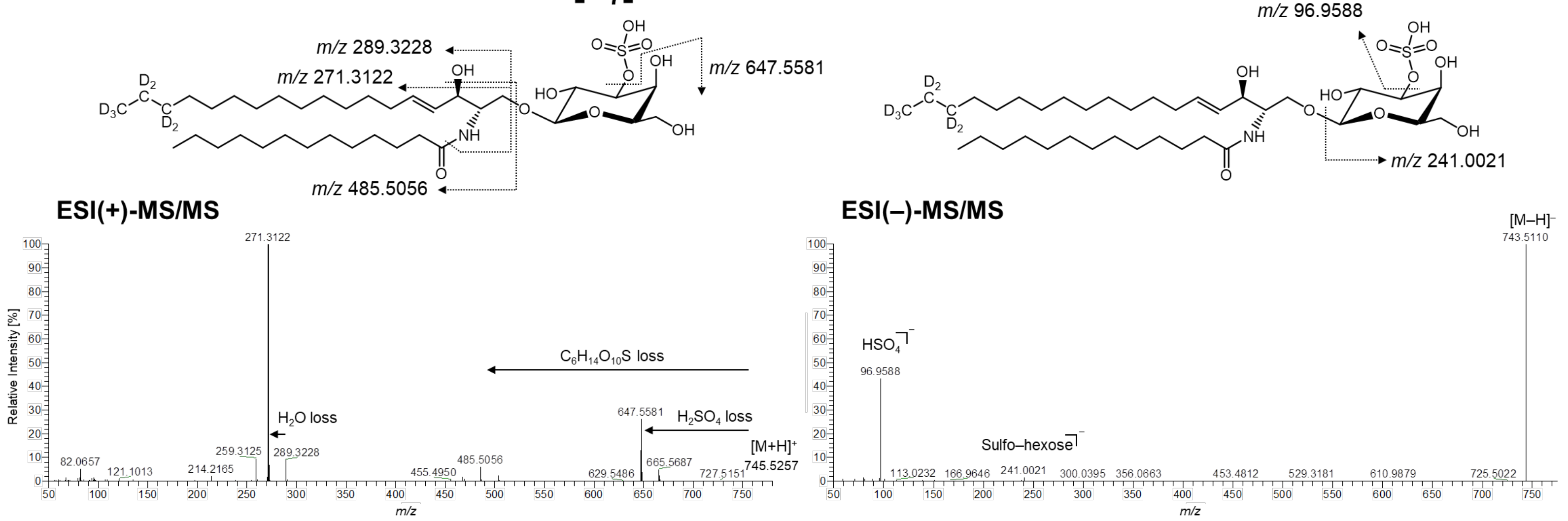

ST 27:1;O

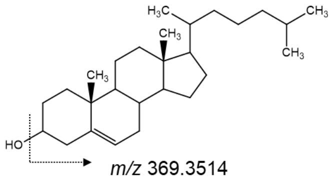

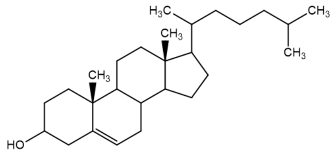

ESI(+)-MS/MS

ESI(–)-MS/MS

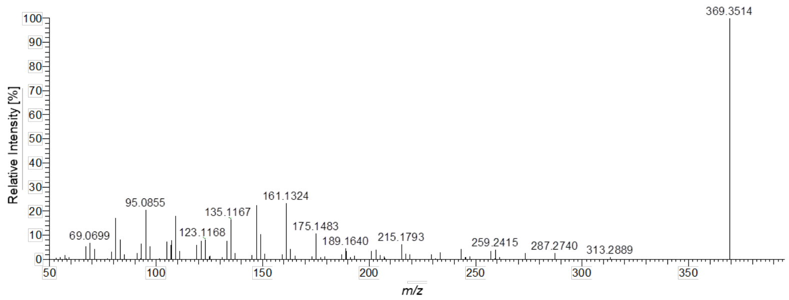

CE 16:0

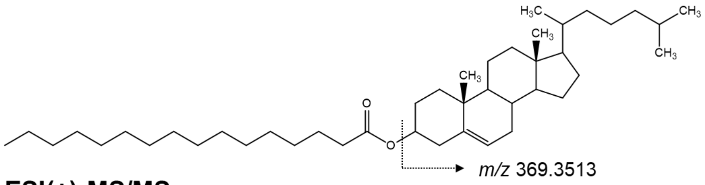

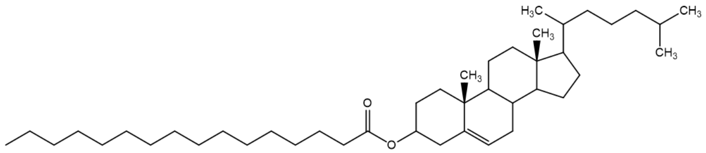

ESI(+)-MS/MS

ESI(–)-MS/MS

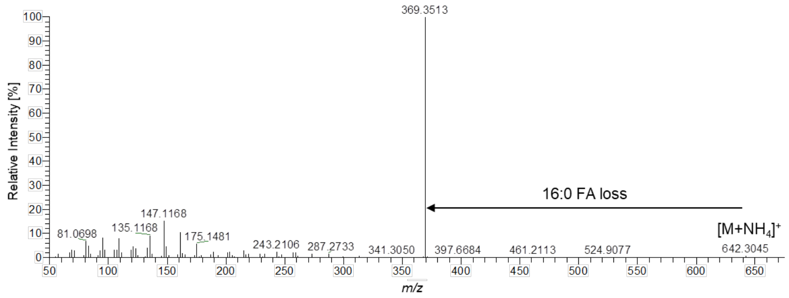

ST 27:1;O;Hex;FA 14:0

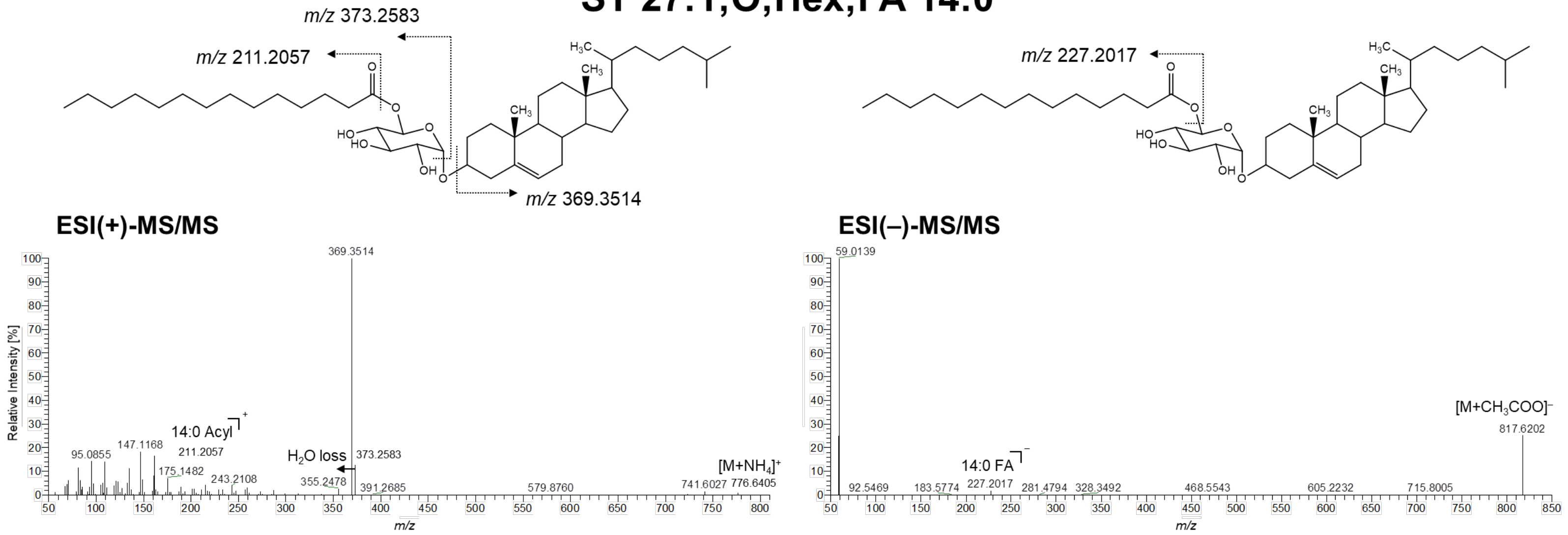

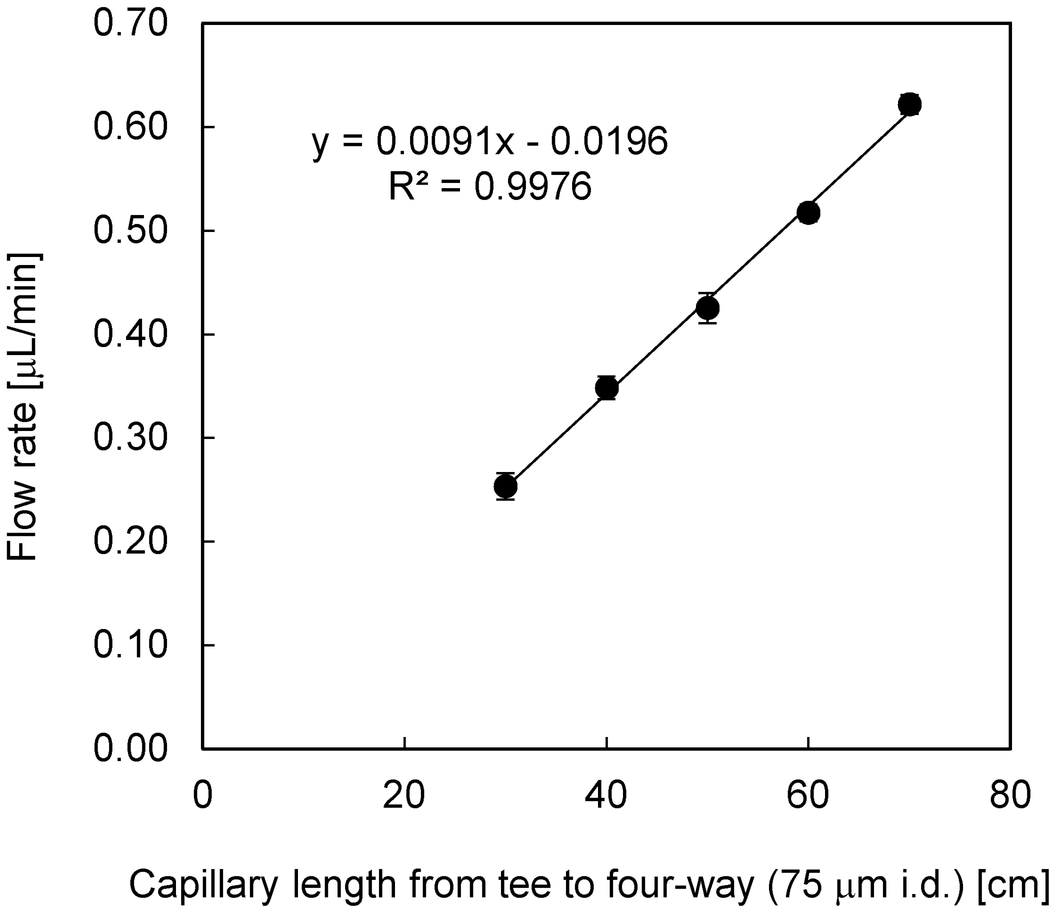

Supplementary Fig. 4 Relationship between the length of capillary tubing used in the flow path from the T-piece to the four-way valve and the actual flow velocity (mean ± standard deviation, $n = 3$).

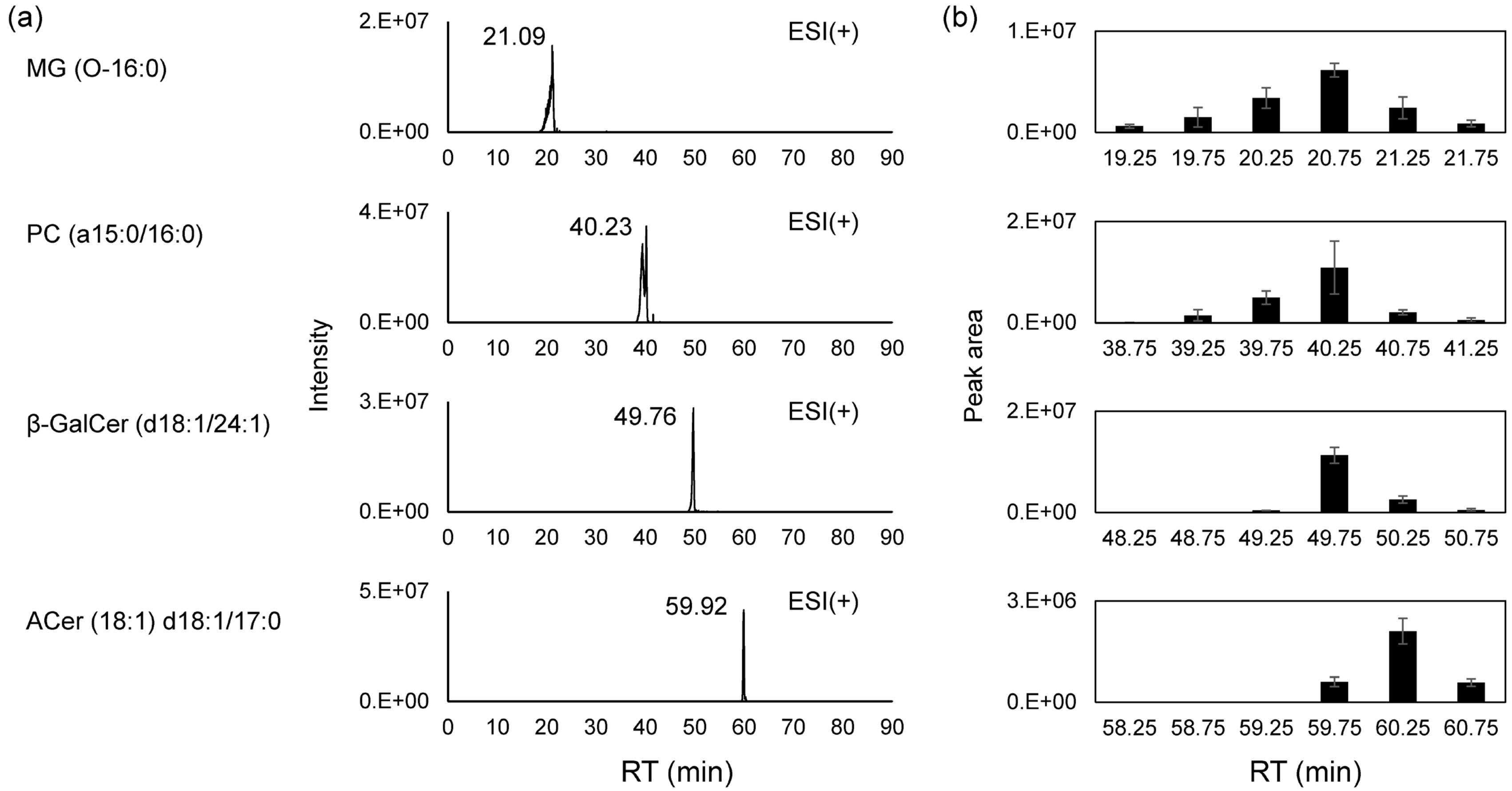

Supplementary Fig. 5 RT and fractionation patterns of each lipid subclass standard. (a) LC-FRC-HRMS chromatogram of each lipid subclass standard. Polarity and RT were described in the chromatogram. (b) Peak area values of the fractionated samples obtained by FI-HRMS analysis (mean ± standard deviation, *n* = 3).
