# Supplementary material for: Efficient lipidomic approach for the discovery of lipid ligands for immune receptors by combining LC-HRMS/MS analysis with fractionation and reporter cell assay
Source: Anal Bioanal Chem. 2023 Dec 23;416(25):5445–56. doi: 10.1007/s00216-023-05111-w (PMC11427514; doi:10.1007/s00216-023-05111-w)
Supplement: Supplementary file 2 — Supplementary file2 (DOCX 171 KB) [file 216_2023_5111_MOESM2_ESM.docx]

**Supplementary Table 1**LIPID MAPS ID, name, abbreviation, standard information, and observed adduct ions for each lipid subclass used in this study

| LIPID MAPS ID^a^ | Lipid subclass | | Lipid species | Formula | Exact mass | Adduct ions in positive ion mode | | | Adduct ions in negative ion mode | | |
| --- | --- | --- | --- | --- | --- | --- | --- | --- | --- | --- | --- |
|  | Name | Abbreviation |  |  |  | [M+H]^+^ | [M+2H]^2+^ | [M+NH_4_]^+^ | [M‒H]^‒^ | [M‒2H]^2‒^ | [M+Ac]^‒^ |
| FA0101 | Straight chain fatty acid | FA | FA 32:6 | C_32_H_52_O_2_ | 468.3967 |  |  | + | ++ |  |  |
| FA0102 | Branched fatty acid | aFA | aFA 15:0 | C_15_H_30_O_2_ | 242.2246 |  |  |  | ++ |  | ++ |
| FA0105 | Hydroxy fatty acid | HFA | HFA 18:1 | C_18_H_34_O_3_ | 298.2508 |  |  |  | ++ |  |  |
| FA0116 | α-Mycolic acid | α-MA | FA80:2;O | C_80_H_156_O_3_ | 1165.2054 | + |  | ++ | ++ |  |  |
| FA0116 | keto-Mycolic acid | keto-MA | FA 86:2;O2 | C_86_H_168_O_4_ | 1265.2943 | + |  | ++ | ++ |  |  |
| FA0116 | methoxy-Mycolic acid | methoxy-MA | FA 85:1;O2 | C_85_H_168_O_4_ | 1253.2943 | ++ |  | + | ++ |  |  |
| FA0600 | Fatty aldehyde | Fatty aldehydes | [*d*_9_] Fattty aldehyde 16:0 | C_16_H_23_D_9_O | 249.3018 | ++ |  |  |  |  |  |
| FA0707 | Fatty acyl carnitine | CAR | CAR 16:0 | C_23_H_45_NO_4_ | 399.3349 | ++ |  |  | ++ |  | ++ |
| FA0709 | Fatty acid estolide | FAHFA | FAHFA 18:0/9-O-16:0 | C_34_H_66_O_4_ | 538.4961 | ++ |  | ++ | ++ |  | ++ |
| FA0802 | *N*-acyl amine (glycine) | NAGly | NAGly 18:1 | C_20_H_37_NO_3_ | 339.2773 | ++ |  | + | ++ |  |  |
| FA0802 | *N*-acyl amine (homocysteine) | NAHC | NAHC 16:0 | C_20_H_39_NO_3_S | 373.2651 | ++ |  |  |  |  |  |
| FA0802 | *N*-acyl amine (taurine) | NATau | NATau 16:0 | C_18_H_37_NO_4_S | 363.2443 | ++ |  | ++ | ++ |  |  |
| GL0101 | Monoacylglycerol | MG | MG 16:0 | C_19_H_38_O_4_ | 330.2770 | + |  | ++ |  |  | ++ |
| GL0102 | Monoalkylglycerol | Ether MG | Ether MG O-16:0 | C_19_H_40_O_3_ | 316.2977 | + |  | ++ |  |  | ++ |
| GL0103 | Mono-(1Z-alkenyl)-glycerol | Ether MG (P) | Ether MG P-18:0 | C_21_H_42_O_3_ | 342.3134 | + |  | + |  |  | ++ |
| GL0201 | Diacylglycerol | DG | DG 18:0/20:4 | C_41_H_72_O_5_ | 644.5380 | + |  | ++ |  |  | ++ |
| GL0301 | Triacylglycerol | TG | TG 18:0/18:0/18:0 | C_57_H_110_O_6_ | 890.8302 |  |  | ++ |  |  |  |
| GP0101 | Diacylglycerophosphocholine | PC | PC 16:0/18:1 | C_42_H_82_NO_8_P | 759.5778 | ++ |  |  |  |  | ++ |
| GP0102 | 1-alkyl,2-acylglycerophosphocholine | EtherPC | PC O-16:0/20:4 | C_44_H_82_NO_7_P | 767.5829 | ++ |  |  |  |  | ++ |
| GP0103 | 1-(1Z-alkenyl),2-acylglycerophosphocholine | EtherPC (P) | PC P-18:0/20:4 | C_46_H_84_NO_7_P | 793.5985 | ++ |  |  |  |  | ++ |
| GP0105 | Monoacylglycerophosphocholine | LPC | LPC 18:0 | C_26_H_54_NO_7_P | 523.3638 | ++ |  |  |  |  | ++ |
| GP0106 | Monoalkylglycerophosphocholine | EtherLPC | LPC O-18:0 | C_26_H_56_NO_6_P | 509.3845 | ++ |  |  |  |  | ++ |
| GP0107 | 1Z-alkenylglycerophosphocholine | EtherLPC (P) | LPC P-18:0 | C_26_H_54_O_6_NP | 507.3689 | ++ |  |  |  |  | ++ |
| GP2001 | Oxidized glycerophosphocholine | OxPC | OxPC 16:0/9:0;O | C_33_H_64_NO_9_P | 649.4319 |  |  |  |  |  | + |
| GP2001 | Oxidized glycerophosphocholine | OxPC | OxPC 16:0/4:0;O2 | C_28_H_54_NO_10_P | 595.3485 | ++ |  | + | ++ |  |  |
| GP0201 | Diacylglycerophosphoethanolamine | PE | PE 18:0/20:4 | C_43_H_78_NO_8_P | 767.5465 | ++ |  |  | ++ |  |  |
| GP2103 | 1-(1Z-alkenyl),2-acylglycerophosphoethanolamine glycan | EtherPE (P) | PE P-18:0/20:4 | C_43_H_78_NO_7_P | 751.5516 | ++ |  |  | ++ |  |  |
| GP0201 | Diacylglycerophosphoethanolamine | MPE | MPE 16:0/16:0 | C_38_H_76_NO_8_P | 705.5309 | ++ |  |  | ++ |  |  |
| GP0205 | Monoacylglycerophosphoethanolamine | LPE | LPE 18:0 | C_23_H_48_NO_7_P | 481.3168 | ++ |  |  | ++ |  |  |
| GP0207 | 1Z-alkenylglycerophosphoethanolamine | EtherLPE (P) | LPE P-18:0 | C_23_H_48_O_6_NP | 465.3219 | ++ |  |  | ++ |  |  |
| GP | Diacylglycerophosphoethanolamine-*N*-lactosyl | Lac-PE | Lac-PE 18:1 | C_53_H_100_NO_18_P | 1069.6678 | ++ |  |  | ++ |  |  |
| GP | Diacylglycerophosphoethanolamine-*N*-succinyl | Suc-PE | Suc-PE 18:1/18:1 | C_45_H_82_NO_11_P | 843.5625 | ++ |  | ++ | ++ |  |  |
| GP0301 | Diacylglycerophosphoserine | PS | PS 18:0/18:1 | C_42_H_80_NO_10_P | 789.5520 | ++ |  |  | ++ |  |  |
| GP0301 | *N*-acyl-phosphatidylserine | NAPS | NAPS 18:1/18:1/19:0 | C_61_H_114_NO_11_P | 1067.8130 | ++ |  | ++ | ++ |  |  |
| GP0305 | Monoacylglycerophosphoserine | LPS | LPS 16:0 | C_22_H_44_NPO_9_ | 497.2754 | ++ |  |  | ++ |  |  |
| GP0401 | Diacylglycerophosphoglycerol | PG | PG 16:0/18:1 | C_40_H_77_O_10_P | 748.5254 | ++ |  | ++ | ++ |  |  |
| GP | Diacylglycerophospholysylglycerol | Lysyl-PG | Lysyl-PG 16:0/16:0 | C_44_H_87_N_2_O_11_P | 850.6047 | ++ |  |  | ++ |  | + |
| GP0405 | Monoacylglycerophosphoglycerol | LPG | LPG 18:0 | C_24_H_49_O_9_P | 512.3114 | ++ |  | ++ | ++ |  |  |
| GP0409 | Diacylglycerophosphomonoradylglycerol | SLBPA | SLBPA 18:1/18:1/16:0 | C_58_H_109_O_11_P | 1012.7708 | ++ |  | ++ | ++ |  |  |
| GP0410 | Monoacylglycerophosphomonoradylglycerol | LBPA | LBPA 18:1/18:1 | C_42_H_79_O_10_P | 774.5411 | ++ |  | ++ | ++ |  |  |
| GP0408 | Diacylglycerophosphodiradylglycerol | BPA | BPA 18:1/18:1/18:1/17:0 | C_77_H_143_O_12_P | 1291.0317 | + |  | ++ | ++ |  |  |
| GP0601 | Diacylglycerophosphoinositol | PI | PI 18:0/20:4 | C_47_H_83_O_13_P | 886.5571 | + |  | ++ | ++ |  | ++ |
| GP0605 | Monoacylglycerophosphoinositol | LPI | LPI 18:0 | C_27_H_53_O_12_P | 600.3275 | ++ |  | ++ | ++ |  |  |
| GP0603 | 1-(1Z-alkenyl),2-acylglycerophosphoinositol | PIP | PIP 18:1/18:1 | C_45_H_84_O_16_P_2_ | 942.5235 | ++ |  | ++ | ++ | ++ |  |
| GP0801 | Diacylglycerophosphoinositol bisphosphate | PIP2 | PIP2 18:1/18:1 | C_45_H_85_O_19_P_3_ | 1022.4898 | + |  | + | + | ++ |  |
| GP0901 | Diacylglycerophosphoinositol trisphosphate | PIP3 | PIP3 18:1/18:1 | C_45_H_86_O_22_P_4_ | 1102.4561 | ++ |  | ++ | ++ | ++ |  |
| GP1001 | Diacylglycerophosphate | PA | PA 16:0/18:1 | C_37_H_71_O_8_P | 674.4887 | + |  | ++ | ++ |  | + |
| GP1005 | Monoacylglycerophosphate | LPA | LPA 18:0 | C_21_H_43_O_7_P | 438.2746 |  |  | ++ | ++ |  |  |
| GP1101 | Diacylglyceropyrophosphate | DGPP | DGPP 18:1/18:1 | C_39_H_74_O_11_P_2_ | 780.4706 |  |  | ++ | ++ |  |  |
| GP1201 | Diacylglycerophosphoglycerophosphodiradylglycerol | CL | [*d*_5_] CL 18:2/18:2 | C_81_H_137_D_5_O_17_P_2_ | 1454.0036 |  |  | + | ++ | + |  |
| GP1301 | CDP-diacylglycerol | CDP-DG | CDP-DG 16:0/16:0 | C_44_H_81_N_3_O_15_P_2_ | 953.5143 | ++ |  |  | ++ |  |  |
| GP2501 | Monoacyl cyclic glycerophosphatidic acid | CPA | CPA 17:0 | C_20_H_42_NO_6_P | 406.2484 | ++ |  |  |  |  |  |
| GP | Diacylglycerophosphothioethanol | PTE | PTE 16:0/16:0 | C_37_H_73_O_8_PS | 708.4764 | + |  | ++ | ++ |  |  |
| SP0101 | Sphing-4-enine (Sphingosine) | Sph | [*d*_7_] SPB d18:1 | C_18_H_30_D_7_NO_2_ | 306.3264 | ++ |  |  |  |  | ++ |
| SP0102 | Sphinganine | DHSph | [*d*_7_] SPB d18:0 | C_18_H_32_D_7_NO_2_ | 308.3420 | ++ |  |  |  |  | ++ |
| SP0105 | Sphingoid base 1-phosphate | Sph-P | [*d*_7_] SPBP d18:1 | C_18_H_31_D_7_NO_5_P | 386.2927 | ++ |  |  | ++ |  |  |
| SP0105 | Dihydrosphingoid base 1-phosphate | DHSph-P | [*d*_7_] SPBP d18:0 | C_18_H_33_D_7_NO_5_P | 388.3083 | ++ |  |  | ++ |  |  |
| SP | Methyl-sphing-4-enine (Methyl-sphingosine) | DOSph | SPB m18:0 | C_18_H_39_NO | 285.3032 | ++ |  |  |  |  |  |
| SP | 1-desoxymethyl-sphing-4-enine (1-desoxymethyl-sphingosine) | - | 1-desoxymethyl SPB m17:0 | C_17_H_37_NO | 271.2875 | ++ |  |  |  |  |  |
| SP0107 | *N*-methylated sphingoid bases | - | *N,N*-Dimethyl-SPB d17:1 | C_19_H_39_NO_2_ | 313.2981 | ++ |  |  |  |  | + |
| SP0105 | Sphingoid base 1-phosphate | - | SPBP t18:0 | C_18_H_40_NO_6_P | 397.2593 | ++ |  |  | ++ |  |  |
| SP0101 | Sphing-4-enine (Sphingosines) | - | 3-keto-SPB d12:0 | C_12_H_25_NO_2_ | 215.1885 | ++ |  |  |  |  | ++ |
| SP0108 | Sphingoid base analog | - | SPB d18:2 | C_18_H_35_NO_2_ | 297.2668 | ++ |  |  |  |  | ++ |
| SP0201 | *N*-acylsphingosine (ceramide) | Cer-AS | Cer d18:1/18:0(2OH) | C_36_H_71_NO_4_ | 581.5383 | ++ |  |  | ++ |  | ++ |
| SP0201 | *N*-acylsphingosine (ceramide) | Cer-NS | Cer d18:1/16:0 | C_34_H_67_NO_3_ | 537.5121 | ++ |  |  | ++ |  | ++ |
| SP0202 | *N*-acylsphinganine (dihydroceramide) | Cer-NDS | [*d*_9_] Cer d18:0/16:0 | C_34_H_60_D_9_NO_3_ | 548.5842 | ++ |  |  | + |  | ++ |
| SP0203 | *N*-acyl-4-hydroxysphinganine (phytoceramide) | Cer-NP | Cer t18:0/24:0 | C_42_H_85_NO_4_ | 667.6479 | ++ |  |  | ++ |  | ++ |
| SP0203 | *N*-acyl-4-hydroxysphinganine (phytoceramide) | Cer-AP | [*d*_9_] Cer t18:0/16:0(2OH) | C_34_H_60_D_9_NO_5_ | 580.5741 | ++ |  |  | + |  | ++ |
| SP0204 | Acylceramide | Cer-EOP | Cer t18:0/26:0/26-O-18:1 | C_62_H_121_NO_6_ | 975.9194 | ++ |  |  | + |  | ++ |
| SP0204 | Acylceramide | Cer-EOS | [*d*_9_] Cer t18:1/26:0/26-O-18:1 | C_62_H_110_D_9_NO_5_ | 966.9653 | ++ |  |  |  |  | ++ |
| SP0204 | Acylceramide | ACer | ACer (18:1) d18:1/17:0 | C_53_H_101_NO_4_ | 815.7731 | ++ |  |  |  |  | ++ |
| SP0205 | Ceramide 1-phosphate | CerP | CerP d18:1/16:0 | C_34_H_68_NO_6_P | 617.4784 | ++ |  |  | ++ |  |  |
| SP | Sphingosylphosphoethanolamine | PE-Sph | PE-SPB d18:1 | C_20_H_43_N_2_O_5_P | 422.2910 | ++ |  |  | ++ |  |  |
| SP | Sphingosylphosphoinositol | PI-Sph | PI-SPB d18:1 | C_24_H_48_NO_10_P | 541.3016 | ++ |  |  | ++ |  | ++ |
| SP0301 | Ceramide phosphocholine | SM | SM d18:1/18:0 | C_41_H_83_N_2_O_6_P | 730.5989 | ++ |  |  |  |  | ++ |
| SP0106 | Lysosphingomyelins and lysoglycosphingolipid | LSM | [*d*_9_] LSM d18:1 | C_23_H_40_D_9_N_2_O_5_P | 473.3944 | ++ |  |  |  |  | ++ |
| SP0302 | Ceramide phosphoethanolamine | PE-Cer | PE-Cer d17:1/12:0 | C_31_H_63_N_2_O_6_P | 590.4424 | ++ |  |  | ++ |  |  |
| SP0501, SP0700 | Simple Glc series | Hex-Sph | [*d*_5_] Hex-SPB d18:1 | C_24_H_42_D_5_NO_7_ | 466.3666 | ++ |  |  |  |  | ++ |
| SP0501 | Simple Glc series | HexCer-NDS | HexCer d18:0/22:0 | C_46_H_91_NO_8_ | 785.6745 | ++ |  |  | + |  | ++ |
| SP0501 | Simple Glc series | HexCer-NS | HexCer d18:1/16:0 | C_40_H_77_NO_8_ | 699.5649 | ++ |  |  | + |  | ++ |
| SP0501 | Simple Glc series | HexCer-NP | HexCer t18:0/26:0 | C_50_H_99_NO_9_ | 857.7320 | ++ |  |  |  |  | ++ |
| SP0502 | Galalpha1-4Galbeta1-4Glc- (Globo series) | Hex3Cer | GB3 d18:1/17:0 | C_53_H_99_NO_18_ | 1037.6862 | ++ |  | + | + |  | ++ |
| SP0503 | GalNAcbeta1-4Galbeta1-4Glc- (Ganglio series) | Hex2Cer | GA2 d18:1/17:0 | C_47_H_89_NO_13_ | 875.6334 | ++ |  | + | + |  | ++ |
| SP0601 | Ganglioside | GM3 | [*d*_5_] GM3 d18:1/18:0 | C_59_H_103_D_5_N_2_O_21_ | 1185.7758 | ++ |  | ++ | ++ |  |  |
| SP0601 | Ganglioside | GM1 | [*d*_5_] GM1 d18:1/18:0 | C_73_H_126_D_5_N_3_O_31_ | 1550.9080 | ++ |  | ++ | ++ |  |  |
| SP0602 | Sulfoglycosphingolipid (sulfatide) | SHexCer-AS | SHexCer d18:1/18:0(OH) | C_42_H_81_NO_12_S | 823.5479 | ++ |  |  | ++ |  | + |
| SP0602 | Sulfoglycosphingolipid (sulfatide) | SHexCer-NS | [*d*_7_] SHexCer d18:1/13:0 | C_37_H_64_D_7_NO_11_S | 744.5187 | ++ |  | ++ |  |  |  |
| ST0101 | Cholesterol and derivative | Chol | ST 27:1;O | C_27_H_46_O | 386.3549 | ++ |  |  |  |  |  |
| ST0102 | Steryl ester | CE | CE 16:0 | C_43_H_76_O_2_ | 624.5845 |  |  | ++ |  |  |  |
| ST0505 | Other Steroid conjugate | AHexCS | ST 27:1;O;Hex 14:0 | C_47_H_82_O_7_ | 758.6061 |  |  | ++ |  |  | ++ |

^a^ https://www.lipidmaps.org/

Ionization efficiencies were evaluated at two levels (+ and ++). Blank is "not detected".

**Supplementary Table 2**　Details of peak alignment and detection parameters

| 1. Peak alignment (node name, Align Retention Times) | Creating an exclusion list^a^ | Creating an inclusion list^b^ | Analysis of LC-FRC-HRMS full-scan data^c^ |
| --- | --- | --- | --- |
| Alignment Model | ‒ | ‒ | Adaptive curve |
| Maximum Retention Shift | ‒ | ‒ | 0.2 min |
| Mass Tolerance | ‒ | ‒ | 5 ppm |
| 2. Peak detection (node name, Detection Compounds) | | | |
| 2-1. General Settings |  |  |  |
| Mass Tolerance | 5 ppm | 5 ppm | 3 ppm |
| Intensity Tolerance | 30% | 30% | 30% |
| *S/N* Threshold | 10 | 10 | 10 |
| Minimum Peak Intensity | 1,000,000 | 1,000,000 | 100,000 |
| Ions | [M+H]^+^, [M‒H]^‒^ | [M+H]^+^, [M‒H]^‒^ | [M+H]^+^, [M+NH_4_]^+^, [M‒H]^‒^, [M‒H+HAc]^‒^ |
| Base Ions | [M+H]^+^, [M‒H]^‒^ | [M+H]^+^, [M‒H]^‒^ | [M+H]^+^, [M‒H]^‒^ |
| Minimum Element Counts | C, H | C, H | C, H |
| Maximum Element Counts | C200, H400, N10, O30, P10, S10 | C200, H400, N10, O30, P10, S10 | C200, H400, N10, O30, P10, S10 |
| 2-2. Peak Detection |  |  |  |
| Filter Peaks | FALSE | FALSE | TRUE |
| Maximum Peak Width | 0.5 min | 0.5 min | 0.5 min |
| Remove Singlets | TRUE | TRUE | TRUE |
| Minimum Scans Per Peak | 3 | 3 | 3 |
| Minimum Isotopes | 1 | 1 | 1 |
| 3. Data grouping (node name, Group Compounds) | | | |
| Mass Tolerance | ‒ | ‒ | 5 |
| RT Tolerance | ‒ | ‒ | 0.1 |
| 4. Gap filling (node name, Fill Gaps) | | | |
| Mass Tolerance | ‒ | 5 ppm | 5 ppm |
| *S/N* Threshold | ‒ | 1.5 | 1.5 |
| Filter Peaks | ‒ | ‒ | TRUE |
| 5. Background subtraction (node name, mark background compounds) | | | |
| Max. Sample/Blank | ‒ | ‒ | 4 |

^b^ Exclusion lists were generated using the blank sample (methanol/chloroform (1:1, *v/v*)) data obtained from the LC-FRC-HRMS in either the positive ion or negative ion mode.

^b^ Inclusion lists were generated using the *H. pylori* sample data obtained from the LC-FRC-HRMS in either the positive ion or negative ion mode.

^c^ *H. pylori* sample data obtained from LC-FRC-HRMS/MS analysis in positive or negative ion mode were used for analysis.

**Supplementary Table 3**　Comparative validation of the RTs and peak widths for each lipid subclass standard using InertSustain C18 (SUS) and PEEK-coated InertSustain C18 columns

| LIPID MAPS ID^a^ | Abbreviation of lipid subclass | Lipid species | InertSustain C18 (SUS) | | InertSustain C18 (PEEK) | | Peak width ratio |
| --- | --- | --- | --- | --- | --- | --- | --- |
|  |  |  | RT [min] | Peak width [min] | RT [min] | Peak width [min] | PEEK/SUS |
| FA0101 | FA | FA 32:6 | 37.96 | 0.67 | 38.62 | 0.66 | 0.99 |
| FA0102 | aFA | aFA 15:0 | 8.77 | 1.20 | 9.07 | 1.00 | 0.83 |
| FA0105 | HFA | HFA 18:1 | 3.34 | 0.28 | 3.02 | 0.22 | 0.79 |
| FA0116 | α-MA | FA80:2;O | 73.90 | 0.56 | 75.54 | 0.54 | 0.96 |
| FA0116 | keto-MA | FA 86:2;O2 | 72.16 | 0.58 | 72.28 | 0.58 | 1.00 |
| FA0116 | methoxy-MA | FA 85:1;O2 | 73.59 | 0.67 | 74.25 | 0.60 | 0.90 |
| FA0600 | Fatty aldehydes | [*d*_9_] Fattty aldehyde 16:0 | 36.76 | 0.62 | 37.52 | 0.37 | 0.60 |
| FA0707 | CAR | CAR 16:0 | 4.96 | 1.80 | 5.10 | 1.30 | 0.72 |
| FA0709 | FAHFA | FAHFA 18:0/9-O-16:0 | 47.55 | 0.64 | 48.21 | 0.61 | 0.95 |
| FA0802 | NAGly | NAGly 18:1 | 3.32 | 0.15 | 3.31 | 0.15 | 1.00 |
| FA0802 | NAHC | NAHC 16:0 | 3.26 | 0.15 | 3.25 | 0.17 | 1.13 |
| FA0802 | NATau | NATau 16:0 | 3.30 | 0.20 | 3.00 | 0.17 | 0.85 |
| GL0101 | MG | MG 16:0 | 16.67 | 0.67 | 17.21 | 0.67 | 1.00 |
| GL0102 | Ether MG | Ether MG O-16:0 | 21.22 | 0.92 | 21.95 | 0.79 | 0.86 |
| GL0103 | Ether MG (P) | Ether MG P-18:0 | 28.44 | 0.86 | 28.89 | 0.67 | 0.78 |
| GL0201 | DG | DG 18:0/20:4 | 50.91 | 0.62 | 51.50 | 0.62 | 1.00 |
| GL0301 | TG | TG 18:0/18:0/18:0 | 67.53 | 0.60 | 68.13 | 0.69 | 1.15 |
| GP0101 | PC | PC 16:0/18:1 | 43.04 | 0.83 | 43.79 | 0.74 | 0.89 |
| GP0102 | EtherPC | PC O-16:0/20:4 | 42.05 | 0.72 | 42.83 | 0.65 | 0.90 |
| GP0103 | EtherPC (P) | PC P-18:0/20:4 | 44.84 | 0.82 | 45.57 | 0.77 | 0.94 |
| GP0105 | LPC | LPC 18:0 | 8.10 | 0.92 | 8.56 | 0.85 | 0.92 |
| GP0106 | EtherLPC | LPC O-18:0 | 9.66 | 1.13 | 10.26 | 1.22 | 1.08 |
| GP0107 | EtherLPC (P) | LPC P-18:0 | 9.68 | 1.13 | 10.16 | 1.65 | 1.46 |
| GP2001 | OxPC | OxPC 16:0/9:0;O | 4.06 | 8.58 | 4.79 | 8.39 | 0.98 |
| GP2001 | OxPC | OxPC 16:0/4:0;O2 | 2.68 | 0.39 | 3.01 | 0.17 | 0.44 |
| GP0201 | PE | PE 18:0/20:4 | 44.44 | 0.84 | 45.16 | 0.86 | 1.02 |
| GP2103 | EtherPE (P) | PE P-18:0/20:4 | 45.92 | 0.71 | 46.65 | 0.71 | 1.00 |
| GP0201 | MPE | MPE 16:0/16:0 | 43.67 | 0.64 | 44.41 | 0.64 | 1.00 |
| GP0205 | LPE | LPE 18:0 | 7.88 | 1.24 | 8.32 | 1.04 | 0.84 |
| GP0207 | EtherLPE (P) | LPE P-18:0 | 9.64 | 0.90 | 10.21 | 0.81 | 0.90 |
| GP | Lac-PE | Lac-PE 18:1 | 41.21 | 1.54 | 42.05 | 0.94 | 0.61 |
| GP | Suc-PE | Suc-PE 18:1/18:1 | 36.45 | 0.86 | 37.63 | 0.66 | 0.77 |
| GP0301 | PS | PS 18:0/18:1 | 43.47 | 13.24 | 43.28 | 2.02 | 0.15 |
| GP0301 | NAPS | NAPS 18:1/18:1/19:0 | 53.47 | 2.56 | 54.00 | 0.79 | 0.31 |
| GP0305 | LPS | LPS 16:0 | 3.43 | 2.48 | 3.16 | 0.54 | 0.22 |
| GP0401 | PG | PG 16:0/18:1 | 39.64 | 0.80 | 40.75 | 0.57 | 0.71 |
| GP | Lysyl-PG | Lysyl-PG 16:0/16:0 | 41.65 | 1.95 | 42.34 | 1.64 | 0.84 |
| GP0405 | LPG | LPG 18:0 | 6.26 | 1.04 | 6.85 | 0.85 | 0.82 |
| GP0409 | SLBPA | SLBPA 18:1/18:1/16:0 | 53.26 | 0.75 | 54.13 | 0.76 | 1.01 |
| GP0410 | LBPA | LBPA 18:1/18:1 | 38.59 | 0.55 | 40.01 | 0.53 | 0.96 |
| GP0408 | BPA | BPA 18:1/18:1/18:1/17:0 | 61.59 | 0.58 | 62.38 | 0.58 | 1.00 |
| GP0601 | PI | PI 18:0/20:4 | 38.89 | 1.24 | 40.04 | 1.01 | 0.81 |
| GP0605 | LPI | LPI 18:0 | 4.71 | 0.85 | 5.07 | 0.91 | 1.07 |
| GP0603 | PIP | PIP 18:1/18:1 | n.d. | ‒ | 26.16, 33.50 | 18.93 | ‒ |
| GP0801 | PIP2 | PIP2 18:1/18:1 | n.d. | ‒ | 11.05 | 15.06 | ‒ |
| GP0901 | PIP3 | PIP3 18:1/18:1 | n.d. | ‒ | 2.94 | 0.24 | ‒ |
| GP1001 | PA | PA 16:0/18:1 | n.d. | ‒ | 41.49 | 1.52 | ‒ |
| GP1005 | LPA | LPA 18:0 | n.d. | ‒ | 7.17 | 1.93 | ‒ |
| GP1101 | DGPP | DGPP 18:1/18:1 | n.d. | ‒ | 35.59, 41.78 | 21.80 | ‒ |
| GP1201 | CL | [*d*_5_] CL 18:2/18:2 | 54.69 | 0.97 | 55.67 | 0.71 | 0.73 |
| GP1301 | CDP-DG | CDP-DG 16:0/16:0 | 30.92 | 6.46 | 32.12 | 1.42 | 0.22 |
| GP2501 | CPA | CPA 17:0 | 6.87 | 1.54 | 7.57 | 1.44 | 0.94 |
| GP | PTE | PTE 16:0/16:0 | 41.61 | 0.58 | 42.70 | 0.56 | 0.97 |
| SP0101 | Sph | [*d*_7_] SPB d18:1 | 6.18 | 1.87 | 6.13 | 1.48 | 0.79 |
| SP0102 | DHSph | [*d*_7_] SPB d18:0 | 6.39 | 1.39 | 6.19 | 1.21 | 0.87 |
| SP0105 | Sph-P | [*d*_7_] SPBP d18:1 | n.d. | ‒ | 2.62 | 2.81 | ‒ |
| SP0105 | DHSph-P | [*d*_7_] SPBP d18:0 | n.d. | ‒ | 2.83 | 2.60 | ‒ |
| SP | DOSph | SPB m18:0 | 7.50 | 1.37 | 7.15 | 0.97 | 0.71 |
| SP | - | 1-desoxymethyl SPB m17:0 | 6.68 | 2.11 | 6.37 | 1.92 | 0.91 |
| SP0107 | - | *N,N*-Dimethyl-SPB d17:1 | 6.85 | 2.60 | 6.38 | 0.54 | 0.21 |
| SP0105 | - | SPBP t18:0 | n.d. | ‒ | 2.55 | 2.73 | ‒ |
| SP0101 | - | 3-keto-SPB d12:0 | 2.98 | 0.67 | 2.95 | 0.72 | 1.07 |
| SP0108 | - | SPB d18:2 | 3.28 | 0.29 | 3.27 | 0.24 | 0.83 |
| SP0201 | Cer-AS | Cer d18:1/18:0(2OH) | 46.27 | 0.68 | 46.96 | 0.69 | 1.01 |
| SP0201 | Cer-NS | Cer d18:1/16:0 | 44.69 | 0.66 | 45.40 | 0.61 | 0.92 |
| SP0202 | Cer-NDS | [*d*_9_] Cer d18:0/16:0 | 45.78 | 0.70 | 46.53 | 0.63 | 0.90 |
| SP0203 | Cer-NP | Cer t18:0/24:0 | 54.13 | 0.67 | 54.78 | 0.71 | 1.06 |
| SP0203 | Cer-AP | [*d*_9_] Cer t18:0/16:0(2OH) | 40.80 | 0.82 | 41.47 | 0.76 | 0.93 |
| SP0204 | Cer-EOP | Cer t18:0/26:0/26-O-18:1 | 60.98 | 0.94 | 61.89 | 0.49 | 0.52 |
| SP0204 | Cer-EOS | [*d*_9_] Cer t18:1/26:0/26-O-18:1 | 61.87 | 0.51 | 62.42 | 0.60 | 1.18 |
| SP0204 | ACer | ACer (18:1) d18:1/17:0 | 59.84 | 0.87 | 60.36 | 0.85 | 0.98 |
| SP0205 | CerP | CerP d18:1/16:0 | n.d. | ‒ | 35.50 | 2.41 | ‒ |
| SP | PE-Sph | PE-SPB d18:1 | 2.83 | 0.68 | 2.77 | 0.54 | 0.79 |
| SP | PI-Sph | PI-SPB d18:1 | 2.41 | 0.70 | 2.42 | 0.56 | 0.80 |
| SP0301 | SM | SM d18:1/18:0 | 42.02 | 0.75 | 42.88 | 0.70 | 0.93 |
| SP0106 | LSM | [*d*_9_] LSM d18:1 | 2.82 | 0.66 | 2.74 | 0.52 | 0.79 |
| SP0302 | PE-Cer | PE-Cer d17:1/12:0 | 20.59 | 2.02 | 20.26 | 1.20 | 0.59 |
| SP0501, SP0700 | Hex-Sph | [*d*_5_] Hex-SPB d18:1 | 3.32 | 0.20 | 3.29 | 0.22 | 1.10 |
| SP0501 | HexCer-NDS | HexCer d18:0/22:0 | 50.57 | 0.99 | 51.52 | 0.94 | 0.95 |
| SP0501 | HexCer-NS | HexCer d18:1/16:0 | 40.28 | 0.69 | 41.09 | 0.64 | 0.93 |
| SP0501 | HexCer-NP | HexCer t18:0/26:0 | 53.31 | 0.51 | 54.02 | 0.51 | 1.00 |
| SP0502 | Hex3Cer | GB3 d18:1/17:0 | 38.61 | 0.74 | 39.50 | 0.65 | 0.88 |
| SP0503 | Hex2Cer | GA2 d18:1/17:0 | 39.79 | 0.64 | 40.67 | 0.62 | 0.97 |
| SP0601 | GM3 | [*d*_5_] GM3 d18:1/18:0 | 36.12 | 0.71 | 37.59 | 0.71 | 1.00 |
| SP0601 | GM1 | [*d*_5_] GM1 d18:1/18:0 | 33.93 | 1.33 | 35.59 | 0.82 | 0.62 |
| SP0602 | SHexCer-AS | SHexCer d18:1/18:0(OH) | 36.64 | 1.53 | 38.37 | 0.77 | 0.50 |
| SP0602 | SHexCer-NS | [*d*_7_] SHexCer d18:1/13:0 | 24.82 | 0.83 | 26.59 | 0.73 | 0.88 |
| ST0101 | Chol | ST 27:1;O | 40.65 | 0.41 | 41.38 | 0.35 | 0.85 |
| ST0102 | CE | CE 16:0 | 49.48 | 3.84 | 50.22 | 2.87 | 0.75 |
| ST0505 | AHexCS | ST 27:1;O;Hex 14:0 | 51.70 | 0.56 | 52.46 | 0.53 | 0.95 |
| Average |  |  |  |  |  |  | 0.85 |

^a^ https://www.lipidmaps.org/

n.d.: not detected.

**Supplementary Table 4**　Fatty acid composition used for setting the theoretically calculated comprehensive lipid library

| Fatty acid | Abbreviation |  | Fatty acid | Abbreviation |
| --- | --- | --- | --- | --- |
| Lauric acid | FA 12:0 |  | Eicosadienoic acid (*n‒*6) | FA 20:2 |
| Tridecanoic acid | FA 13:0 |  | Dihomo-g-linoleic acid (*n‒*6) | FA 20:3 |
| Myristic acid | FA 14:0 |  | Arachidonic acid (*n‒*6) | FA 20:4 |
| Myristoleic acid (*n‒*5) | FA 14:1 |  | Eicosapentaenoic acid (*n‒*3) | FA 20:5 |
| Pentadecylic acid | FA 15:0 |  | Behenic acid | FA 20:6 |
| Palmitic acid | FA 16:0 |  | Heneicosanoic acid | FA 21:0 |
| Palmitoleic acid (*n‒*7) | FA 16:1 |  | Erucic acid (*n‒*9) | FA 22:1 |
| Heptadecanoic acid | FA 17:0 |  | Docosadienoic acid (*n‒*6) | FA 22:2 |
| *cis*-10-Heptadecenoic acid (*n*‒7) | FA 17:1 |  | Docosatorienoic acid (*n‒*3) | FA 22:3 |
| Stearic acid | FA 18:0 |  | Docosatetraenoic acid (*n‒*6) | FA 22:4 |
| Oleic acid (*n‒*9) | FA 18:1 |  | Docosapentaenoic acid (*n‒*3) | FA 22:5 |
| Linoleic acid (*n‒*6) | FA 18:2 |  | Docosahexaenoic acid (*n‒*3) | FA 22:6 |
| a-Linoleic acid (*n‒*3) | FA 18:3 |  | Tricosanoic acid | FA 23:0 |
| Stearidonic acid (*n‒*3) | FA 18:4 |  | Lignoceric acid | FA 24:0 |
| Nonadecylic acid | FA 19:0 |  | Nervonic acid (*n*‒9) | FA 24:1 |
| *cis*-10-Nonadecenoic acid (*n*‒9) | FA 19:1 |  | Pentacosanoic acid | FA 25:0 |
| Arachidic acid | FA 20:0 |  | Cerotic acid (hexacosanoic acid) | FA 26:0 |
| Eicosenoic acid (*n‒*9) | FA 20:1 |  |  |  |

Double bond positions (*n*)

**Supplementary Table 5**　Summary of key substructure information from the HRMS/MS spectra of each lipid class standard for lipid annotation

| LIPID MAPS ID^a^ | Abbreviation  of lipid subclass | Lipid species | Positive ion mode (ESI+) | | Negative ion mode (ESI–) | |
| --- | --- | --- | --- | --- | --- | --- |
|  |  |  | Major adduct ion (MS^1^) | Major product ion(s) (MS^2^) with characteristic substructure(s) | Major adduct ion (MS^1^) | Major product ion(s) (MS^2^) with characteristic substructure(s) |
| FA0101 | FA | FA 32:6 | - | - | [M ‒ H]^‒^ | [M – H]^‒^ |
| FA0102 | aFA | aFA 15:0 | - | - | [M ‒ H]^‒^ | [M – H]^‒^ |
| FA0105 | HFA | HFA 18:1 | - | - | [M ‒ H]^‒^ | [M – H – CH_2_O_2_]^‒^ |
| FA0116 | α-MA | FA80:2;O | [M + NH_4_]^+^ | [M + NH_4_ – NH_3_ – CH_4_O_3_]^+^ | [M ‒ H]^‒^ | [Acyl FA – H]^‒^ |
| FA0116 | keto-MA | FA 86:2;O2 | [M + H]^+^ | [M + H – Acyl FA]^+^ | [M ‒ H]^‒^ | [Acyl FA – H]^‒^ |
| FA0116 | methoxy-MA | FA 85:1;O2 | [M + H]^+^ | [M + H – CH_6_O_2_]^+^ | [M ‒ H]^‒^ | [Acyl FA – H]^‒^ |
| FA0600 | Fatty aldehydes | [*d*_9_] Fattty aldehyde 16:0 | [M + H]^+^ | [M + H]^+^ | - | - |
| FA0707 | CAR | CAR 16:0 | [M + H]^+^ | [C_4_H_4_O_2_+ H]^+^ | [M ‒ H]^‒^ | [Acyl FA – H]^‒^ |
| FA0709 | FAHFA | FAHFA 16:0/9-O-18:0 | [M + NH_4_]^+^ | [Acyl FA + H]^+^ | [M ‒ H]^‒^ | [Acyl FA – H]^‒^ |
| FA0802 | NAGly | NAGly 18:1 | [M + H]^+^ | [C_2_H_5_NO_2_ + H]^+^ | [M ‒ H]^‒^ | [M – H – CO_2_]^‒^ |
| FA0802 | NAHC | NAHC 16:0 | [M + H]^+^ | [C_4_H_9_NO_2_S + H]^+^ | [M ‒ H]^‒^ | [Acyl + C_2_H_4_N – H]^‒^ |
| FA0802 | NATau | NATau 16:0 | [M + H]^+^ | [C_2_H_7_NO_3_S + H]^+^ | [M ‒ H]^‒^ | [Acyl amide – H]^‒^ |
| GL0101 | MG | MG 16:0 | [M + H]^+^ | [M + H – H_2_O]^+^ | - | - |
| GL0102 | Ether MG | Ether MG O-16:0 | [M + H]^+^ | [C_3_H_8_O_3_ + H]^+^ | - | - |
| GL0103 | Ether MG (P) | Ether MG P-18:0 | [M + H]^+^ | [C_3_H_6_O_2_ + H]^+^ | - | - |
| GL0201 | DG | DG 18:0/20:4 | [M + H]^+^ | [M + H – Acyl FA (*sn*-1)]^+^  [M + H – Acyl FA (*sn*-2)]^+^ | - | - |
| GL0301 | TG | TG 18:0/18:0/18:0 | [M + NH_4_]^+^ | [M + NH_4_ – Acyl FA (*sn*-1)]^+^  [M + NH_4_ – Acyl FA (*sn*-2)]^+^  [M + NH_4_ – Acyl FA (*sn*-3)]^+^ | - | - |
| GP0101 | PC | PC 16:0/18:1 | [M + H]^+^ | [C_5_H_14_NO_4_P + H]^+^ | [M + CH_3_COO]^‒^ | [Acyl FA (*sn*-1) – H]^‒^  [Acyl FA (*sn*-2) – H]^‒^ |
| GP0102 | EtherPC | PC O-16:0/20:4 | [M + H]^+^ | [C_5_H_14_NO_4_P + H]^+^ | [M + CH_3_COO]^‒^ | [Acyl FA – H]^‒^ |
| GP0103 | EtherPC (P) | PC P-18:0/20:4 | [M + H]^+^ | [C_5_H_14_NO_4_P + H]^+^ | [M + CH_3_COO]^‒^ | [Acyl FA – H]^‒^  [Acyl ether – H]^‒^ |
| GP0105 | LPC | LPC 18:0 | [M + H]^+^ | [C_5_H_14_NO_4_P + H]^+^ | [M + CH_3_COO]^‒^ | [Acyl FA – H]^‒^ |
| GP0106 | EtherLPC | LPC O-18:0 | [M + H]^+^ | [C_5_H_14_NO_4_P + H]^+^ | [M + CH_3_COO]^‒^ | [M + CH_3_COO – CH_3_COOH – C_5_H_13_NO]^‒^ |
| GP0107 | EtherLPC (P) | LPC P-18:0 | [M + H]^+^ | [C_5_H_14_NO_4_P + H]^+^ | [M + CH_3_COO]^‒^ | [Acyl ether – H]^‒^ |
| GP2001 | OxPC | OxPC 16:0/9:0;O | [M + H]^+^ | [C_5_H_14_NO_4_P + H]^+^ | [M + CH_3_COO]^‒^ | [Acyl FA – H]^‒^  [Acyl FA;O – H]^‒^ |
| GP2001 | OxPC | OxPC 16:0/4:0;O2 | [M + H]^+^ | [C_5_H_14_NO_4_P + H]^+^ | [M ‒ H]^‒^ | [Acyl FA – H]^‒^  [Acyl FA;O2 – H]^‒^ |
| GP0201 | PE | PE 18:0/20:4 | [M + H]^+^ | [M + H – C_2_H_8_NO_4_P]^+^ | [M ‒ H]^‒^ | [Acyl FA (*sn*-1) – H]^‒^  [Acyl FA (*sn*-2) – H]^‒^ |
| GP2103 | EtherPE (P) | PE P-18:0/20:4 | [M + H]^+^ | [M + H – C_2_H_8_NO_4_P]^+^  [Acyl ether + C_2_H_7_NO_3_P + H]^+^ | [M ‒ H]^‒^ | [Acyl FA – H]^‒^  [Acyl ether – H]^‒^ |
| GP0201 | MPE | MPE 16:0/16:0 | [M + H]^+^ | [M + H – C_3_H_10_NO_4_P]^+^ | [M ‒ H]^‒^ | [Acyl FA (*sn*-1) – H]^‒^  [Acyl FA (*sn*-2) – H]^‒^ |
| GP0205 | LPE | LPE 18:0 | [M + H]^+^ | [M + H – C_2_H_8_NO_4_P]^+^ | [M ‒ H]^‒^ | [Acyl FA – H]^‒^ |
| GP0207 | EtherLPE (P) | LPE P-18:0 | [M + H]^+^ | [Acyl FA ether + C_2_H_7_NO_3_P + H]^+^ | [M ‒ H]^‒^ | [Acyl ether – H]^‒^ |
| GP | Lac-PE | Lac-PE 18:1 | [M + H]^+^ | [M + H – C_14_H_30_NO_14_P]^+^ | [M ‒ H]^‒^ | [Acyl FA (*sn*-1) – H]^‒^  [Acyl FA (*sn*-2) – H]^‒^ |
| GP | Suc-PE | Suc-PE 18:1/18:1 | [M + H]^+^ | [M + H – C_6_H_12_NO_7_P]^+^ | [M ‒ H]^‒^ | [Acyl FA (*sn*-1) – H]^‒^  [Acyl FA (*sn*-2) – H]^‒^ |
| GP0301 | PS | PS 18:0/18:1 | [M + H]^+^ | [M + H – C_3_H_8_NO_6_P]^+^ | [M ‒ H]^‒^ | [Acyl FA (*sn*-1) – H]^‒^  [Acyl FA (*sn*-2) – H]^‒^ |
| GP0301 | NAPS | NAPS 18:1/18:1/19:0 | [M + H]^+^ | [Acyl + C_3_H_4_NO_2_ + H]^+^ | [M ‒ H]^‒^ | [Acyl FA (*sn*-1) – H]^‒^  [Acyl FA (*sn*-2) – H]^‒^  [M – H – Acyl (N-acyl) – C_3_H_4_NO_2_]^‒^ |
| GP0305 | LPS | LPS 16:0 | [M + H]^+^ | [M + H – C_3_H_8_NO_6_P]^+^ | [M ‒ H]^‒^ | [Acyl FA – H]^‒^ |
| GP0401 | PG | PG 16:0/18:1 | [M + H]^+^ | [M + H – C_3_H_9_O_6_P]^+^ | [M ‒ H]^‒^ | [Acyl FA (*sn*-1) – H]^‒^  [Acyl FA (*sn*-2) – H]^‒^ |
| GP | Lysyl-PG | Lysyl-PG 16:0/16:0 | [M + H]^+^ | [C_9_H_21_N_2_O_7_P + H]^+^ | [M ‒ H]^‒^ | [Acyl FA (*sn*-1) – H]^‒^  [Acyl FA (*sn*-2) – H]^‒^ |
| GP0405 | LPG | LPG 18:0 | [M + H]^+^ | [M + H – C_3_H_9_O_6_P]^+^ | [M ‒ H]^‒^ | [Acyl FA – H]^‒^ |
| GP0409 | SLBPA | SLBPA 18:1/18:1/16:0 | [M + H]^+^ | [M + H – C_3_H_5_O_4_P – Acyl FA (*sn*-1) – Acyl FA (*sn*-2)]^+^  [M + H – C_3_H_7_O_5_P – Acyl FA (*sn*-1')]^+^ | [M ‒ H]^‒^ | [Acyl FA (*sn*-1) – H]^‒^  [Acyl FA (sn-2) – H]^‒^  [Acyl FA (*sn*-1') – H]^‒^ |
| GP0410 | LBPA | LBPA 18:1/18:1 | [M + H]^+^ | [M + H – C_3_H_7_O_5_P – Acyl FA (*sn*-1)]^+^  [M + H – C_3_H_7_O_5_P – Acyl FA (*sn*-1')]^+^ | [M ‒ H]^‒^ | [Acyl FA (*sn*-1) – H]^‒^  [Acyl FA (*sn*-1') – H]^‒^ |
| GP0408 | BPA | BPA 18:1/18:1/18:1/17:0 | [M + H]^+^ | [M + H – C_3_H_5_O_4_P – Acyl FA (*sn*-1) – Acyl FA (*sn*-2)]^+^  [M + H – C_3_H_5_O_4_P – Acyl FA (*sn*-1') – Acyl FA (*sn*-2')]^+^ | [M ‒ H]^‒^ | [Acyl FA (*sn*-1) – H]^‒^  [Acyl FA (sn-2) – H]^‒^  [Acyl FA (sn-1') – H]^‒^  [Acyl FA (*sn*-2') – H]^‒^ |
| GP0601 | PI | PI 18:0/20:4 | [M + H]^+^ | [M + H – C_6_H_13_O_9_P]^+^ | [M ‒ H]^‒^ | [Acyl FA (*sn*-1) – H]^‒^  [Acyl FA (*sn*-2) – H]^‒^ |
| GP0605 | LPI | LPI 18:0 | [M + H]^+^ | [M + H – C_6_H_13_O_9_P]^+^ | [M ‒ H]^‒^ | [Acyl FA – H]^‒^ |
| GP0603 | PIP | PIP 16:0/16:0 | [M + H]^+^ | [M + H – C_6_H_14_O_12_P_2_]^+^ | [M ‒ H]^‒^ | [Acyl FA (*sn*-1) – H]^‒^  [Acyl FA (*sn*-2) – H]^‒^ |
| GP0801 | PIP2 | PIP2 17:0/20:4 | [M + H]^+^ | [M + H – C_6_H_15_O_15_P_3_]^+^ | [M ‒ H]^‒^ | [Acyl FA (*sn*-1) – H]^‒^  [Acyl FA (*sn*-2) – H]^‒^ |
| GP0901 | PIP3 | PIP3 16:0/16:0 | [M + H]^+^ | [M + H – C_6_H_16_O_18_P_4_]^+^ | [M ‒ H]^‒^ | [Acyl FA (*sn*-1) – H]^‒^  [Acyl FA (*sn*-2) – H]^‒^ |
| GP1001 | PA | PA 16:0/18:1 | [M + NH_4_]^+^ | [M + NH_4_ – NH_3_ – H_3_O_4_P]^+^ | [M ‒ H]^‒^ | [Acyl FA (*sn*-1) – H]^‒^  [Acyl FA (*sn*-2) – H]^‒^ |
| GP1005 | LPA | LPA 18:0 | [M + NH_4_]^+^ | [M + NH_4_ – NH_3_ – H_3_O_4_P]^+^ | [M ‒ H]^‒^ | [Acyl FA – H]^‒^ |
| GP1101 | DGPP | DGPP 18:1/18:1 | [M + NH_4_]^+^ | [M + NH_4_ – NH_3_ – H_4_O_7_P_2_]^+^ | [M ‒ H]^‒^ | [Acyl FA (*sn*-1) – H]^‒^  [Acyl FA (*sn*-2) – H]^‒^ |
| GP1201 | CL | [*d*_5_] CL 18:2/18:2 | [M + H]^+^ | [M + H – PA (*sn*-1, *sn*-2) – C_3_H_2_^2^H_5_O_5_P]^+^  [M + H – PA (*sn*-1', *sn*-2') – C_3_H_2_^2^H_5_O_5_P]^+^ | [M ‒ H]^‒^ | [Acyl FA (*sn*-1) – H]^‒^  [Acyl FA (*sn*-2) – H]^‒^, [Acyl FA (*sn*-1') – H]^‒^  [Acyl FA (*sn*-2') – H]^‒^ |
| GP1301 | CDP-DG | CDP-DG 16:0/16:0 | [M + H]^+^ | [M + H – C_9_H_15_N_3_O_11_P_2_]^+^ | [M ‒ H]^‒^ | [Acyl FA (*sn*-1) – H]^‒^  [Acyl FA (*sn*-2) – H]^‒^ |
| GP0000 | CPA | CPA 17:0 | [M + NH_4_]^+^ | [C_3_H_7_O_5_P + H]^+^ | [M ‒ H]^‒^ | [Acyl FA – H]^‒^ |
| GP | PTE | PTE 16:0/16:0 | [M + NH_4_]^+^ | [M + NH_4_ ‒ NH_3_ – C_2_H_7_O_4_PS]^+^ | [M ‒ H]^‒^ | [Acyl FA (*sn*-1) – H]^‒^  [Acyl FA (*sn*-2) – H]^‒^ |
| SP0101 | Sph | [*d*_7_] SPB d18:1 | [M + H]^+^ | [SPB d18:1 + H – H_2_O]^+^ | - | - |
| SP0102 | DHSph | [*d*_7_] SPB d18:0 | [M + H]^+^ | [SPB d18:0 + H – H_2_O]^+^ | - | - |
| SP0105 | Sph-P | [*d*_7_] SPBP d18:1 | [M + H]^+^ | [SPB d18:1 + H – 2H_2_O]^+^ | [M ‒ H]^‒^ | [PO_3_]^‒^ |
| SP0105 | DHSph-P | [*d*_7_] SPBP d18:0 | [M + H]^+^ | [SPB d18:0 + H – 2H_2_O]^+^ | [M ‒ H]^‒^ | [PO_3_]^‒^ |
| SP | DOSph | SPB m18:0 | [M + H]^+^ | [SPB m18:0 + H – H_2_O]^+^ | - | - |
| SP | - | 1-desoxymethyl SPB m17:0 | [M + H]^+^ | [M + H – H_2_O]^+^ | - | - |
| SP0107 | - | *N,N*-Dimethyl-SPB d17:1 | [M + H]^+^ | [M + H – CH_4_O_2_]^+^ | - | - |
| SP0105 | - | SPBP t18:0 | [M + H]^+^ | [SPB t18:0 + H – H_2_O]^+^ | [M ‒ H]^‒^ | [PO_3_]^‒^ |
| SP0101 | - | 3-keto-SPB d12:0 | [M + H]^+^ | [M + H – CH_2_O]^+^ | - | - |
| SP0108 | - | SPB d18:2 | [M + H]^+^ | [SPB d18:2 + H – H_2_O]^+^ | - | - |
| SP0201 | Cer-AS | Cer d18:1/18:0(2OH) | [M + H]^+^ | [SPB d18:1 + H – 2H_2_O]^+^ | [M ‒ H]^‒^ | [Acyl;O – H]^‒^ |
| SP0201 | Cer-NS | Cer d18:1/16:0 | [M + H]^+^ | [SPB d18:1 + H – 2H_2_O]^+^ | [M ‒ H]^‒^ | [Acyl + C_2_H_4_N – H]^‒^ |
| SP0202 | Cer-NDS | [*d*_9_] Cer d18:0/16:0 | [M + H]^+^ | [SPB d18:0 + H – 2H_2_O]^+^ | [M ‒ H]^‒^ | [Acyl + C_2_H_4_N – H]^‒^ |
| SP0203 | Cer-NP | Cer t18:0/24:0 | [M + H]^+^ | [SPB t18:0 + H – 2H_2_O]^+^ | [M ‒ H]^‒^ | [Acyl + C_2_H_5_NO – H]^‒^ |
| SP0203 | Cer-AP | [*d*_9_] Cer t18:0/16:0(2OH) | [M + H]^+^ | [SPB t18:0 + H – 2H_2_O]^+^ | [M ‒ H]^‒^ | [Acyl;O – H]^‒^ |
| SP0204 | Cer-EOP | Cer t18:0/26:0/26-O-18:1 | [M + H]^+^ | [SPB t18:0 + H – 2H_2_O]^+^  [M + H – Acyl FA]^+^ | [M + CH_3_COO]^‒^ | [Acyl FA – H]^‒^  [Acyl + C_2_H_5_NO – H]^‒^ |
| SP0204 | Cer-EOS | [*d*_9_] Cer d18:1/26:0/26-O-18:1 | [M + H]^+^ | [SPB d18:1 + H – 2H_2_O]^+^ | [M + CH_3_COO]^‒^ | [Acyl FA – H]^‒^  [Acyl + C_2_H_4_N – H]^‒^ |
| SP0204 | ACer | ACer (18:1) d18:1/17:0 | [M + H]^+^ | [SPB d18:1 + H – 2H_2_O]^+^ | [M + CH_3_COO]^‒^ | [Acyl FA – H]^‒^  [Acyl;O + C_2_H_4_N – H]^‒^ |
| SP0205 | CerP | CerP d18:1/16:0 | [M + H]^+^ | [SPB d18:1 + H – 2H_2_O]^+^ | [M ‒ H]^‒^ | [PO_3_]^‒^ |
| SP | PE-Sph | PE-SPB d18:1 | [M + H]^+^ | [SPB d18:1 + H – 2H_2_O]^+^ | [M ‒ H]^‒^ | [C_2_H_8_NO_4_P – H]^‒^ |
| SP | PI-Sph | PI-SPB d18:1 | [M + H]^+^ | [SPB d18:1 + H – 2H_2_O]^+^ | [M ‒ H]^‒^ | [C_6_H_11_O_8_P – H]^‒^ |
| SP0301 | SM | SM d18:1/18:0 | [M + H]^+^ | [C_5_H_14_NO_4_P + H]^+^ | - | - |
| SP0106 | LSM | [*d*_9_] LSM d18:1 | [M + H]^+^ | [SPB d18:1 + H – 2H_2_O]^+^ | [M + CH_3_COO]^‒^ | [C_4_H_6_^2^H_6_NO_4_P – H]^‒^ |
| SP0302 | PE-Cer | PE-Cer d17:1/12:0 | [M + H]^+^ | [SPB d17:1 + H – 2H_2_O]^+^ | [M ‒ H]^‒^ | [C_2_H_8_NO_4_P – H]^‒^ |
| SP0501, SP0700 | Hex-Sph | [*d*_5_] Hex-SPB d18:1 | [M + H]^+^ | [SPB d18:1 + H – H_2_O]^+^ | [M ‒ H]^‒^ | [C_6_H_10_O_5_ – H]^‒^ |
| SP0501 | HexCer-NDS | HexCer d18:0/22:0 | [M + H]^+^ | [SPB d18:0 + H – 2H_2_O]^+^ | [M ‒ H]^‒^ | [Acyl + C_2_H_4_N – H]^‒^ |
| SP0501 | HexCer-NS | HexCer d18:1/16:0 | [M + H]^+^ | [SPB d18:1 + H – 2H_2_O]^+^ | [M ‒ H]^‒^ | [Acyl + C_2_H_4_N – H]^‒^ |
| SP0501 | HexCer-NP | HexCer t18:0/26:0 | [M + H]^+^ | [SPB t18:0 + H – 2H_2_O]^+^ | [M ‒ H]^‒^ | [Acyl + C_3_H_5_NO – H]^‒^ |
| SP0502 | Hex3Cer | GB3 d18:1/17:0 | [M + H]^+^ | [M + H – C_6_H_10_O_5_]^+^  [M + H – 2C_6_H_10_O_5_]^+^  [M + H – 3C_6_H_10_O_5_]^+^  [SPB d18:1 – 2H_2_O + H]^+^ | [M ‒ H]^‒^ | [Acyl + C_2_H_4_N – H]^‒^ |
| SP0503 | Hex2Cer | GA2 d18:1/17:0 | [M + H]^+^ | [M + H – C_6_H_10_O_5_]^+^  [M + H – 2C_6_H_10_O_5_]^+^  [SPB d18:1 ‒ 2H_2_O + H]^+^ | [M ‒ H]^‒^ | [Acyl + C_2_H_4_N – H]^‒^ |
| SP0601 | GM3 | [*d*_5_] GM3 d18:1/17:0 | [M + H]^+^ | [M + H – C_11_H_17_NO_8_]^+^, [M + H – C_11_H_17_NO_8_ – C_6_H_10_O_5_]^+^  [M + H – C_11_H_17_NO_8_ – 2C_6_H_10_O_5_]^+^  [SPB d18:1 – 2H_2_O + H]^+^ | [M ‒ H]^‒^ | [Acyl + C_2_H_4_N – H]^–^  [C_11_H_17_NO_8_ – H]^‒^ |
| SP0601 | GM1 | [*d*_5_] GM1 d18:1/17:0 | [M + NH_4_]^+^ | [M + NH_4_ – NH_3_ – C_14_H_23_NO_10_]^+^  [M + NH_4_ – NH_3_ – C_14_H_23_NO_10_ – C_11_H_17_NO_8_]^+^  [M + NH_4_ – NH_3_ – C_14_H_23_NO_10_ – C_11_H_17_NO_8_ – C_6_H_10_O_5_]^+^ [M + NH_4_ – NH_3_ – C_14_H_23_NO_10_ – C_11_H_17_NO_8_ – 2C_6_H_10_O_5_]^+^  [SPB d18:1 – 2H_2_O + H]^+^ | [M ‒ H]^‒^ | [Acyl + C_2_H_4_N – H]^–^  [C_11_H_17_NO_8_ – H]^‒^ |
| SP0602 | SHexCer-AS | SHexCer d18:1/18:0(OH) | [M + H]^+^ | [SPB d18:1 – 2H_2_O + H]^+^ | [M ‒ H]^‒^ | [H_2_O_4_S – H]^‒^ |
| SP0602 | SHexCer-NS | [*d*_7_] SHexCer d18:1/13:0 | [M + H]^+^ | [SPB d18:1 – 2H_2_O + H]^+^ | [M ‒ H]^‒^ | [H_2_O_4_S – H]^‒^ |
| ST0101 | Chol | ST 27:1;O | [M + H ‒ H_2_O]^+^ | [C_27_H_45_]^+^ | - | - |
| ST0102 | CE | CE 16:0 | [M + NH_4_]^+^ | [C_27_H_45_]^+^ | - | - |
| ST0505 | AHexCS | ST 27:1;O;Hex 14:0 | [M + NH_4_]^+^ | [C_27_H_45_]^+^ |  | [Acyl FA – H]^‒^ |

^a^ https://www.lipidmaps.org/
